# Synthesis of cycloiptycenes from carbon nanobelts†
†Electronic supplementary information (ESI) available. CCDC 1997770–1997773. For ESI and crystallographic data in CIF or other electronic format see DOI: 10.1039/d0sc02501a


**DOI:** 10.1039/d0sc02501a

**Published:** 2020-06-04

**Authors:** Hiroki Shudo, Motonobu Kuwayama, Yasutomo Segawa, Kenichiro Itami

**Affiliations:** a Graduate School of Science , Nagoya University , Chikusa , Nagoya , 464-8602 , Japan . Email: ysegawa@nagoya-u.jp ; Email: Itami@chem.nagoya-u.ac.jp; b JST-ERATO , Itami Molecular Nanocarbon Project , Nagoya University , Chikusa , Nagoya , 464-8602 , Japan; c Institute of Transformative Bio-Molecules (WPI-ITbM) , Nagoya University , Chikusa , Nagoya , 464-8602 , Japan; d Institute for Molecular Science , Myodaiji , Okazaki , 444-8787 , Japan; e Department of Structural Molecular Science , SOKENDAI (The Graduate University for Advanced Studies) , Myodaiji , Okazaki , 444-8787 , Japan

## Abstract

The synthesis of each of the cycloiptycene derivatives was achieved in one step from the (6,6)carbon nanobelt. It was revealed that the carbon nanobelt reacted as a diene in the Diels–Alder reaction with arynes and alkynes. The structures of all products were identified by X-ray crystallography to confirm that the Diels–Alder reactions took place at the six central benzene rings of the carbon nanobelt. DFT calculations indicated that the release of strain energy is the driving force to promote the Diels–Alder reaction. By using this method, we have successfully synthesized cyclotetracosiptycene, the largest iptycene ever synthesized.

## Abstract 2

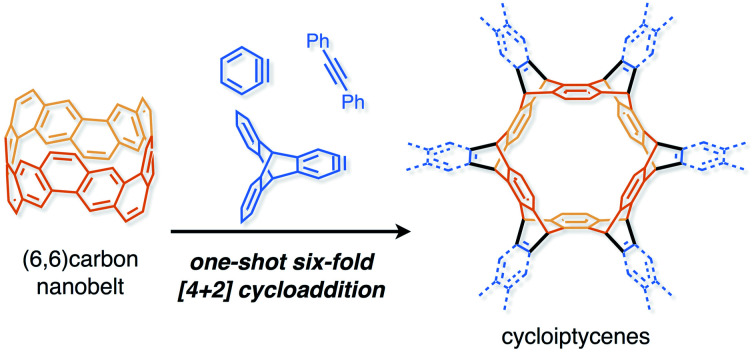
The synthesis of each of the cycloiptycene derivatives was achieved in one step from the (6,6)carbon nanobelt.

## Introduction

The bottom-up synthesis of nanocarbon structures has been of great interest in recent years. Nanocarbons such as fullerenes, carbon nanotubes (CNTs), and graphene have been known as potentially useful functional materials.^1^–^3^ In addition to these well-known nanocarbons, there are many nanocarbon structures that are predicted theoretically but not synthesized yet.^4^,^5^ Since the properties of nanocarbons greatly depend on their structures, precise synthesis methods for various nanocarbon structures are required. The bottom-up synthesis methods using small molecules having substructures of nanocarbons, or so-called molecular nanocarbons, have attracted much attention.^6^–^11^

**Fig. 1:**
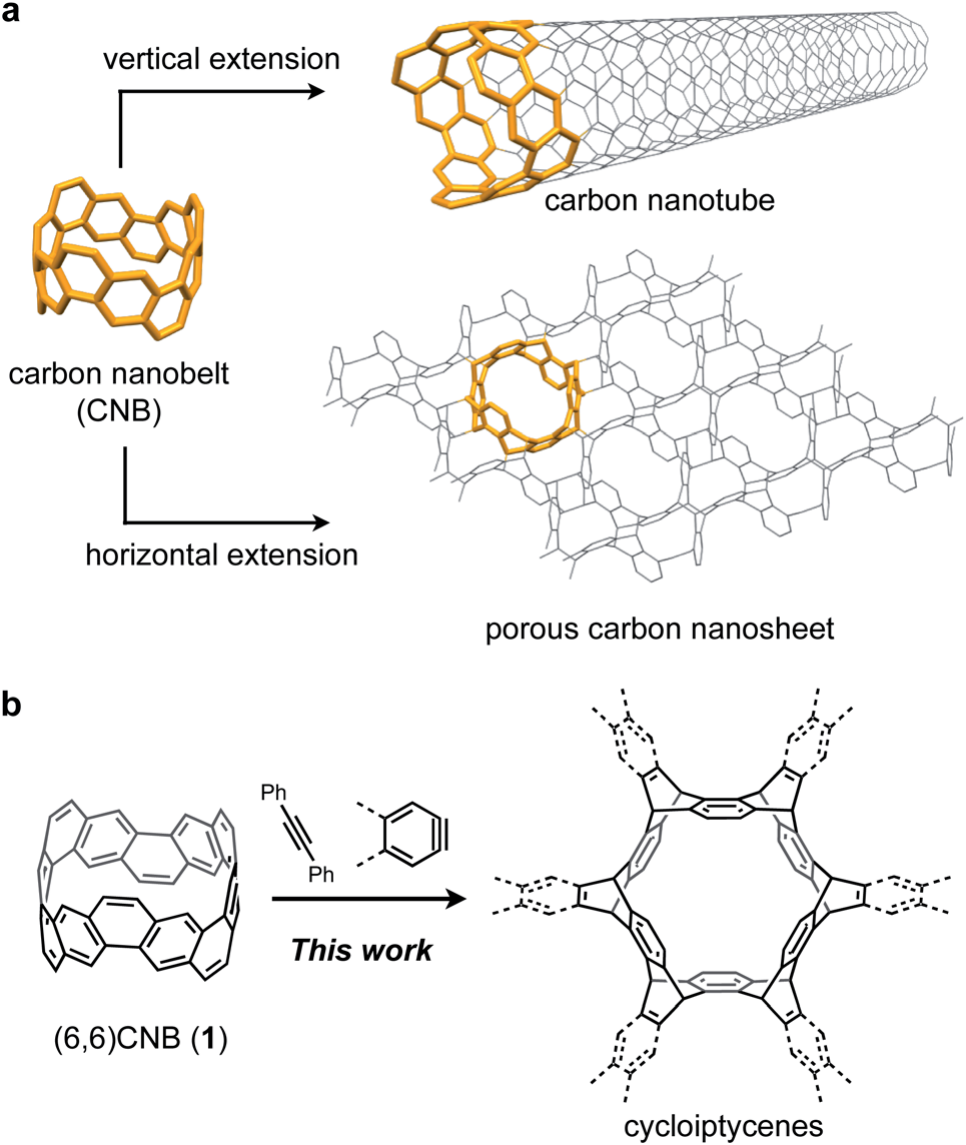
(a) Carbon nanobelts (CNBs) as building blocks of nanocarbon structures. (b) Six-fold Diels–Alder reaction of the (6,6)CNB (**1**) with arynes and alkynes.

Carbon nanobelts (CNBs) are molecular nanocarbons having partial structures of CNTs. In a CNB, all benzene rings are fused together to form a tubular structure.^12^ More than 60 years have passed since it was first theoretically proposed in 1954,^13^ and in 2017 we successfully synthesized the (6,6)CNB (**1**, Fig. 1a).^14^ Our synthetic method was also applicable to the synthesis of large CNBs such as (8,8) and (12,12)CNBs.^15^ Later, Miao and co-workers synthesized chiral CNBs by a different method.^16^ Now the (6,6)CNB is commercially available,^17^ and further investigations and applications are expected including physical properties and host–guest chemistry.^18^

To clarify the usefulness of CNBs as building blocks of nanocarbons, it is necessary to investigate the reactivity of CNBs. Since the extension of carbon nanotubes using seed molecules has been reported,^19^–^21^ CNBs are considered to be effective building blocks (Fig. 1a). On the other hand, by extending CNBs in the horizontal direction, it is conceivable to construct a two-dimensional porous nanocarbon sheet. This corresponds to the functionalization of the sidewall of CNTs at the molecular level.^22^,^23^ However, the reactivity of CNBs remains unclear because there is no report on the reaction of CNBs.

Herein we report the synthesis of sp^2^–sp^3^ macrocyclic hydrocarbons with high symmetry by a six-fold Diels–Alder reaction of the (6,6)CNB (**1**) with arynes and alkynes (Fig. 1b). The structural and electronic properties of the thus-obtained compounds were investigated by X-ray crystallography and photophysical measurements. The characteristic reactivity of **1** was analyzed by using DFT calculations.

## Results and discussion

### Synthesis and structures of cycloiptycenes

The reactivity of **1** with arynes and alkynes was investigated because an elongation reaction of CNT segments with alkynes and arynes was proposed.^24^,^25^ The results are summarized in Table 1. In all reactions (entries 1–4), no elongation product was observed and only side-on products were obtained, in which **1** reacted as a diene in the Diels–Alder reaction. Benzyne generated from *o*-aminobenzoic acid with isoamyl nitrite was reacted with **1** to afford cyclododeciptycene **2** in 13% yield (entry 1). The yield of **2** was slightly decreased to 11% when 2-(trimethylsilyl)phenyl triflate and cesium fluoride were used as the precursor of benzyne (entry 2). The dodecamethoxycyclododeciptycene **3** was also formed from the reaction of **1** with 2-amino-4,5-dimethoxybenzoic acid and isoamyl nitrite in 18% yield. The reaction of **1** with the excess amount of diphenylacetylene took place at 200 °C for three days to form six-fold addition product **4** in 5% yield. In contrast, fullerene C_60_ was not a suitable dienophile^26^ for **1** even under harsh conditions (200 °C, 7 days, entry 5).

**Table 1:** Six-fold Diels–Alder reaction of **1** with arynes and alkynes

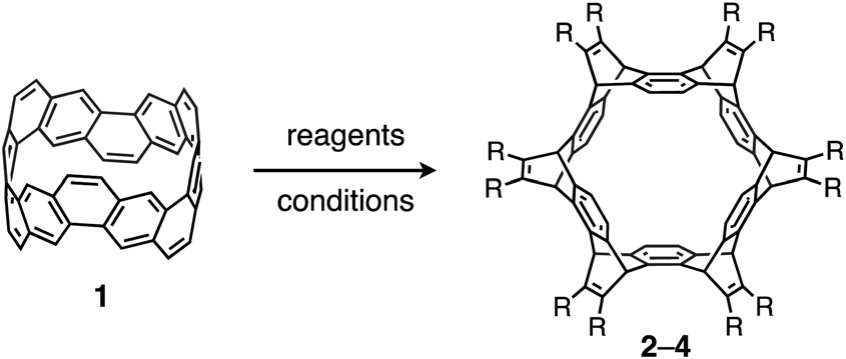			
Entry	Reagents	Conditions	Yield
1	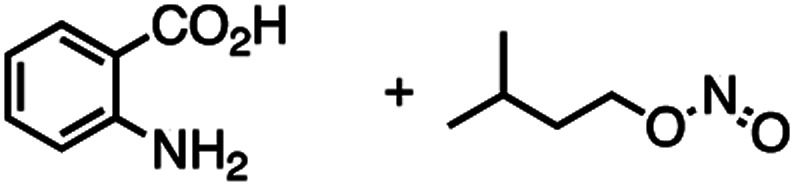	THF/C_2_H_4_Cl_2_, 85 °C, 7 days	**2**, 13%
2	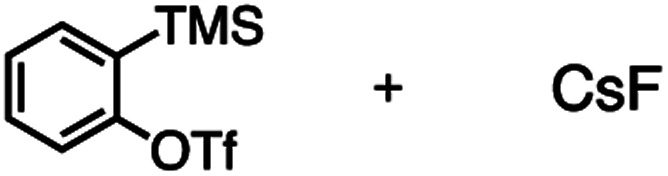	MeCN/C_2_H_4_Cl_2_, 100 °C, 7 days	**2**, 11%
3	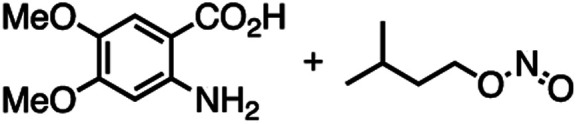	THF/C_2_H_4_Cl_2_, 85 °C, 7 days	**3**, 18%
4	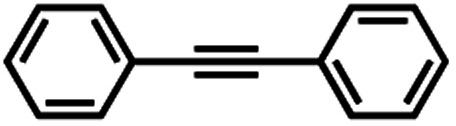	Neat, 200 °C, 3 days	**4**, 5%
5	Fullerene C_60_	*o*-Dichlorobenzene, 200 °C, 7 days	n.d.

**Fig. 2:**
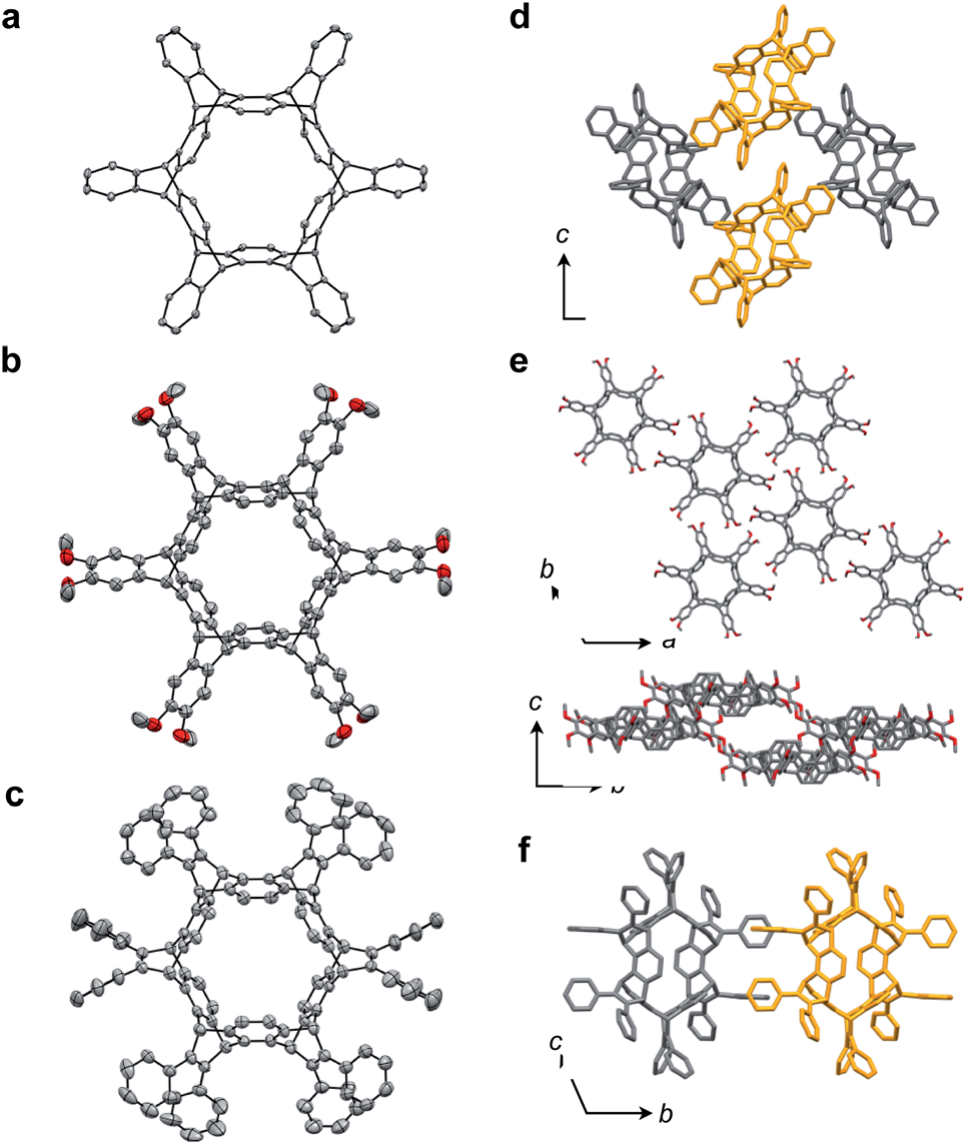
(a–c) ORTEP drawings of **2** (a), **3** (b), and **4** (c) with 50% thermal probabilities: all hydrogen atoms and solvent molecules are omitted for clarity. (d–f) Packing structures of **2** (d), **3** (e), and **4** (f).

X-ray crystallography was performed for the products **2–4**. For recrystallization of **2** and **3**, CHCl_3_/pentane and 1,4-dioxane/hexane solvent systems were used, respectively. Single crystals of **4** were obtained by using THF and pentane. In all compounds, the Diels–Alder reactions occurred at the six central benzene rings of **1**, which correspond to the 9,10-positions of the anthracene subunits of **1** (Fig. 2a–c). In particular, compound **2** is the first example of the synthesized and structurally determined cycloiptycene, the macrocyclic molecule consisting solely of triptycene units.^27^ Considering that multiple steps were required to synthesize the quinone derivative of cycloiptycene,^28^ it was demonstrated that **1** is a useful building block for such complex nanocarbon skeletons. The packing structures of **2–4** are shown in Fig. 2d–f. A gear-like packing structure was found for **2** whereas **3** aligned in the direction of the *c* axis. In the packing of **4**, CH–π interactions between phenyl groups were observed.

### DFT calculations

**Fig. 3:**
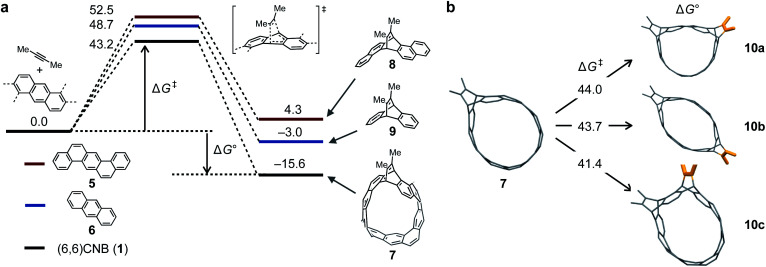
(a) Energy diagrams of the Diels–Alder reactions of **1**, **5** and **6** with 2-butyne (Δ*G*, kcal mol^–1^). (b) Three possible pathways for the second Diels–Alder step for the reaction of **1** with 2-butyne (Δ*G*, kcal mol^–1^). All calculations were performed by using the B3LYP/6-31G(d) level of theory.

The reaction was analyzed by DFT calculations. First, the ground states (GSs) and transition states (TSs) for the first and second addition steps were determined at the B3LYP/6-31G(d) level of theory (Fig. 3a). For dienophile, 2-butyne was used instead of diphenylacetylene to reduce the calculation cost. The TS corresponding to a concerted Diels–Alder reaction (**TS_1–7_**) was obtained with an energy barrier of 43.2 kcal mol^–1^ (Δ*G*^‡^) and the heat of formation from **1 to 7** was determined to be –15.6 kcal mol^–1^ (Δ*G*°). For comparison, the same calculations were performed for [*a*,*h*]dibenzoanthracene **5** and anthracene **6**, the partial structures of **1**, to reveal that both TSs and GSs are higher than those in the case of **1**. Judging from the strain energies of **1** and **7** (119.5 and 89.0 kcal mol^–1^, respectively. See the ESI† for details), the strain release contributes to the Diels–Alder reaction of **1**. In the second step, **10a** is the most stable intermediate of **10a–c**. Because the difference in the strain energies of **10a–c** is small, the number of Clar sextets (**10a**: 5, **10b**: 4, and **10c**: 4) may affect the stability of these products (see the ESI† for details).

### Synthesis and photophysical properties of a large cycloiptycene

**Fig. 4:**
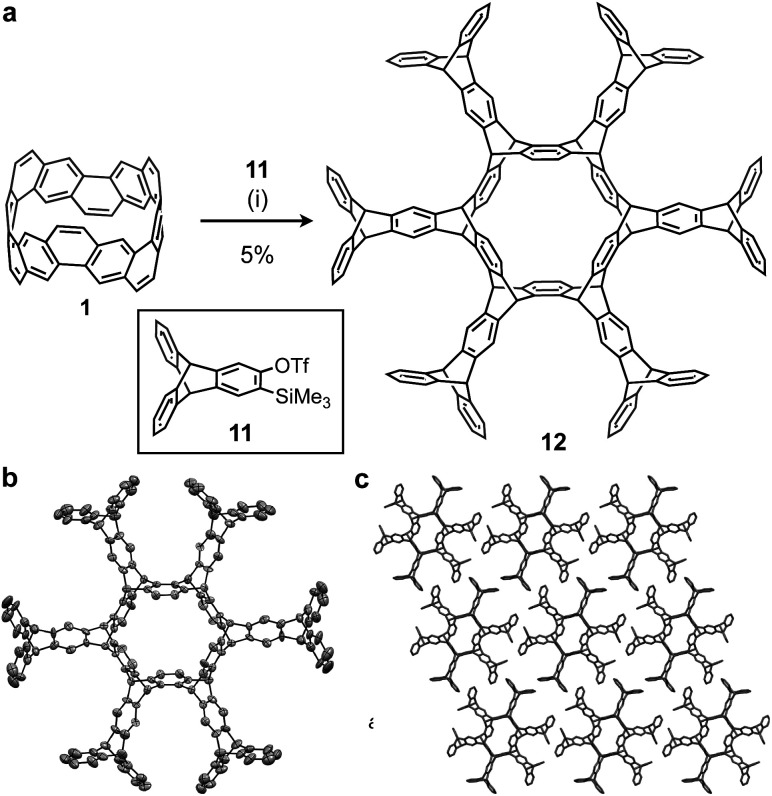
(a) Synthesis of cyclotetracosiptycene **12**. Reaction conditions: (i) **1** (1.0 equiv.), **11** (15 equiv.), CsF (30 equiv.), MeCN/1,2-dichloroethane, 85 °C, 7 days. (b) ORTEP drawings of **12** with 50% thermal probabilities: all hydrogen atoms and solvent molecules are omitted for clarity. (c) Packing structure of **12**.

Using this method, we attempted to synthesize a large cycloiptycene. Compound **11** was prepared according to a previous report^29^ and used as the precursor for triptycene-based aryne. By reaction of **1** with **11** and cesium fluoride at 80 °C for 7 days in acetonitrile and 1,2-dichloroethane, the six-fold triptycene adduct **12** was obtained in 5% isolated yield (Fig. 4a). The product was identified by ^1^H NMR and HRMS. Furthermore, although **12** had low solubility, a good single crystal was obtained by using CH_2_Cl_2_ and pentane, and the structure of **12** was successfully determined by X-ray crystallography. As shown in Fig. 4b, **12** also belongs to a class of cycloiptycenes, and could be named tetracosiptycene according to the number of benzene rings in triptycene moieties.^30^ To the best of our knowledge, tetracosiptycene is the largest iptycene among the previously reported iptycenes.^31^ In the packing structure, **12** aligned two-dimensionally in the *ac* plane.

**Fig. 5:**
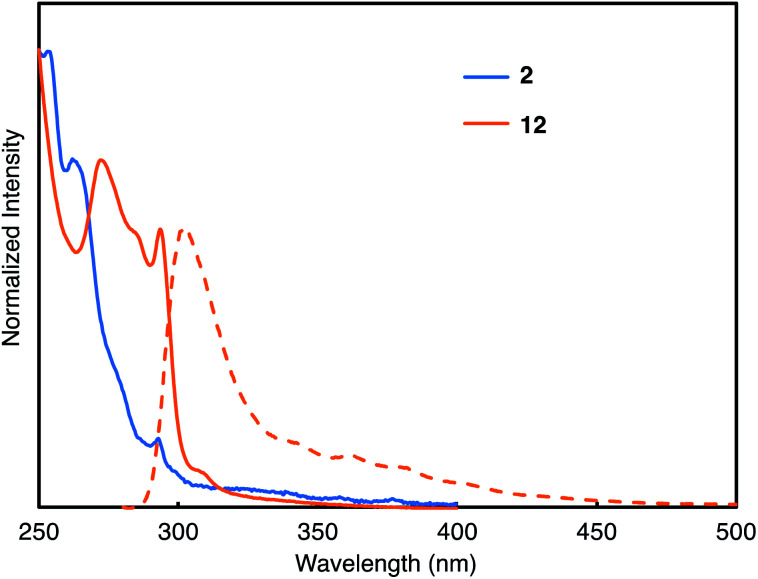
UV-Vis absorption (solid lines) and fluorescence (broken lines) spectra of the diluted dichloromethane solution of **2** (blue) and **12** (orange). The fluorescence spectrum of **12** was recorded upon excitation at 270 nm.

In order to analyze the optoelectronic properties of the thus-obtained iptycene derivatives, the UV-vis absorption spectrum, fluorescence spectrum, fluorescence quantum yield, and fluorescence lifetime were measured (Fig. 5 and the ESI†). The characteristic peaks were observed at 293 nm in the absorption spectra of both **2** and **12**. A broadened absorption band of **12** appeared in the region of 270–300 nm presumably caused by the through-space conjugation of triptycene moieties. Weak fluorescence with a peak top at 302 nm was observed from the solution of **12**, whereas fluorescence of **2** could not be found under UV light (254 nm). The fluorescence quantum yield (*Φ*_F_) and fluorescence lifetime (*τ*) of **12** were 1.2% and 1.56 ns, respectively. According to the equations *Φ*_F_ = *k*_r_ × *τ* and *k*_r_ + *k*_nr_ = *τ*^–1^, the radiative (*k*_r_) and nonradiative (*k*_nr_) decay rate constants from the singlet excited state were determined (*k*_r_ = 7.7 × 10^6^ s^–1^; *k*_nr_ = 1.1 × 10^8^ s^–1^), which indicated that non-radiative decay is relatively fast.

## Conclusions

In conclusion, we have synthesized cycloiptycene derivatives **2**, and **3**, and a related molecule **4** each in one step from the (6,6)CNB **1**. It was revealed that **1** reacts as a diene in the Diels–Alder reaction with arynes and alkynes. The structures of all products were identified by X-ray crystallography to confirm that the Diels–Alder reactions took place at the six central benzene rings of **1**, whose reactivity was similar to that of anthracene. DFT calculations indicated that the release of strain energy promotes the Diels–Alder reactions of **1**. By using this method, we have successfully synthesized tetracosiptycene **12**, the largest iptycene ever synthesized. This study demonstrates the potential utility of molecular nanocarbons such as CNBs as building blocks for novel nanocarbon structures. Further investigations on the construction of nanocarbons from CNBs and other molecular nanocarbons are now ongoing.

## Conflicts of interest

There are no conflicts to declare.

## Supplementary Material

Supplementary information

Crystal structure data
