# Supplementary material for: Synthesis of cycloiptycenes from carbon nanobelts
Source: Chem Sci. 2020 Jun 4;11(26):6775–9. doi: 10.1039/d0sc02501a (PMC7450714; doi:10.1039/d0sc02501a)

Electronic Supplementary Material (ESI) for Chemical Science.
This journal is © The Royal Society of Chemistry 2020

Supporting Information

Synthesis of cycloiptycenes from carbon nanobelt

Hiroki Shudo,[1] Motonobu Kuwayama,[2,3] Yasutomo Segawa*,[1,2,4,5] Kenichiro Itami*,[1,2,3]

[1] *Graduate School of Science, Nagoya University, Nagoya 464-8602, Japan*
[2] *JST, ERATO, Itami Molecular Nanocarbon Project, Nagoya University, Nagoya 464-8602, Japan*
[3] *Institute of Transformative Bio-Molecules (WPI-ITbM) Nagoya University, Nagoya 464-8602, Japan*
[4] *Institute for Molecular Science, Myodaiji, Okazaki, 444-8787, Japan*
[5] *Department of Structural Molecular Science, SOKENDAI (The Graduate University for Advanced Studies), Myodaiji, Okazaki, 444-8787, Japan*

E-mail: ysegawa@nagoya-u.jp, itami@chem.nagoya-u.ac.jp

Table of Contents

1. Experimental Section S2–S6
2. X-ray Crystallography S7
3. Photophysical Measurement S8
4. Computational Study S9–S26
5. References S27
6. ^{1}H and ^{13}C NMR Spectra of Products S28–S33

1. Experimental Section

General

Unless otherwise noted, all materials were obtained from commercial suppliers and used without further purification. (6,6)Carbon nanobelt (**1**) purchased from Tokyo Chemical Industry Co., Ltd. (product no. I1078) includes two equivalent of THF as co-crystallized solvent, and was used without further purification. All reactions were performed with dry solvents under an atmosphere of nitrogen in dried glassware with standard vacuum-line techniques. Work-up and purification procedures were carried out with reagent-grade solvents under air. Compound **11**[S1] was synthesized according to reported procedure.

Analytical thin-layer chromatography (TLC) was performed using E. Merck silica gel 60 F254 precoated plates (0.25 mm). The developed chromatograms were analyzed by UV lamp (254 or 365 nm). Flash column chromatography was performed with KANTO Silica Gel 60N (spherical, neutral, 40-100 μm) or Biotage Isolera® equipped with Biotage SNAP Cartridge KP-Sil columns. Preparative recycling gel permeation chromatography (GPC) was performed with a JAI LC-9260II NEXT instrument equipped with JAIGEL-2HR columns (20 mm I.D. × 600 mm × 2) using $CHCl_3$ as an eluent. Preparative thin-layer chromatography (PTLC) was performed using Wako-gel® B5-F silica coated plates (0.75 mm) prepared in our laboratory. The high-resolution mass spectra (HRMS) were obtained from a JEOL JMS-S3000 SpiralTOF (MALDI-TOF MS). Melting points were measured on a MPA100 Optimelt automated melting point system. Nuclear magnetic resonance (NMR) spectra were recorded on a JEOL ECS-600 (^{1}H 600 MHz, ^{13}C 150 MHz) spectrometer or a JEOL ECA 600II spectrometer with UltraCool™ probe (^{1}H 600 MHz, ^{13}C 150 MHz). Chemical shifts for ^{1}H NMR are expressed in parts per million (ppm) relative to $CHDCl_2$ (δ 5.32 ppm). Chemical shifts for ^{13}C NMR are expressed in ppm relative to CD_2Cl_2 (δ 53.8 ppm). Data are reported as follows: chemical shift, multiplicity (s = singlet, d = doublet, t = triplet, dd = doublet of doublets, td = triplet of doublets, m = multiplet, br = broad signal), coupling constant (Hz), and integration.

Synthesis of cyclododeciptycene 2

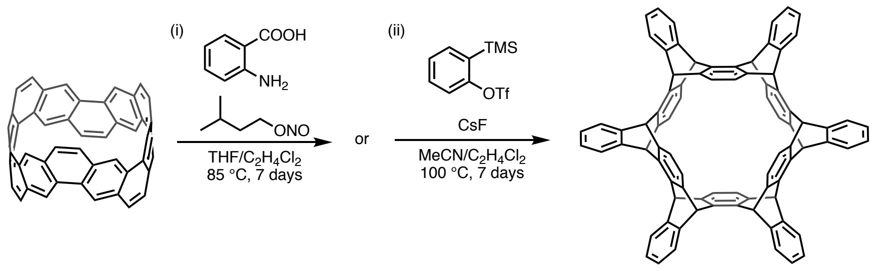

2

Method (i)

To a 20-mL Schlenk tube containing a magnetic stirring bar and filled by argon gas were added **1**·2THF (5.0 mg, 6.7 µmol), 2-aminobenzoic acid (17.8 mg, 130 µmol), isoamyl nitrite (15.2 mg, 130 µmol), THF (1.0 mL) and 1,2-dichloroethane (2.0 mL). The reaction mixture was stirred at 85 °C for 7 days. After cooling the reaction mixture to room temperature, the reaction mixture was quenched by $NaHCO_3$ aqueous solution and $Na_2S_2O_3$ aqueous solution. And the organic layer was extracted with $CHCl_3$, washed with brine, dried over Na_2SO_4, and then evaporated *in vacuo*. The crude material was purified by GPC (eluent: $CHCl_3$), PTLC (eluent: $CHCl_3$/hexane = 7:1), and recrystallization ($CHCl_3$/hexane) to afford **2** (0.9 mg, 13%) as colorless solid.

Method (ii)

To a 20-mL Schlenk tube containing a magnetic stirring bar and filled by argon gas were added **1** (3.0 mg, 4.0 µmol), 2-(trimethylsilyl)phenyl trifluoromethanesulfonate (14.3 mg, 48.0 µmol), cesium fluoride (12.1 mg, 80.0 µmol), acetonitrile (1.0 mL) and 1,2-dichloroethane (2.0 mL). The reaction mixture was stirred at 100 °C for 7 days. After cooling the reaction mixture to room temperature, the reaction mixture was quenched by $NaHCO_3$ aqueous solution and $Na_2S_2O_3$ aqueous solution. And the organic layer was extracted with $CHCl_3$, washed with brine, dried over Na_2SO_4, and then evaporated *in vacuo*. The crude material was purified by GPC column chromatography (eluent: $CHCl_3$), PTLC (eluent: $CHCl_3$/hexane = 7:1), and recrystallization ($CHCl_3$/hexane) to afford **2** (0.5 mg, 11%) as colorless solid.

^{1}H NMR (600 MHz, CD_2Cl_2) δ 7.37 (dd, *J* = 5, 3 Hz, 12H), 6.95 (dd, *J* = 5, 3 Hz, 12H), 6.87 (s, 12H), 5.68 (s, 12H); ^{13}C NMR (150 MHz, CD_2Cl_2) δ 145.0 (4°), 142.1 (4°), 139.5 (4°), 125.5 (CH), 123.9 (CH), 120.9 (CH), 50.1 (CH); HRMS (MALDI-TOF MS) *m/z* calcd for $C_{84}H_{48}$ $[M]^+$: 1056.3751, found: 1056.3765.

Synthesis of 3

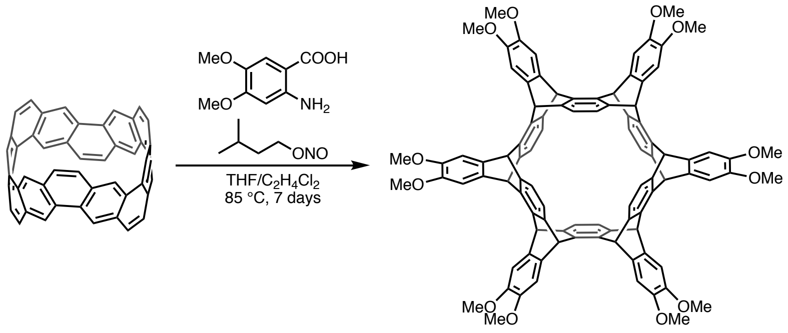

To a 20-mL Schlenk tube containing a magnetic stirring bar and filled by argon gas were added **1**·2THF (5.0 mg, 6.7 µmol), 2-amino-4,5-dimethoxybenzoic acid (26 mg, 0.13 mmol), 3-methylbuthyl nitrile (15.2 mg, 130 µmol), THF (2.0 mL) and 1,2-dichloroethane (1.0 mL). The reaction mixture was stirred at 85 °C for 7 days. After cooling the reaction mixture to room temperature, the reaction mixture was quenched by $NaHCO_3$ aqueous solution and $Na_2S_2O_3$ aqueous solution. And the organic layer was extracted with $CHCl_3$, washed with brine, dried over Na_2SO_4, and then evaporated *in vacuo*. The crude material was purified by GPC column chromatography (eluent: $CHCl_3$), PTLC (eluent: $CHCl_3$/ethyl acetate = 5:1), recrystallization (THF/hexane) to afford **3** (1.7 mg, 18%) as colorless solid.

^{1}H NMR (600 MHz, CD_2Cl_2) δ 7.01 (s, 12H), 6.82 (s, 12H), 5.55 (s, 12H), 3.76 (s, 36H); ^{13}C NMR of **3** could not be obtained due to its low solubility; HRMS (MALDI-TOF MS) *m/z* calcd for $C_{96}O_{12}H_{73}$ $[M+H]^+$: 1417.5097, found: 1417.5080.

Synthesis of 4

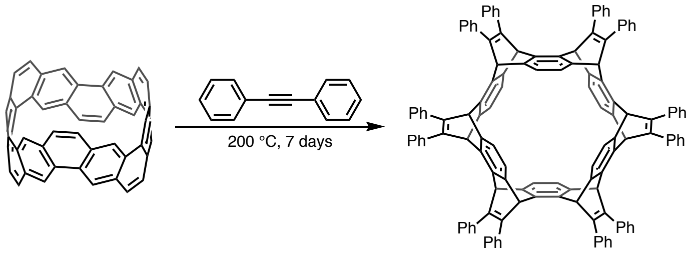

4

To a 20-mL Schlenk tube containing a magnetic stirring bar and filled by argon gas were added **1**·2THF (15.0 mg, 20.1 μmol), diphenylacetylene (300 mg, 1.68 mmol). The reaction mixture was stirred at 200 °C for 7 days. After cooling the reaction mixture to room temperature, the crude material was purified by silica gel column chromatography (eluent: $CHCl_3$) and recrystallization ($CHCl_3$/hexane) to afford **4** (1.6 mg, 5%) as colorless solid.

^{1}H NMR (600 MHz, CD_2Cl_2) δ 7.17 (td, J = 7, 2 Hz, 12H), 7.08 (t, J = 7 Hz, 24H), 6.98 (dd, J = 7, 2 Hz, 24H), 6.84 (s, 12H), 5.63 (s, 12H); ^{13}C NMR (150 MHz, CD_2Cl_2) δ 144.9, 142.0, 139.1, 128.6, 128.2, 127.2, 120.0, 54.4; HRMS (MALDI-TOF MS) *m/z* calcd for C132H84 $[M]^+$: 1668.6568, found: 1668.6570.

Synthesis of cyclotetracosiptycene 12

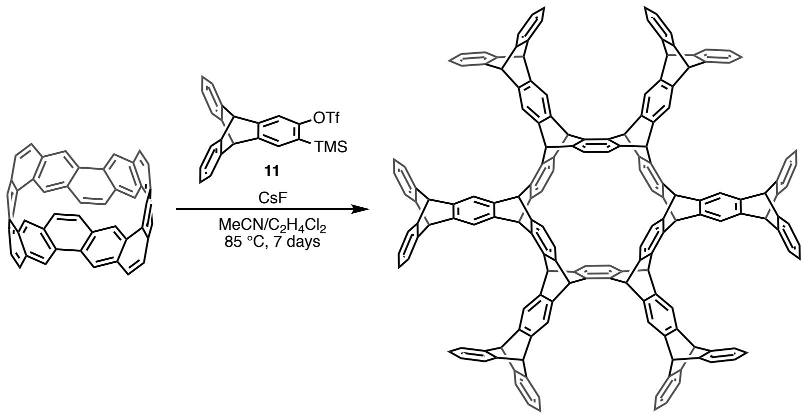

To a 20-mL Schlenk tube containing a magnetic stirring bar and filled by argon gas were added **1**·2THF (10.0 mg, 13.4 μmol), 3-(trimethylsilyl)triptycen-2-yl trifluoromethanesulfonate (**11**, 94.9 mg, 200 μmol), cesium fluoride (60.8 mg, 400 μmol), acetonitrile (1.5 mL) and 1,2-dichloroethane (3.0 mL). The reaction mixture was stirred at 85 °C for 7 days. After cooling the reaction mixture to room temperature, the organic layer was extracted with $CHCl_3$, washed with brine, dried over Na_2SO_4, and then evaporated *in vacuo*. The crude material was purified by PTLC twice ($CHCl_3$/hexane = 8:1, then $CHCl_3$/hexane = 12:1) to afford **12** (1.4 mg, 5%) as colorless solid.

^{1}H NMR (600 MHz, CD_2Cl_2) δ 7.32 (s, 12H), 7.24 (dd, *J* = 7, 2 Hz, 12H), 7.22 (dd, *J* = 7, 2 Hz, 12H), 6.86–6.81 (m, 24H), ;6.60 (s, 12H), 5.39 (s, 12H), 5.23 (s, 12H);^{13}C NMR of **3** could not be obtained due to its low solubility; HRMS (MALDI-TOF MS) *m/z* calcd for $C_{168}H_{96}$ $[M]^+$: 2112.7507, found: 2112.7515.

2. X-ray Crystallography

Details of the crystal data and a summary of the intensity data collection parameters for **2**, **3**, **4**, and **12** are listed in Table S1. A suitable crystal was mounted with mineral oil on a MiTeGen MicroMounts and transferred to the goniometer of the kappa goniometer of a RIGAKU XtaLAB Synergy-S system with 1.2 kW MicroMax-007HF microfocus rotating anode (Graphite-monochromated Mo Kα radiation (λ = 0.71073 Å)) and PILATUS200K hybrid photon-counting detector. Cell parameters were determined and refined, and raw frame data were integrated using CrysAlis^{Pro} (Agilent Technologies, 2010). The structures were solved by direct methods with (SHELXT)[S2] and refined by full-matrix least-squares techniques against F^2 (SHELXL-2018/3)[S3] by using Olex2 software package.[S4] The intensities were corrected for Lorentz and polarization effects. The non-hydrogen atoms were refined anisotropically. Hydrogen atoms were placed using AFIX instructions.

Table S1. Crystallographic data and structure refinement details of **2**, **3**, **4**, and **12.**

	**2**	**3**	**4**	12
CCDC No.	1997770	1997771	1997772	1997773
formula	$C_{96}H_{74}Cl_6$	$C_{74}H_{84}O_{19}$	$C_{142.5}H_{106}O_2$	$C_{201}H_{174}Cl_6$
fw	1440.25	1277.41	1850.27	2802.09
T (K)	123(2)	123(2)	123(2)	123(2)
λ (Å)	0.71073	0.71073	0.71073	0.71073
cryst syst	monoclinic	trigonal	triclinic	monoclinic
space group	$P2_1/n$	R-3	P-1	$C2/c$
a (Å)	13.9419(3)	31.0383(8)	11.0799(9)	46.422(4)
b (Å)	18.9963(3)	31.0383(8)	15.4092(15)	7.7267(6)
c (Å)	14.6207(3)	11.8708(4)	16.833(2)	46.356(4)
α (deg)	90	90	111.731(10)	90
β (deg)	97.9869(19)	90	95.979(8)	109.884(10)
γ (deg)	90	120	99.110(7)	90
V (Å^3)	3834.64(13)	9903.9(5)	2594.1(5)	15636(2)
Z	2	6	1	4
D_{calc} (g·cm^{-3})	1.247	1.285	1.184	1.190
μ (mm^{-1})	0.272	0.092	0.068	0.166
F(000)	1504.0	4080.0	977.0	5928.0
cryst size (mm)	0.10 × 0.10 × 0.03	0.10 × 0.10 × 0.05	0.10 × 0.02 × 0.01	0.05 × 0.01 × 0.01
2θ range (deg)	4.348–61.226	3.75–56.124	3.782–57.25	3.732–53.982
reflns collected	69561	45408	24058	92326
indep reflns/R_{int}	10893 / 0.0385	5068 / 0.0728	10280 / 0.1831	15814 / 0.4476
params	506	285	685	1039
GOF on F^2	1.066	1.510	0.959	1.033
R_1, wR_2 [I>2σ(I)]	0.0732, 0.2204	0.1465, 0.4100	0.1177, 0.2859	0.1677, 0.3823
R_1, wR_2 (all data)	0.0953, 0.2390	0.2302, 0.4520	0.3183, 0.3871	0.4275, 0.4979

3. Photophysical Measurement

UV–Vis absorption spectra were recorded on a Shimadzu UV-3510 spectrometer with a resolution of 0.5 nm. Emission spectra were measured on Shimadzu RF-6000 spectrometer with a resolution of 0.4 nm. Absolute fluorescence quantum yields (Φ_F) were determined on a Shimadzu RF-6000 using a calibrated integrating sphere system upon excitation at 340 nm. For FL lifetime measurement, Hamamatsu Photonics Quantaurus-Tau® fluorescence lifetime spectrometer C11367-21 with LED as a light source was used (excitation: 340 nm, emission: 505 nm, time range: 103 ns, frequency: 2 MHz). Dilute solutions in spectral grade dichloromethane in a 1 cm square quartz cell were used for measurements.

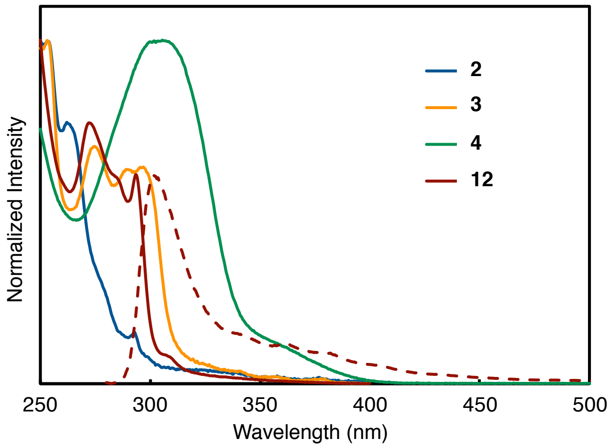

Figure S1. UV–Vis absorption (solid lines) and fluorescence (broken lines) spectra of the diluted dichloromethane solution of **2** (blue), **3** (orange), **4** (green) and **12** (red). Fluorescence spectrum of **12** was recorded upon excitation at 270 nm.

4. Computational Study

The Gaussian 16 program[S5] running on a NEC LX 110Rh system was used for optimization (B3LYP/6-31G(d)[S6,7]. Structures were optimized without any symmetry assumptions. Structures were optimized without any symmetry assumptions. Zero-point energy, enthalpy, and Gibbs free energy at 298.15 K and 1 atm were estimated from the gas-phase studies. Harmonic vibration frequency calculation at the same level was performed to verify all stationary points as local minima (with no imaginary frequency) or transition states (with one imaginary frequency).

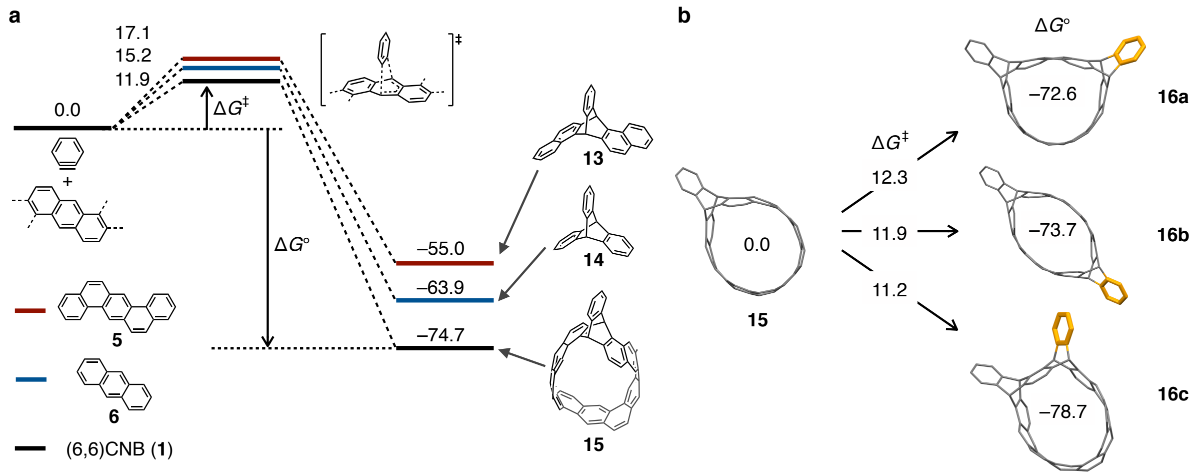

Figure S2. Energy diagrams of the Diels–Alder reactions of **1**, **5**, **6** (a) and **15** (b) with benzyne (Δ*G*, kcal mol^{-1})

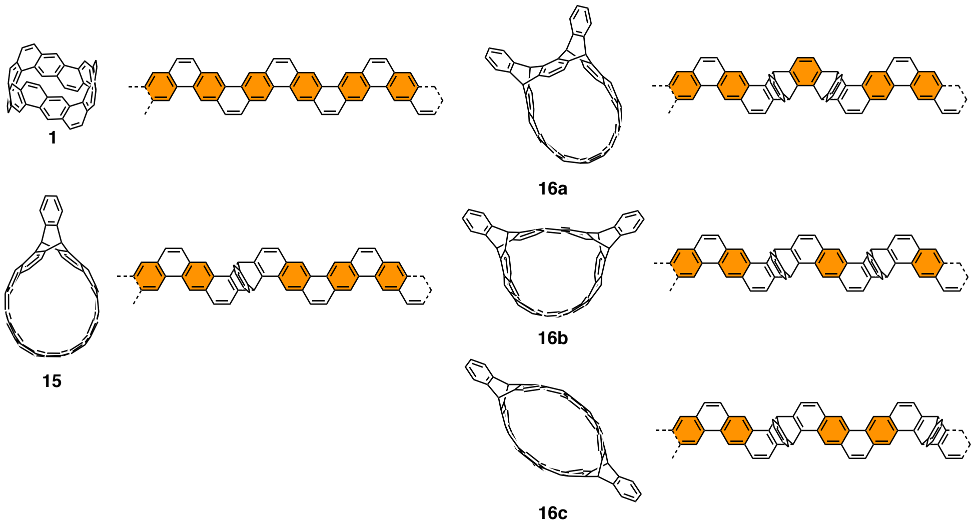

Figure S3. Structures of **1**, **15**, **16a**–**c** and their Clar sextets (orange color).

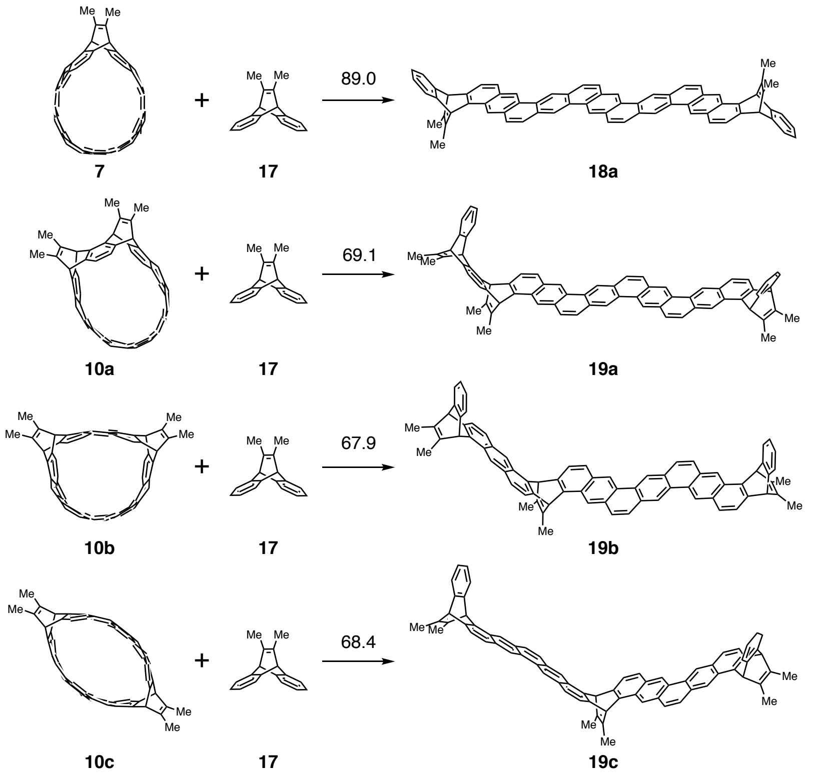

Figure S4. Homodesmotic reactions to estimate the strain energies of **7** and **10a–c** (ΔH, kcal mol^{-1})

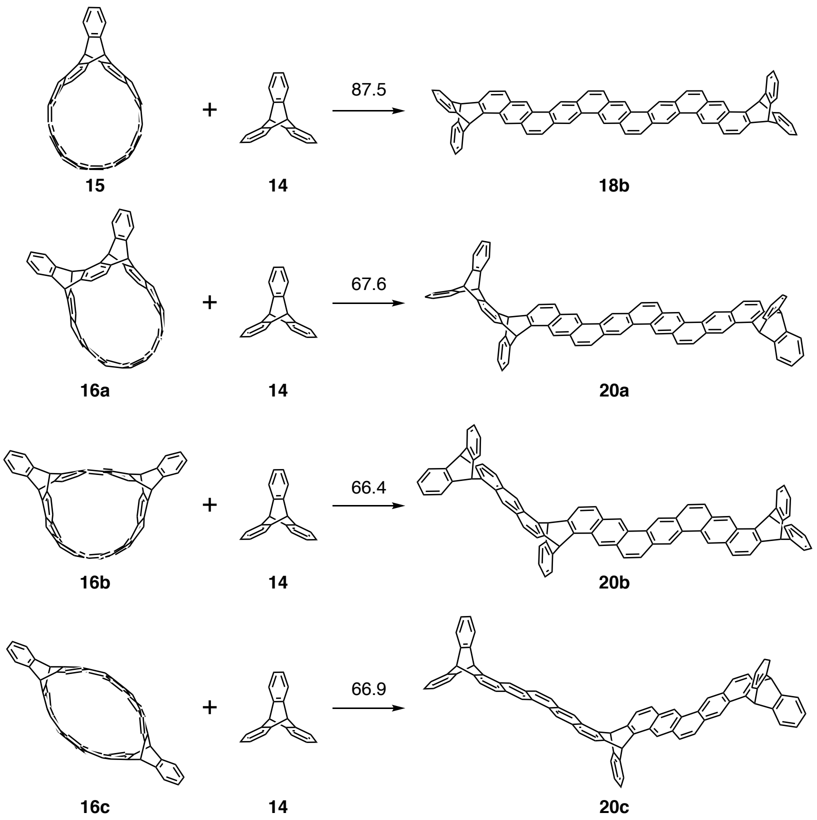

Figure S5. Homodesmotic reactions to estimate the strain energies of **15** and **16a–c** (ΔH, kcal mol^{-1})

Table S2. Uncorrected and thermal-corrected (298 K) energies of stationary points (Hartree).[a]

	E	*E* + *ZPE*	*H*	*G*
**1**	-1843.53941754	-1842.981149	-1842.950508	-1843.036285
**5**	-846.826646448	-846.538113	-846.522340	-846.580046
**6**	-539.530489336	-539.335992	-539.325616	-539.370743
**7**	-1999.57364175	-1998.924158	-1998.888806	-1998.984296
**8**	-1002.82861981	-1002.449533	-1002.429175	-1002.496305
**9**	-695.544193820	-695.259265	-695.244898	-695.298685
**TS$_{1-7}$**	-1999.47275955	-1998.828579	-1998.792050	-1998.890527
**TS$_{5-8}$**	-1002.74569292	-1002.371243	-1002.349809	-1002.419532
**TS$_{6-9}$**	-695.455405878	-695.174926	-695.158897	-695.216317
**10a**	-2155.61450950	-2154.873783	-2154.833764	-2154.938837
**10b**	-2155.60402309	-2154.863530	-2154.823453	-2154.928603
**10c**	-2155.60615436	-2154.865395	-2154.825289	-2154.930666
**TS$_{7-10a}$**	-2155.50983120	-2154.774460	-2154.733228	-2154.841410
**TS$_{7-10b}$**	-2155.50578222	-2154.770395	-2154.729171	-2154.837264
**TS$_{7-10c}$**	-2155.50623802	-2154.770779	-2154.729536	-2154.837741
**13**	-1077.85392083	-1077.483220	-1077.463632	-1077.529604
**14**	-770.572086449	-770.295077	-770.280937	-770.334567
**15**	-2074.59866659	-2073.957592	-2073.923000	-2074.017296
**TS$_{1-15}$**	-2074.44904803	-2073.814789	-2073.778492	-2073.879346
**TS$_{5-13}$**	-1077.72883264	-1077.364153	-1077.342994	-1077.414837
**TS$_{6-14}$**	-770.435567020	-770.164940	-770.149153	-770.208444
**16a**	-2305.66443986	-2304.940552	-2304.902050	-2305.004738
**16b**	-2305.65437351	-2304.930694	-2304.892135	-2304.994912
**16c**	-2305.65626325	-2304.932345	-2304.893755	-2304.996751
**TS$_{15-16a}$**	-2305.50922174	-2304.792212	-2304.751888	-2304.861509
**TS$_{15-16b}$**	-2305.50800327	-2304.790878	-2304.750670	-2304.859713
**TS$_{15-16c}$**	-2305.50819007	-2304.791074	-2304.750839	-2304.860286
**17**	-695.544193820	-695.259265	-695.244898	-695.298685
**18a**	-2695.27582443	-2694.338109	-2694.285821	-2694.424889
**18b**	-2845.32608127	-2844.405215	-2844.354448	-2844.491277
**19a**	-2851.28405135	-2850.255696	-2850.198795	-2850.347604
**19b**	-2851.27180047	-2850.243712	-2850.186822	-2850.335483
**19c**	-2851.27452745	-2850.246320	-2850.189430	-2850.338287
**20a**	-3076.35937503	-3075.356277	-3075.301646	-3075.446978
**20b**	-3076.34767284	-3075.344785	-3075.290187	-3075.435307
**20c**	-3076.35015799	-3075.347153	-3075.292554	-3075.437870

a) *E*: electronic energy; *ZPE*: zero-point energy; H (= $E + ZPE + E_{vib} + E_{rot} + E_{trans} + RT$): sum of electronic and thermal enthalpies; G (= $H - TS$): sum of electronic and thermal free energies.

Table S3. Cartesian coordinates of optimized structures.

1

C	0.124743	4.207427	-2.421157	C	-3.641370	2.033119	0.057864	H	4.247277	0.339421	-2.195167
C	-0.634188	4.108023	-1.192901	C	3.240722	2.603213	1.192848	H	3.553038	2.351449	-2.195199
C	2.207771	3.550690	1.221101	C	-2.207771	3.550690	-1.221101	H	-0.410963	4.369002	-3.353484
C	1.502052	3.890472	0.057818	C	-1.502052	3.890472	-0.057818	H	1.829823	3.847846	2.195159
C	-4.148718	0.714012	-2.421107	C	-2.122777	3.573826	1.192906	H	-4.383968	0.206320	-3.353430

C	1.455913	3.949586	-2.421150	C	2.692617	3.235669	-2.421117	H	2.013225	3.899443	-3.353466
C	-4.156729	0.051401	-1.192867	C	3.581582	2.211837	-2.421143	H	-0.260029	4.252670	2.195135
C	2.122777	3.573826	-1.192905	C	3.874973	1.504828	-1.192907	H	-2.417505	3.508347	-2.195171
C	3.205859	2.684084	-1.221154	C	4.179096	0.136545	-1.221137	H	-4.247278	0.339421	2.195167
C	0.059863	4.169906	0.057816	C	4.120585	0.644467	-0.057851	H	-3.813093	1.901215	-2.195135
C	-0.721682	4.118382	1.221089	C	4.156730	0.051401	1.192867	H	-2.370601	3.693311	3.353421
C	-2.033758	3.625285	1.192863	C	3.927660	1.434239	1.221099	H	-3.989487	1.828824	3.353470
C	-2.618302	3.245993	-0.057843	C	0.721682	4.118382	-1.221090	H	-3.578650	2.540563	-3.353392
C	-1.971199	3.687237	-1.221132	C	4.148719	0.714012	2.421107	H	-3.553037	2.351448	2.195199
C	-4.120585	0.644467	0.057852	C	3.706482	1.995775	2.421089	H	-1.829824	3.847847	-2.195158
C	-4.179096	0.136546	1.221137	C	-1.455912	3.949586	2.421150	H	2.370601	3.693311	-3.353421
C	-3.874973	1.504829	1.192906	C	3.641370	2.033119	-0.057863	H	3.989486	1.828823	-3.353470
C	-3.581480	2.136810	-0.057817	C	2.033758	3.625285	-1.192863	H	3.813093	1.901215	2.195134
C	-3.927660	1.434239	-1.221100	C	3.581480	2.136810	0.057817	H	0.260028	4.252669	-2.195135
C	-2.692617	3.235669	2.421117	C	-0.059862	4.169907	-0.057816	H	4.383969	0.206320	3.353430
C	-3.581583	2.211838	2.421142	C	0.634188	4.108023	1.192900	H	3.578650	2.540562	3.353392
C	-3.706482	1.995775	-2.421088	C	1.971199	3.687237	1.221131	H	-2.013224	3.899442	3.353466
C	-3.240723	2.603213	-1.192848	C	2.618302	3.245993	0.057843	H	2.417505	-3.508347	2.195171
C	-3.205858	2.684083	1.221155	C	-0.124743	4.207427	2.421158	H	0.410964	-4.369003	3.353484

5

C	-3.665107	0.661364	0.299120	C	3.708636	0.740593	0.022479	H	0.079657	-2.408554	-0.558555
C	-3.708636	-0.740593	-0.022479	C	2.493771	1.455764	0.235880	H	-0.079657	2.408554	0.558555
C	-2.493771	-1.455764	-0.235880	C	4.944264	1.416274	0.128985	H	2.467971	-2.385022	-0.648866
C	-1.222250	-0.743692	-0.122553	C	4.997971	2.762977	0.437384	H	4.607176	-1.181044	-0.456669
C	-1.230768	0.654070	0.200092	C	3.803521	3.472948	0.648691	H	5.860585	0.854413	-0.037141
C	-2.485733	1.325431	0.405148	C	2.580787	2.830140	0.549234	H	5.955891	3.269656	0.516006
C	0.021537	-1.352741	-0.312773	C	-4.944264	-1.416274	-0.128985	H	3.836679	4.531591	0.891432
C	1.230768	-0.654070	-0.200092	C	-4.997971	-2.762977	-0.437384	H	1.676262	3.404904	0.717741
C	1.222250	0.743692	0.122553	C	-3.803521	-3.472948	-0.648691	H	-5.860585	-0.854413	0.037141
C	-0.021537	1.352741	0.312773	C	-2.580787	-2.830140	-0.549234	H	-5.955891	-3.269656	-0.516006
C	2.485733	-1.325431	-0.405148	H	-4.607176	1.181044	0.456669	H	-3.836679	-4.531591	-0.891432
C	3.665107	-0.661364	-0.299120	H	-2.467971	2.385022	0.648866	H	-1.676262	-3.404904	-0.717741

6

C	-3.677355	0.517120	0.344381	C	1.203064	0.524099	0.545976	H	-2.406315	-1.809340	-1.814868
C	-3.638116	-0.516970	-0.637008	C	-0.038582	1.018252	0.965016	H	-2.543525	1.809060	1.617271
C	-2.438821	-1.020319	-1.067083	C	2.516220	-1.020480	-0.868812	H	0.068498	-1.807978	-1.713374
C	-1.203064	-0.524099	-0.545976	C	3.677355	-0.517120	-0.344381	H	-0.068498	1.807978	1.713374
C	-1.242802	0.524394	0.447879	C	3.638116	0.516970	0.637008	H	2.543525	-1.809060	-1.617271
C	-2.516220	1.020480	0.868812	C	2.438821	1.020319	1.067083	H	4.637890	-0.904303	-0.673388
C	0.038582	-1.018252	-0.965016	H	-4.637890	0.904303	0.673388	H	4.569332	0.904509	1.041280
C	1.242802	-0.524394	-0.447879	H	-4.569332	-0.904509	-1.041280	H	2.406315	1.809340	1.814868

7

C	3.132565	-0.855456	-2.541843	C	-3.923613	2.623995	-0.109049	H	0.802039	3.861231	-3.191838
C	-1.157724	3.815672	-2.369425	C	-4.742094	-1.406762	0.093892	H	-5.218783	-1.150828	-3.311194
C	-4.122109	-2.711492	-2.363455	C	2.130843	-2.978954	0.989544	H	4.453074	1.463652	-1.854818
C	2.278654	-1.929917	-2.606124	C	3.742734	0.534333	1.310465	H	-3.625132	2.798605	-2.247231
C	0.196231	3.805265	-2.290485	C	0.712597	3.352968	1.384499	H	-1.823165	-4.002246	-2.200674
C	-4.835062	-1.558663	-2.379119	C	-3.457948	3.127451	1.146020	H	0.164284	-3.431360	-2.317780
C	1.921219	-2.630466	-1.418720	C	-4.978935	-0.630677	1.238292	H	2.653236	2.830874	-1.931125
C	4.435445	0.792103	-0.993607	C	-1.974025	-3.632970	1.185234	H	-4.961147	1.087971	-2.223825
C	0.858636	3.551335	-1.026683	C	0.858636	-3.551335	1.026683	H	2.653236	-2.830874	1.931125
C	-3.255548	3.071753	-1.262767	C	4.435445	-0.792103	0.993607	H	0.164284	3.431360	2.317780
C	-4.952424	-0.768665	-1.171836	C	1.921219	2.630466	1.418720	H	-4.961147	-1.087971	2.223825
C	-2.207002	-3.755972	-1.214762	C	-2.207002	3.755972	1.214762	H	4.453074	-1.463652	1.854818
C	0.712597	-3.352968	-1.384499	C	-4.952424	0.768665	1.171836	H	-1.823165	4.002246	2.200674
C	3.742734	-0.534333	-1.310465	C	-3.255548	-3.071753	1.262767	H	-3.625132	-2.798605	2.247231
C	2.130843	2.978954	-0.989544	C	0.196231	-3.805265	2.290485	H	0.802039	-3.861231	3.191838
C	-1.974025	3.632970	-1.185234	C	2.278654	1.929917	2.606124	H	1.761764	2.171171	3.531599
C	-4.978935	0.630677	-1.238292	C	-4.835062	1.558663	2.379119	H	-5.218783	1.150828	3.311194
C	-3.457948	-3.127451	-1.146020	C	-1.157724	-3.815672	2.369425	H	-1.654499	-3.875466	3.335023
C	0.094320	-3.693424	-0.186589	C	3.132565	0.855456	2.541843	H	3.303189	0.228523	3.413720
C	3.634288	-1.355307	-0.202667	C	-4.122109	2.711492	2.363455	H	-3.919146	3.255487	3.282800
C	2.637416	2.380009	0.188656	C	5.852930	-0.432076	0.512519	H	7.005565	2.125377	-1.174714
C	-1.367401	3.815593	0.096556	C	5.852930	0.432076	-0.512519	H	7.005565	-2.125377	1.174715
C	-4.742094	1.406762	-0.093892	C	7.024712	1.028051	-1.233676	H	7.982373	-0.688955	0.828754
C	-3.923613	-2.623995	0.109049	C	7.024712	-1.028051	1.233676	H	7.004115	-0.770169	2.301932
C	-1.367401	-3.815593	-0.096556	H	3.303189	-0.228523	-3.413720	H	7.982373	0.688955	-0.828754

C	2.637416	-2.380009	-0.188656	H	-1.654499	3.875466	-3.335023	H	7.004115	0.770169	-2.301932
C	3.634288	1.355307	0.202667	H	-3.919146	-3.255487	-3.282800				
C	0.094320	3.693424	0.186589	H	1.761764	-2.171171	-3.531599				

8

C	-3.253625	-1.058046	0.995699	C	5.209595	-1.703178	0.306873	H	6.180991	-2.185616	0.373327
C	-2.542108	-1.036058	2.222369	C	4.526352	-1.683130	-0.886126	H	4.952070	-2.149642	-1.771822
C	-1.307146	-0.427469	2.318022	C	-3.417724	-0.478652	-1.388773	H	-3.002055	-0.017630	-2.279603
C	-0.747426	0.184821	1.181092	C	-4.646482	-1.093819	-1.455641	H	-5.191049	-1.114117	-2.395986
C	-1.411162	0.183675	-0.033780	C	-5.209596	-1.703177	-0.306874	H	-6.180992	-2.185615	-0.373328
C	-2.680176	-0.436369	-0.171216	C	-4.526353	-1.683130	0.886125	H	-4.952070	-2.149642	1.771820
C	0.602377	0.902911	1.120378	C	-0.767610	3.496543	-1.412372	H	1.098086	0.922419	2.092433
C	1.411163	0.183674	0.033779	C	-0.319789	2.326908	-0.588791	H	-1.098087	0.922420	-2.092432
C	0.747425	0.184821	-1.181092	C	0.319791	2.326907	0.588793	H	-0.517948	4.455092	-0.948430
C	-0.602377	0.902912	-1.120377	C	0.767613	3.496543	1.412372	H	-1.855157	3.474200	-1.569557
C	2.680175	-0.436370	0.171216	H	-2.988357	-1.511388	3.092681	H	-0.304773	3.476843	-2.409202
C	3.253624	-1.058046	-0.995700	H	-0.768213	-0.418974	3.262487	H	0.517965	4.455090	0.948421
C	2.542108	-1.036057	-2.222369	H	2.988356	-1.511386	-3.092682	H	1.855158	3.474191	1.569572
C	1.307145	-0.427469	-2.318022	H	0.768212	-0.418973	-3.262487	H	0.304762	3.476854	2.409196
C	3.417723	-0.478654	1.388773	H	3.002053	-0.017634	2.279604				
C	4.646481	-1.093821	1.455641	H	5.191048	-1.114121	2.395986				

9

C	3.291210	-1.585688	-0.694324	C	-2.247553	-0.971949	-1.403034	H	-2.245992	-0.977295	-2.490802
C	3.290686	-1.584942	0.699268	C	-1.218907	-0.358077	-0.701003	H	-2.244979	-0.973882	2.494532
C	2.246674	-0.970405	1.406765	C	-1.218545	-0.357083	0.703619	H	-4.101390	-2.063019	1.242861
C	1.218550	-0.357074	0.703621	C	-2.246665	-0.970423	1.406762	H	-4.102205	-2.064432	-1.236888
C	1.218914	-0.358069	-0.701001	C	-3.290672	-1.584966	0.699263	H	0.000346	2.715919	2.623227
C	2.247563	-0.971936	-1.403031	C	-3.291196	-1.585707	-0.694329	H	-0.882752	3.631464	1.400664
C	-0.000001	0.365082	1.282373	H	4.102222	-2.064408	-1.236882	H	0.882292	3.631884	1.400143
C	-0.000003	1.784142	0.672768	H	4.101408	-2.062988	1.242867	H	-0.000004	3.910839	-1.032034
C	-0.000002	1.777931	-0.669123	H	2.244987	-0.973862	2.494536	H	-0.881340	2.959822	-2.240408
C	0.000002	0.361731	-1.280283	H	2.246003	-0.977284	-2.490798	H	0.881307	2.959820	-2.240429
C	-0.000029	3.000657	1.566337	H	-0.000003	0.377217	2.375381				
C	-0.000009	2.965763	-1.583354	H	0.000003	0.376285	-2.373731				

TS_{1-7}

C	-3.232308	1.264947	-2.503897	C	3.851916	-2.668831	-0.049001	H	-0.682307	-4.114669	-3.255918
C	1.248215	-4.061117	-2.364190	C	4.614130	1.414121	0.056051	H	5.003816	1.041678	-3.350392
C	3.970162	2.658517	-2.428517	C	-2.054375	3.164583	1.103099	H	-3.970783	-1.289813	-2.051554
C	-2.503213	2.415919	-2.538863	C	-3.548186	-0.658288	1.245035	H	3.645613	-2.940789	-2.185603
C	-0.107090	-4.054614	-2.335021	C	-0.817476	-3.729786	1.323093	H	1.743303	4.070281	-2.239429
C	4.657567	1.489854	-2.422149	C	3.365379	-3.148726	1.208307	H	-0.346001	3.895610	-2.287208
C	-2.019096	3.000603	-1.317313	C	4.830791	0.662855	1.220465	H	-2.493170	-2.932493	-2.069713
C	-3.889083	-0.726245	-1.124853	C	2.007526	3.831709	1.152450	H	4.810005	-1.145112	-2.193723
C	-0.809908	-3.802798	-1.094115	C	-0.809910	3.802797	1.094114	H	-2.493171	2.932491	2.069711
C	3.256948	-3.198948	-1.204584	C	-3.889083	0.726243	1.124852	H	-0.346000	-3.895610	2.287207
C	4.796744	0.738131	-1.193270	C	-2.019096	-3.000604	1.317312	H	4.810003	1.145114	2.193724
C	2.152281	3.849938	-1.257573	C	2.152282	-3.849937	1.257573	H	-3.970782	1.289811	2.051552
C	-0.817477	3.729785	-1.323094	C	4.796744	-0.738130	1.193272	H	1.743304	-4.070281	2.239429
C	-3.548187	0.658286	-1.245037	C	3.256947	3.198949	1.204585	H	3.645611	2.940790	2.185604
C	-2.054374	-3.164585	-1.103101	C	-0.107092	4.054613	2.335020	H	-0.682309	4.114668	3.255917
C	2.007528	-3.831709	-1.152450	C	-2.503212	-2.415921	2.538861	H	-2.160829	-2.825945	3.485823
C	4.830792	-0.662853	-1.220464	C	4.657566	-1.489853	2.422150	H	5.003815	-1.041677	3.350393
C	3.365378	3.148727	-1.208306	C	1.248212	4.061117	2.364190	H	1.783416	4.124918	3.308654
C	-0.107352	3.977826	-0.145476	C	-3.232307	-1.264948	2.503895	H	-3.487314	-0.741331	3.422409
C	-3.417808	1.398105	-0.054545	C	3.970161	-2.658516	2.428518	H	3.751890	-3.171435	3.362148
C	-2.594776	-2.595125	0.065608	C	-6.068226	0.350725	0.514497	H	-6.482582	-2.140871	-1.609379
C	1.362001	-4.013508	0.111854	C	-6.068228	-0.350723	-0.514493	H	-6.482577	2.140873	1.609383
C	4.614130	-1.414120	-0.056050	C	-6.745815	-1.076048	-1.608218	H	-7.836708	0.999305	1.508204
C	3.851915	2.668832	0.049002	C	-6.745810	1.076050	1.608223	H	-6.472025	0.671260	2.590547
C	1.362000	4.013508	-0.111854	H	-3.487315	0.741330	-3.422411	H	-7.836713	-0.999301	-1.508199
C	-2.594776	2.595122	-0.065609	H	1.783419	-4.124918	-3.308654	H	-6.472030	-0.671257	-2.590543
C	-3.417808	-1.398107	0.054543	H	3.751891	3.171436	-3.362147				
C	-0.107351	-3.977827	0.145475	H	-2.160830	2.825944	-3.485824				

TS_{5-8}

C	1.429363	0.118105	-0.251198	H	0.999340	2.196483	0.219990	C	-3.530550	-1.477432	-0.538115
C	0.567840	1.204703	0.114470	H	-0.999332	-2.196445	0.220445	H	-2.977062	-2.400248	-0.395150
C	-0.821788	1.151787	-0.240385	H	1.118999	-3.295798	-0.422237	C	-5.632839	-0.363532	-0.993205

C	0.821777	-1.151835	-0.240203	H	3.542776	-3.122017	-0.861205	C	-4.889529	-1.542301	-0.776127
C	-1.429375	-0.118155	-0.251116	H	-1.119018	3.295715	-0.422810	H	-5.386946	-2.508173	-0.804665
C	-0.567836	-1.204685	0.114717	H	-3.542813	3.121852	-0.861644	H	-6.700899	-0.421825	-1.184468
C	0.269224	0.564909	2.230929	C	0.825310	1.724314	2.959846	C	4.995862	-0.859898	-0.969419
C	-0.269125	-0.564477	2.231054	H	0.289330	2.649442	2.712074	H	5.557902	-1.775137	-1.141707
C	1.606705	-2.323960	-0.447476	H	0.752707	1.576343	4.045563	C	3.530527	1.477328	-0.538541
C	2.948408	-2.227991	-0.688273	H	1.882512	1.883350	2.713268	H	2.977045	2.400171	-0.395725
C	3.604684	-0.957693	-0.720492	C	-0.825181	-1.723739	2.960219	C	5.632796	0.363342	-0.993511
C	2.847651	0.235837	-0.493699	H	-1.882394	-1.882822	2.713714	C	4.889495	1.542151	-0.776623
C	-1.606724	2.323873	-0.447846	H	-0.289212	-2.648916	2.712606	H	6.700848	0.421599	-1.184831
C	-2.948438	2.227858	-0.688569	H	-0.752535	-1.575556	4.045905	H	5.386912	2.508018	-0.805363
C	-3.604715	0.957554	-0.720521	C	-4.995903	0.859712	-0.969371				
C	-2.847672	-0.235933	-0.493536	H	-5.557951	1.774919	-1.141806				

TS$_{6\text{-}9}$

C	-1.237967	-0.587056	0.711953	C	2.448481	-0.815377	1.404288	H	4.538828	-1.263710	-1.243872
C	0.000000	-0.249608	1.349178	C	3.615642	-1.065263	0.706298	H	2.447792	-0.810699	-2.491641
C	1.237967	-0.587056	0.711953	C	3.615642	-1.065709	-0.705686	C	-0.000001	2.606373	1.921731
C	-1.237963	-0.587517	-0.711645	C	2.448482	-0.816272	-1.403835	H	0.883905	2.347888	2.518531
C	1.237963	-0.587517	-0.711645	H	0.000000	-0.160917	2.434731	H	0.000001	3.695072	1.777085
C	0.000000	-0.250534	-1.349094	H	0.000000	-0.162478	-2.434696	H	-0.883908	2.347891	2.518530
C	0.000000	1.901370	0.624004	H	-2.447792	-0.810699	-2.491641	C	0.000001	2.605379	-1.923152
C	0.000000	1.901092	-0.625053	H	-4.538828	-1.263710	-1.243873	H	0.883908	2.346576	-2.519809
C	-2.448482	-0.816272	-1.403836	H	-4.538828	-1.262924	1.244609	H	-0.883906	2.346575	-2.519810
C	-3.615642	-1.065709	-0.705687	H	-2.447794	-0.809105	2.492090	H	0.000000	3.694160	-1.779095
C	-3.615642	-1.065263	0.706298	H	2.447793	-0.809105	2.492090				
C	-2.448481	-0.815376	1.404288	H	4.538828	-1.262924	1.244610				

10a

C	-2.393494	-0.703330	-2.386808	C	-2.402023	-2.440893	1.295290	H	-2.400529	-3.414390	-1.933581
C	3.511721	-3.202974	-2.397677	C	1.641701	-3.471039	1.382448	H	5.145976	-1.092014	-2.354704
C	2.305430	3.810062	-2.287546	C	5.232311	-1.388770	1.043080	H	-0.321242	3.981679	-1.920695
C	-2.393484	0.703419	-2.386800	C	4.449498	2.546714	1.156088	H	-2.400468	3.414465	-1.933549
C	2.305360	-3.810032	-2.287607	C	0.235147	3.372879	1.413645	H	-0.321312	-3.981621	-1.920752
C	3.511781	3.202987	-2.397622	C	-2.401993	2.440952	1.295320	H	5.145999	1.091996	-2.354685
C	-2.835325	1.379695	-1.264289	C	-3.700447	-1.684121	0.995376	H	-4.103064	1.190106	1.883158
C	-2.771071	-2.892984	-1.049018	C	0.235099	-3.372878	1.413595	H	2.169733	-3.263788	2.308187
C	1.602761	-3.814600	-1.019598	C	4.449458	-2.546784	1.156043	H	4.255596	2.928998	2.154577
C	4.975320	-1.488113	-1.357422	C	5.232332	1.388689	1.043105	H	-4.103074	-1.190021	1.883141
C	4.144813	2.607735	-1.236657	C	1.641752	3.471013	1.382502	H	4.255550	-2.929082	2.154526
C	0.211374	3.846309	-0.983503	C	-1.712780	2.432525	2.528790	H	2.169778	3.263738	2.308239
C	-2.771023	2.893055	-1.048993	C	-0.429618	-2.924802	2.591810	H	-2.179319	1.996278	3.408959
C	-2.835348	-1.379610	-1.264306	C	5.649508	0.677762	2.233861	H	0.131593	-2.879402	3.521991
C	0.211308	-3.846276	-0.983558	C	-0.429582	2.924805	2.591852	H	5.816064	1.238606	3.150396
C	4.144768	-2.607757	-1.236702	C	-1.712803	-2.432491	2.528757	H	0.131624	2.879382	3.522035
C	4.975347	1.488079	-1.357396	C	5.649498	-0.677871	2.233849	H	-2.179329	-1.996241	3.408931
C	1.602826	3.814615	-1.019539	C	-4.687186	-2.704293	0.397550	H	5.816045	-1.238733	3.150374
C	-1.862100	3.050355	0.177377	C	-4.189835	-3.351410	-0.666695	H	-4.258904	-5.335949	-1.499767
C	-3.324469	-0.694115	-0.127144	C	-4.845635	-4.406569	-1.506711	H	-6.583937	-1.912532	1.038133
C	-0.507137	-3.495887	0.182621	C	-6.030704	-2.862472	1.044536	H	-4.919677	4.084053	-2.555200
C	3.710053	-3.015852	0.064315	C	-4.687140	2.704406	0.397579	H	-5.929517	3.157503	2.098336
C	5.328516	0.732711	-0.224453	C	-4.189763	3.351539	-0.666644	H	-6.650591	3.613621	0.546814
C	2.348356	3.553541	0.185620	C	-4.845705	4.406394	-1.506933	H	-6.584868	1.912966	1.035748
C	-0.507082	3.495915	0.182670	C	-6.030767	2.862398	1.044381	H	-5.854377	4.651309	-1.162345
C	-3.324455	0.694197	-0.127132	H	-2.017829	-1.250586	-3.248050	H	-4.256576	5.334255	-1.504154
C	-1.862151	-3.050306	0.177342	H	3.981427	-3.070508	-3.369510	H	-5.856095	-4.648085	-1.164974
C	2.348300	-3.553560	0.185562	H	1.788607	4.181324	-3.169327	H	-4.915222	-4.086508	-2.555957
C	5.328504	-0.732771	-0.224465	H	-2.017811	1.250680	-3.248035	H	-6.651351	-3.612151	0.545668
C	3.710102	3.015813	0.064367	H	1.788525	-4.181264	-3.169395	H	-5.929335	-3.159922	2.097832
C	-3.700420	1.684213	0.995403	H	3.981492	3.070537	-3.369456				

10b

C	-2.563890	-1.897857	2.459069	C	-4.058045	-1.027796	-1.339995	H	-4.978462	-0.636241	1.885797
C	-1.695974	4.383162	2.288038	C	-3.026548	2.959894	-1.462340	H	0.956154	4.552098	2.279905
C	3.790291	-0.431826	2.590893	C	0.837031	4.522209	-1.127558	H	3.555444	-3.084098	1.897103
C	-1.288755	-2.405950	2.472236	C	4.030799	1.929649	-0.929285	H	1.303498	-2.886246	2.120458
C	-2.864262	3.702103	2.198799	C	3.309612	-1.910559	-1.253243	H	-4.407303	1.550627	1.875883
C	3.659055	0.933206	2.666585	C	0.650960	-2.791551	-1.252277	H	2.610736	3.304566	2.403936
C	-0.650950	-2.791555	1.252270	C	-3.869970	-2.509003	-1.023258	H	-1.303488	-2.886241	-2.120465
C	-4.638790	-1.187832	1.006461	C	-3.689313	1.717448	-1.478208	H	-2.610761	3.304570	-2.403936

C	-3.257229	3.089378	0.945271	C	-0.555867	4.562117	-1.270188	H	4.407300	1.550638	-1.875878
C	0.555851	4.562120	1.270189	C	3.257219	3.089384	-0.945268	H	-3.555434	-3.084101	-1.897108
C	3.689299	1.717452	1.478211	C	4.638797	-1.187820	-1.006461	H	-0.956170	4.552093	-2.279904
C	3.869979	-2.508997	1.023254	C	1.288765	-2.405939	-2.472242	H	4.978468	-0.636226	-1.885796
C	0.744952	-2.924643	1.188894	C	-3.659068	0.933206	-2.666584	H	0.703065	-2.403110	-3.388233
C	-3.309604	-1.910570	1.253240	C	2.864250	3.702107	-2.198797	H	-3.390466	1.413052	-3.604424
C	-4.030804	1.929640	0.929289	C	2.563898	-1.897843	-2.459073	H	3.458374	3.499718	-3.086831
C	-0.837046	4.522211	1.127558	C	-3.790298	-0.431828	-2.590893	H	3.000328	-1.475646	-3.361201
C	3.026529	2.959893	1.462342	C	1.695958	4.383160	-2.288037	H	-3.633420	-1.055230	-3.468008
C	4.058047	-1.027790	1.339994	C	-5.216637	-3.002288	-0.460687	H	1.330445	4.736027	-3.249601
C	1.433806	-2.839366	-0.030336	C	-5.639088	-2.282733	0.588583	H	-7.501040	-1.523408	1.357617
C	-2.829638	-2.522884	0.117209	C	-6.898574	-2.442240	1.386224	H	-5.262358	-5.053579	-1.111661
C	-4.124760	1.126911	-0.232492	C	-5.894295	-4.154748	-1.139272	H	5.262264	-5.053466	1.111977
C	-1.399617	4.338008	-0.171804	C	5.639096	-2.282720	-0.588586	H	7.501075	-1.523404	-1.357565
C	2.644877	3.564731	0.270327	C	5.216648	-3.002278	0.460683	H	7.523771	-3.262563	-1.022403
C	4.368969	-0.280354	0.218618	C	5.894324	-4.154714	1.139289	H	6.673902	-2.638007	-2.444238
C	2.829648	-2.522881	-0.117215	C	6.898586	-2.442219	-1.386224	H	6.853465	-4.407035	0.678218
C	-1.433796	-2.839366	0.030329	H	-3.000320	-1.475665	3.361199	H	6.081768	-3.934419	2.199718
C	-4.368966	-0.280363	-0.218616	H	-1.330462	4.736033	3.249602	H	-6.853581	-4.406872	-0.678396
C	-2.644892	3.564729	-0.270326	H	3.633416	-1.055231	3.468006	H	-6.081447	-3.934598	-2.199781
C	1.399602	4.338008	0.171805	H	-0.703056	-2.403120	3.388226	H	-7.523782	-3.262551	1.022370
C	4.124755	1.126919	0.232494	H	-3.458386	3.499714	3.086833	H	-6.673885	-2.638087	2.444227
C	-0.744941	-2.924640	-1.188902	H	3.390447	1.413050	3.604425				

10c

C	-0.678846	-3.424579	2.284787	C	-2.709519	-2.619739	-1.374351	H	-3.232393	-2.614781	2.020397
C	-3.150272	2.019223	2.587778	C	-5.238538	0.764449	-1.014637	H	-0.974992	3.396502	2.324385
C	4.010710	0.950149	2.542500	C	-1.435726	3.260992	-1.057124	H	5.253106	-1.415220	1.892151
C	0.678844	-3.424578	2.284787	C	2.751692	2.790635	-1.061172	H	3.232390	-2.614778	2.020398
C	-4.010706	0.950150	2.542500	C	4.567649	-0.576661	-1.302280	H	-5.253103	-1.415216	1.892154
C	3.150273	2.019219	2.587777	C	2.709518	-2.619733	-1.374350	H	0.974989	3.396491	2.324386
C	1.435723	-3.260992	1.057122	C	-1.455925	-3.257775	-1.362239	H	0.974989	-3.396492	-2.324387
C	-2.751695	-2.790641	1.061171	C	-4.567645	-0.576663	-1.302281	H	-5.253104	1.415214	-1.892156
C	-4.567645	0.576661	1.302279	C	-2.751695	2.790639	-1.061172	H	3.232390	2.614776	-2.020399
C	-1.455925	3.257773	1.362237	C	1.435723	3.260990	-1.057124	H	-0.974992	-3.396503	-2.324387
C	2.709517	2.619732	1.374349	C	5.238540	0.764455	-1.014633	H	-3.232393	2.614779	-2.020399
C	5.238540	-0.764456	1.014632	C	3.150273	-2.019220	-2.587779	H	5.253106	1.415219	-1.892152
C	2.751692	-2.790637	1.061171	C	-4.010706	-0.950152	-2.542501	H	2.672798	-2.309469	-3.520273
C	-1.435725	-3.260993	1.057122	C	0.678844	3.424577	-2.284788	H	-4.227522	-0.368527	-3.435270
C	-5.238537	-0.764451	1.014635	C	4.010710	-0.950150	-2.542501	H	1.224698	3.456278	-3.225059
C	-2.709519	2.619737	1.374349	C	-3.150272	-2.019224	-2.587780	H	4.227528	-0.368526	-3.435270
C	1.455922	3.257766	1.362237	C	-0.678846	3.424577	-2.284789	H	-2.672797	-2.309474	-3.520274
C	4.567649	0.576660	1.302279	C	-6.656384	0.421861	-0.521249	H	-1.224700	3.456279	-3.225059
C	3.369672	-2.318199	-0.125505	C	-6.656384	-0.421864	0.521247	H	-7.805444	-0.720422	2.319043
C	-0.735960	-3.428712	-0.188752	C	-7.828052	-1.000502	1.256412	H	-7.805347	0.720609	-2.319074
C	-4.421985	-1.349365	-0.162746	C	-7.828053	1.000515	-1.256400	H	7.810315	-2.098913	1.220325
C	-3.369674	2.318201	0.125505	C	6.656385	0.421864	-0.521247	H	7.810402	2.098923	-1.220157
C	0.735958	3.428708	0.188750	C	6.656385	-0.421867	0.521245	H	8.785860	-0.668395	0.846019
C	4.421986	1.349362	0.162746	C	7.828053	-1.000559	1.256366	H	7.805482	-0.720540	2.319016
C	4.421986	-1.349363	-0.162747	C	7.828054	1.000572	-1.256355	H	8.785860	0.668278	-0.846111
C	0.735958	-3.428710	-0.188752	H	-1.224700	-3.456280	3.225058	H	7.805397	0.720706	-2.319042
C	-3.369674	-2.318203	-0.125506	H	-2.672797	2.309472	3.520272	H	-8.785859	0.668203	-0.846172
C	-4.421985	1.349363	0.162744	H	4.227527	0.368525	3.435269	H	-7.810449	2.098870	-1.220243
C	-0.735960	3.428710	0.188750	H	1.224698	-3.456280	3.225058	H	-8.785859	-0.668336	0.846067
C	3.369672	2.318197	0.125504	H	-4.227522	0.368525	3.435269	H	-7.810350	-2.098860	1.220433
C	1.455922	-3.257767	-1.362239	H	2.672798	2.309468	3.520272				

TS$_{7\text{-}10a}$

C	-2.773367	-0.471430	-2.460853	C	-2.878401	-2.157967	1.294910	H	-3.180836	-3.140230	-1.918446
C	2.835355	-3.530129	-2.468013	C	0.951069	-3.760873	1.302212	H	4.796541	-1.721944	-2.335914
C	3.134914	3.588683	-2.245856	C	4.799846	-2.157053	1.049049	H	0.542758	4.013160	-1.980554
C	-2.428132	0.871063	-2.468458	C	4.836609	1.880210	1.251690	H	-1.447283	3.323646	-2.068821
C	1.557019	-3.973878	-2.382596	C	0.918727	3.571937	1.389564	H	-1.077532	-3.861531	-1.999992
C	4.219708	2.780774	-2.322019	C	-1.750360	2.665893	1.241276	H	5.346529	0.376763	-2.271086
C	-2.462618	1.607858	-1.269612	C	-4.015752	-1.171297	1.022037	H	-3.427554	1.551198	2.001694
C	-3.415813	-2.549697	-1.030321	C	-0.429846	-3.484879	1.351993	H	1.502760	-3.665989	2.232143
C	0.859772	-3.966500	-1.111936	C	3.843084	-3.179981	1.111013	H	4.683454	2.270632	2.254075
C	4.556461	-2.124809	-1.355878	C	5.339653	0.576954	1.133574	H	-4.283507	-0.598428	1.913051
C	4.668526	2.059110	-1.148704	C	2.321140	3.473331	1.408600	H	3.581309	-3.564179	2.092923
C	1.020254	3.880183	-1.013984	C	-1.143370	2.924524	2.515834	H	2.791305	3.272959	2.367108

C	-1.826550	2.890028	-1.147168	C	-1.002792	-2.980532	2.554465	H	-1.689655	2.669273	3.421081
C	-3.246887	-1.048660	-1.276617	C	5.555085	-0.232782	2.314216	H	-0.429089	-3.048815	3.475470
C	-0.528215	-3.841921	-1.062478	C	0.148682	3.354049	2.586883	H	5.791402	0.261617	3.253427
C	3.551128	-3.097075	-1.283734	C	-2.185149	-2.280503	2.518456	H	0.649944	3.435400	3.548414
C	5.225565	0.780208	-1.269468	C	5.301939	-1.563931	2.270776	H	-2.563003	-1.784984	3.409633
C	2.405584	3.703556	-0.999236	C	-5.196741	-2.004035	0.491063	H	5.330287	-2.168438	3.174176
C	-1.126434	3.146226	0.067519	C	-4.871212	-2.753606	-0.572032	H	-5.320873	-4.713395	-1.341804
C	-3.485602	-0.290602	-0.131567	C	-5.738517	-3.696771	-1.350945	H	-6.895288	-0.882834	1.193885
C	-1.195157	-3.464387	0.126984	C	-6.512930	-1.913534	1.203740	H	-3.670753	4.459222	-2.834702
C	3.056501	-3.496172	-0.002528	C	-4.273455	3.624630	0.291820	H	-4.918525	3.746317	2.326521
C	5.343764	-0.058432	-0.149545	C	-3.730548	4.078834	-0.731294	H	-6.095975	4.329760	1.136327
C	3.063785	3.356341	0.227335	C	-3.675220	5.004074	-1.882359	H	-5.821482	2.590014	1.341183
C	0.238124	3.638613	0.129061	C	-5.326042	3.568750	1.323585	H	-4.544142	5.675277	-1.885101
C	-3.024484	1.045079	-0.081804	H	-2.631170	-1.082499	-3.348962	H	-2.771246	5.624937	-1.857400
C	-2.480542	-2.842038	0.160567	H	3.315767	-3.408198	-3.436041	H	-6.757748	-3.751589	-0.958031
C	1.637398	-3.864266	0.096968	H	2.717856	4.047966	-3.138947	H	-5.803076	-3.392932	-2.405321
C	5.034415	-1.491068	-0.194713	H	-2.019072	1.329526	-3.365552	H	-7.277057	-2.555779	0.756775
C	4.278862	2.534982	0.144948	H	0.995818	-4.219182	-3.281177	H	-6.411507	-2.200981	2.259849
C	-2.929543	1.878437	1.091493	H	4.694935	2.571160	-3.277365				

TS$_{7\text{-}10b}$

C	2.166728	-2.533388	-2.395321	C	3.895991	-1.278182	1.270190	H	4.422383	-1.047584	-2.065923
C	2.243757	4.335406	-2.315365	C	3.676793	2.793470	1.365660	H	-0.377487	4.678863	-2.230748
C	-3.729604	-0.083544	-2.578931	C	-0.187190	4.496751	1.168088	H	-3.963489	-2.739492	-1.886865
C	0.890601	-3.009787	-2.362491	C	-3.685252	2.278086	0.945440	H	-1.746643	-3.120547	-2.009526
C	3.343196	3.544076	-2.280844	C	-3.657578	-1.599731	1.272892	H	4.530228	1.180191	-2.045773
C	-3.388600	1.245533	-2.646297	C	-1.141783	-2.795975	1.357170	H	-2.047359	3.464015	-2.359403
C	0.190729	-3.141818	-1.108757	C	3.226818	-2.539570	1.172217	H	0.790785	-2.912308	2.254901
C	4.045303	-1.427441	-1.119009	C	4.201530	1.489894	1.344414	H	3.409696	3.205031	2.334863
C	3.719279	2.881217	-1.048804	C	1.208842	4.452637	1.280915	H	-4.114085	1.941846	1.885896
C	0.044434	4.623172	-1.231330	C	-2.755118	3.317270	0.981250	H	2.970890	-3.032990	2.107431
C	-3.340586	2.030930	-1.459162	C	-4.843115	-0.678806	0.972936	H	1.627654	4.368052	2.279701
C	-4.214260	-2.119460	-1.023310	C	-1.745838	-2.302866	2.551534	H	-5.125252	-0.075122	1.838358
C	-1.207457	-3.113372	-1.065783	C	4.264600	0.729249	2.562907	H	-1.174086	-2.331294	3.475853
C	2.867144	-2.237171	-1.175631	C	-2.274993	3.839219	2.245085	H	4.293072	1.260249	3.511236
C	4.313183	1.616703	-1.074467	C	-2.955006	-1.654335	2.498410	H	-2.873776	3.672199	3.137369
C	1.436196	4.502398	-1.123759	C	4.106414	-0.623777	2.526677	H	-3.356217	-1.155431	3.377380
C	-2.521237	3.175969	-1.426440	C	-1.036825	4.384207	2.337535	H	4.014266	-1.198103	3.445685
C	-4.140723	-0.626358	-1.342746	C	5.061284	-3.689198	0.429255	H	-0.623498	4.658085	3.305498
C	-1.903757	-2.823417	0.126194	C	5.459240	-3.147021	-0.619591	H	6.891160	-2.071110	-1.789279
C	2.356928	-2.707476	0.044977	C	6.382685	-3.042872	-1.768095	H	4.262228	-5.324126	1.553114
C	4.369567	0.818342	0.084781	C	5.161497	-4.698015	1.503265	H	-5.998888	-4.401215	-1.177402
C	2.021436	4.242559	0.154170	C	-5.989720	-1.596143	0.509750	H	-7.732379	-0.536246	1.198553
C	-2.091709	3.735129	-0.228106	C	-5.645793	-2.381760	-0.520698	H	-8.027688	-2.249175	0.857979
C	-4.369858	0.166343	-0.232829	C	-6.473841	-3.411400	-1.229518	H	-7.149083	-1.767212	2.317388
C	-3.235314	-2.298036	0.159273	C	-7.291920	-1.541870	1.251076	H	-7.479750	-3.501527	-0.809764
C	0.950837	-3.073515	0.117534	H	2.646232	-2.305574	-3.344779	H	-6.578145	-3.168593	-2.296458
C	4.214082	-0.623979	0.066601	H	1.877539	4.741357	-3.255373	H	6.023028	-5.358716	1.337770
C	3.231555	3.411111	0.194344	H	-3.636498	-0.726036	-3.451275	H	5.286318	-4.228228	2.486944
C	-0.786883	4.403090	-0.125498	H	0.338217	-3.168593	-3.285521	H	7.152723	-3.824388	-1.720882
C	-3.891276	1.513037	-0.227218	H	3.882778	3.298428	-3.192463	H	5.852853	-3.154171	-2.722449
C	0.246553	-3.016600	1.320771	H	-3.019504	1.675982	-3.573888				

TS$_{7\text{-}10c}$

C	-0.577586	3.707367	-2.289065	C	-2.783898	2.964120	1.280494	H	-3.071059	2.746456	-2.122428
C	-3.353350	-2.464787	-2.503238	C	-4.705958	-0.705074	1.138667	H	-1.110833	-3.800870	-2.300798
C	3.854390	-0.849287	-2.555614	C	-1.372449	-3.523480	1.089664	H	5.148736	1.475062	-1.845819
C	0.778066	3.703135	-2.239369	C	2.762938	-2.929208	0.973626	H	3.268784	2.781926	-1.924490
C	-4.102681	-1.327039	-2.482569	C	4.446806	0.529232	1.315931	H	-4.785948	1.253769	-2.074851
C	2.998593	-1.921261	-2.629392	C	2.613260	2.606755	1.442178	H	0.864909	-3.385230	-2.370956
C	1.475284	3.468735	-0.989603	C	-1.530548	3.597082	1.320322	H	0.864909	3.385230	2.370957
C	-2.665409	2.991976	-1.144406	C	-4.378771	0.683456	1.233206	H	-4.785953	-1.253770	2.074848
C	-4.378770	-0.683457	-1.233208	C	-2.665411	-2.991978	1.144405	H	3.268782	-2.781925	1.924493
C	-1.530547	-3.597083	-1.320322	C	1.475283	-3.468735	0.989605	H	-1.110834	3.800869	2.300799
C	2.613260	-2.606754	-1.442176	C	5.132067	-0.797593	0.989049	H	-3.071062	-2.746458	2.122427
C	5.132068	0.797593	-0.989045	C	2.998593	1.921261	2.629395	H	5.148735	-1.475062	1.845823
C	2.762939	2.929208	-0.973623	C	-4.102683	1.327038	2.482567	H	2.495055	2.165118	3.561532
C	-1.372447	3.523479	-1.089664	C	0.778064	-3.703135	2.239371	H	-4.405490	0.841820	3.407657
C	-4.705955	0.705073	-1.138670	C	3.854389	0.849288	2.555617	H	1.362215	-3.758858	3.155048
C	-2.783898	-2.964121	-1.280496	C	-3.353351	2.464787	2.503236	H	4.040254	0.224216	3.425738

C	1.389733	-3.303210	-1.424959	C	-0.577588	-3.707368	2.289066	H	-3.041269	2.907564	3.445898
C	4.446806	-0.529232	-1.315927	C	-6.880454	-0.344391	0.519071	H	-1.092768	-3.763514	3.245321
C	3.309811	2.357083	0.201249	C	-6.880452	0.344395	-0.519081	H	-7.288279	0.630655	-2.599360
C	-0.732505	3.696753	0.181167	C	-7.562726	1.049663	-1.623132	H	-7.288286	-0.630651	2.599349
C	-4.212970	1.389928	0.025856	C	-7.562732	-1.049659	1.623120	H	7.702974	2.134153	-1.157744
C	-3.317975	-2.533117	-0.020319	C	6.549600	-0.436294	0.509040	H	7.702973	-2.134153	1.157748
C	0.734875	-3.606967	-0.237971	C	6.549600	0.436293	-0.509036	H	8.679122	0.694018	-0.824876
C	4.325671	-1.350583	-0.209164	C	7.721334	1.037327	-1.226014	H	7.699628	0.788614	-2.296449
C	4.325671	1.350583	0.209168	C	7.721333	-1.037328	1.226018	H	8.679122	-0.694019	0.824881
C	0.734875	3.606967	0.237972	H	-1.092765	3.763513	-3.245320	H	7.699627	-0.788615	2.296454
C	-3.317974	2.533115	0.020317	H	-3.041266	-2.907564	-3.445899	H	-8.653270	-0.969859	1.521070
C	-4.212971	-1.389930	-0.025859	H	4.040255	-0.224216	-3.425734	H	-7.303618	-2.115334	1.641644
C	-0.732505	-3.696754	-0.181167	H	1.362217	3.758858	-3.155046	H	-8.653265	0.969864	-1.521084
C	3.309810	-2.357083	-0.201246	H	-4.405487	-0.841820	-3.407660	H	-7.303612	2.115338	-1.641656
C	1.389733	3.303211	1.424961	H	2.495056	-2.165117	-3.561529				

13

C	3.277245	-1.323996	-0.985770	C	-5.233757	-1.954389	-0.322005	H	-3.002636	-0.297120	-2.290893
C	2.572453	-1.301464	-2.216884	C	-4.555119	-1.937127	0.873843	H	-5.201476	-1.370662	-2.411965
C	1.333880	-0.703797	-2.316353	C	3.424959	-0.751715	1.400132	H	-6.209732	-2.427025	-0.390872
C	0.757009	-0.108207	-1.177871	C	4.660146	-1.354145	1.469761	H	-4.988979	-2.395674	1.759640
C	1.413867	-0.109978	0.041990	C	5.233756	-1.954391	0.322006	H	3.002636	-0.297116	2.290893
C	2.691922	-0.714005	0.180285	C	4.555118	-1.937131	-0.873841	H	5.201478	-1.370661	2.411964
C	-0.603481	0.594057	-1.139209	C	0.662164	3.205461	1.239112	H	6.209730	-2.427027	0.390875
C	-1.413867	-0.109978	-0.041990	C	0.331056	2.007850	0.618969	H	4.988977	-2.395680	-1.759639
C	-0.757010	-0.108205	1.177870	C	-0.331055	2.007850	-0.618970	H	1.174812	3.206155	2.198293
C	0.603481	0.594057	1.139209	C	-0.662161	3.205461	-1.239114	H	-1.174809	3.206154	-2.198295
C	-2.691923	-0.714005	-0.180285	C	-0.328591	4.415780	-0.614638	H	-0.584564	5.357237	-1.093307
C	-3.277246	-1.323994	0.985771	C	0.328595	4.415780	0.614636	H	0.584569	5.357237	1.093303
C	-2.572454	-1.301460	2.216886	H	3.028606	-1.766620	-3.087482	H	-1.091737	0.598667	-2.114785
C	-1.333881	-0.703794	2.316354	H	0.801959	-0.690921	-3.264554	H	1.091736	0.598667	2.114785
C	-3.424959	-0.751716	-1.400131	H	-3.028608	-1.766615	3.087483				
C	-4.660147	-1.354146	-1.469760	H	-0.801960	-0.690916	3.264554				

14

C	1.896167	-3.315727	0.697127	C	-3.819955	0.016316	0.697128	H	-0.000256	-0.000018	-2.390870
C	1.896165	-3.315728	-0.697128	C	-2.609882	0.011157	1.404320	H	-0.000256	-0.000020	2.390870
C	1.295297	-2.265358	-1.404306	C	1.314606	2.254211	1.404319	H	-2.610322	0.011138	-2.491956
C	0.699825	-1.224424	-0.702958	C	0.710220	1.218425	0.702961	H	-4.761387	0.020335	-1.240016
C	0.699823	-1.224426	0.702958	C	0.710219	1.218427	-0.702960	H	-4.761387	0.020341	1.240018
C	1.295297	-2.265360	1.404306	C	1.314602	2.254213	-1.404319	H	-2.610319	0.011142	2.491957
C	-0.000248	0.000002	-1.297638	C	1.924455	3.299382	-0.697128	H	1.314920	2.254537	2.491956
C	-1.410674	0.006043	-0.702962	C	1.924458	3.299380	0.697127	H	1.314915	2.254539	-2.491956
C	-1.410674	0.006042	0.702962	H	2.363637	-4.132903	1.240022	H	2.398985	4.112484	-1.240015
C	-0.000247	0.000001	1.297638	H	2.363635	-4.132903	-1.240023	H	2.398991	4.112479	1.240016
C	-2.609882	0.011154	-1.404320	H	1.295573	-2.265698	-2.491942				
C	-3.819955	0.016312	-0.697127	H	1.295573	-2.265697	2.491942				

15

C	2.891903	-0.882875	-2.541612	C	-4.167976	2.625030	-0.113600	H	-4.162291	-3.261668	-3.277364
C	-1.407187	3.818646	-2.378332	C	-4.984612	-1.406639	0.096170	H	1.531498	-2.210144	-3.528557
C	-4.364996	-2.715998	-2.358977	C	1.882040	-2.985587	0.999509	H	0.551942	3.864299	-3.202597
C	2.041260	-1.959065	-2.601802	C	3.490112	0.544451	1.308136	H	-5.459837	-1.155823	-3.309531
C	-0.053166	3.809547	-2.300746	C	0.469021	3.371756	1.375393	H	4.172581	1.460717	-1.878549
C	-5.076792	-1.562490	-2.376685	C	-3.702323	3.131193	1.140502	H	-3.872170	2.797522	-2.252245
C	1.677884	-2.650125	-1.410222	C	-5.220845	-0.628601	1.239361	H	-2.068727	-4.011052	-2.192430
C	4.168606	0.796363	-1.012131	C	-2.221906	-3.637001	1.192998	H	-0.076429	-3.455980	-2.309801
C	0.610102	3.558963	-1.036813	C	0.610102	-3.558963	1.036813	H	2.402476	2.834469	-1.941560
C	-3.502268	3.072846	-1.268532	C	4.168605	-0.796363	1.012130	H	-5.203196	1.084307	-2.225616
C	-5.194224	-0.770580	-1.170681	C	1.677884	2.650125	1.410222	H	2.402476	-2.834470	1.941560
C	-2.452817	-3.762542	-1.207201	C	-2.452817	3.762542	1.207201	H	-0.076429	3.455980	2.309801
C	0.469021	-3.371756	-1.375393	C	-5.194224	0.770580	1.170681	H	-5.203194	-1.084306	2.225616
C	3.490112	-0.544451	-1.308136	C	-3.502268	-3.072846	1.268532	H	4.172581	-1.460717	1.878549
C	1.882040	2.985587	-0.999509	C	-0.053166	-3.809547	2.300746	H	-2.068728	4.011056	2.192430
C	-2.221906	3.637001	-1.192998	C	2.041260	1.959065	2.601802	H	-3.872171	-2.797524	2.252246
C	-5.220845	0.628600	-1.239361	C	-5.076793	1.562490	2.376685	H	0.551942	-3.864299	3.202597
C	-3.702323	-3.131193	-1.140502	C	-1.407187	-3.818646	2.378332	H	1.531498	2.210144	3.528557
C	-0.152602	-3.704434	-0.176760	C	2.891903	0.882875	2.541612	H	-5.459837	1.155823	3.309531
C	3.379533	-1.358281	-0.193878	C	-4.364996	2.715998	2.358977	H	-1.904986	-3.876246	3.343509
C	2.389478	2.391005	0.180063	C	5.576503	-0.452157	0.536743	H	3.069807	0.265114	3.418386

C	-1.614610	3.823213	0.087917	C	5.576503	0.452157	-0.536743	H	-4.162291	3.261668	3.277364
C	-4.984612	1.406639	-0.096170	C	6.774851	0.901162	-1.077721	H	6.775330	1.598605	-1.912269
C	-4.167976	-2.625030	0.113600	C	7.984672	0.447573	-0.534558	H	8.926171	0.796388	-0.950335
C	-1.614610	-3.823213	-0.087917	C	7.984672	-0.447573	0.534558	H	8.926171	-0.796388	0.950335
C	2.389478	-2.391006	-0.180063	C	6.774851	-0.901162	1.077721	H	6.775330	-1.598605	1.912269
C	3.379533	1.358281	0.193877	H	3.069807	-0.265114	-3.418386				
C	-0.152602	3.704434	0.176760	H	-1.904986	3.876246	-3.343509				

TS$_{1-15}$

C	-2.989207	1.410830	-2.456955	C	4.129500	-2.682265	-0.040587	H	3.990024	3.155366	-3.373393
C	1.588778	-4.135759	-2.381358	C	4.873382	1.415903	0.049572	H	-2.025069	3.049515	-3.415753
C	4.215711	2.649147	-2.437913	C	-1.721854	3.223904	1.167027	H	-0.329549	-4.195947	-3.300669
C	-2.291520	2.575969	-2.474002	C	-3.192814	-0.711027	1.213085	H	5.230709	1.020088	-3.358153
C	0.233088	-4.133140	-2.372330	C	-0.548376	-3.843722	1.274440	H	-3.359969	-1.204907	-2.148622
C	4.896991	1.476881	-2.429557	C	3.632417	-3.157710	1.214810	H	3.963492	-2.982021	-2.176345
C	-1.746116	3.113169	-1.249671	C	5.082938	0.668089	1.217366	H	2.009897	4.094895	-2.231247
C	-3.337392	-0.692702	-1.191058	C	2.325620	3.891572	1.159408	H	-0.106694	4.041683	-2.246744
C	-0.485618	-3.881532	-1.141202	C	-0.485522	3.881561	1.141238	H	-2.136670	-2.976839	-2.140145
C	3.563955	-3.236163	-1.198725	C	-3.337355	0.692786	1.191139	H	5.060561	-1.154373	-2.188561
C	5.045908	0.733028	-1.197064	C	-1.746159	-3.113116	1.249727	H	-2.136577	2.976901	2.140207
C	2.431607	3.880216	-1.253517	C	2.431549	-3.880245	1.253506	H	-0.106739	-4.041662	2.246773
C	-0.548320	3.843752	-1.274404	C	5.045913	-0.733108	1.197011	H	5.060619	1.154292	2.188508
C	-3.192817	0.711108	-1.213007	C	3.564038	3.236112	1.198695	H	-3.359911	1.204992	2.148703
C	-1.721936	-3.223851	-1.166972	C	0.233208	4.133154	2.372354	H	2.009851	-4.094915	2.231242
C	2.325524	-3.891599	-1.159418	C	-2.291534	-2.575907	2.474067	H	3.963586	2.981962	2.176309
C	5.082906	-0.668170	-1.217419	C	4.897001	-1.476959	2.429506	H	-0.329413	4.195972	3.300702
C	3.632460	3.157658	-1.214840	C	1.588898	4.135747	2.381360	H	-2.025078	-3.049458	3.415812
C	0.194348	4.062711	-0.107279	C	-2.989198	-1.410754	2.457031	H	5.230742	-1.020172	3.358097
C	-3.034773	1.412518	0.013573	C	4.215697	-2.649211	2.437874	H	2.138898	4.200111	3.317156
C	-2.271896	-2.660512	-0.004102	C	-5.983732	0.319984	0.546456	H	-3.297717	-0.932302	3.383239
C	1.663593	-4.074209	0.096646	C	-5.983679	-0.319926	-0.546439	H	3.990015	-3.155426	3.373357
C	4.873353	-1.415980	-0.049622	C	-7.103251	-0.727698	-1.252449	H	-7.106286	-1.275662	-2.190850
C	4.129554	2.682203	0.040549	C	-8.301260	-0.353274	-0.607404	H	-9.252074	-0.620510	-1.064071
C	1.663672	4.074195	-0.096645	C	-8.301330	0.353218	0.607208	H	-9.252199	0.620408	1.063790
C	-2.271843	2.660576	0.004165	C	-7.103397	0.727699	1.252359	H	-7.106544	1.275662	2.190760
C	-3.034802	-1.412441	-0.013497	H	-3.297748	0.932384	-3.383159				
C	0.194269	-4.062695	0.107304	H	2.138762	-4.200134	-3.317162				

TS$_{5-13}$

C	1.431859	-0.706066	-0.144724	C	-1.656118	-0.796114	-2.310451	C	-3.545897	-0.879558	1.508797
C	0.566102	-0.501103	-1.245279	C	-3.006606	-0.931174	-2.204542	C	-4.920540	-1.014683	1.579721
C	-0.834765	-0.693995	-1.142152	C	-3.653590	-0.961119	-0.924425	H	-5.410119	-1.038267	2.549540
C	0.834835	-0.693643	1.142252	C	-2.872818	-0.842701	0.265288	C	-5.686313	-1.127824	0.403943
C	-1.431791	-0.706059	0.144826	H	0.979346	-0.429783	-2.246290	H	-6.765615	-1.236813	0.465108
C	-0.566037	-0.500726	1.245321	H	-0.979284	-0.429145	2.246313	H	-2.980218	-0.807342	2.431899
C	0.263887	1.934242	-0.581764	H	-1.046864	3.066921	2.302416	C	3.545971	-0.879908	-1.508645
C	-0.263990	1.934273	0.581320	H	-0.510508	5.212641	1.121991	H	2.980290	-0.807971	-2.431768
C	-0.596533	3.062265	1.312836	H	0.509974	5.212606	-1.122716	C	5.057089	-1.103536	0.825318
C	-0.290536	4.262870	0.638703	H	1.046610	3.066855	-2.302950	H	5.636849	-1.193114	1.741114
C	0.290125	4.262849	-0.639344	H	1.178060	-0.768134	3.286653	C	4.920617	-1.015019	-1.579530
C	0.596278	3.062224	-1.313369	H	3.623447	-1.017842	3.095880	C	5.686394	-1.127802	-0.403720
C	1.656190	-0.795404	2.310579	H	-1.177987	-0.769116	-3.286532	H	6.765698	-1.236782	-0.464854
C	3.006682	-0.930464	2.204707	H	-3.623369	-1.018821	-3.095690	H	5.410196	-1.038870	-2.549343
C	3.653666	-0.960763	0.924600	C	-5.057009	-1.103898	-0.825102				
C	2.872892	-0.842708	-0.265147	H	-5.636767	-1.193755	-1.740872				

TS$_{6-14}$

C	-1.112168	-0.716823	1.234564	C	-1.233187	1.405053	2.472732	H	4.847538	-1.231092	0.000000
C	-0.945220	-1.387123	0.000000	C	-1.368865	0.709683	3.651023	H	2.702031	-2.530672	0.000000
C	-1.112168	-0.716823	-1.234564	C	-1.368863	-0.710230	3.651022	H	-1.225088	2.492435	2.470496
C	-1.112168	0.716290	1.234568	C	-1.233187	-1.405593	2.472730	H	-1.474944	1.245260	4.590478
C	-1.112168	0.716290	-1.234568	C	-1.233187	-1.405593	-2.472730	H	-1.474938	-1.245809	4.590477
C	-0.945142	1.386588	0.000000	C	-1.368863	-0.710230	-3.651022	H	-1.225090	-2.492975	2.470486
C	1.571871	-0.636624	0.000000	C	-1.368865	0.709683	-3.651023	H	-1.225090	-2.492975	-2.470486
C	1.571446	0.636926	0.000000	C	-1.233187	1.405053	-2.472732	H	-1.474938	-1.245809	-4.590477
C	2.696536	1.444709	0.000000	H	-0.899943	-2.474211	0.000000	H	-1.474944	1.245260	-4.590478
C	3.896598	0.703236	0.000000	H	-0.899905	2.473679	0.000000	H	-1.225088	2.492435	-2.470496
C	3.897118	-0.701159	0.000000	H	2.700128	2.531859	0.000000				
C	2.697609	-1.443524	0.000000	H	4.846621	1.233880	0.000000				

16a

C	-2.087348	-0.702041	-2.358883	C	-2.036304	-2.445061	1.329050	H	2.112939	4.189417	-3.173823
C	3.840287	-3.206366	-2.417210	C	2.005078	-3.482764	1.379409	H	-1.730159	1.249604	-3.227529
C	2.636441	3.816464	-2.296777	C	5.586927	-1.388873	1.008728	H	2.112922	-4.189415	-3.173817
C	-2.087344	0.702048	-2.358881	C	4.807404	2.548368	1.128421	H	4.301352	3.073091	-3.393043
C	2.636427	-3.816470	-2.296769	C	0.598962	3.386238	1.422659	H	-2.046302	-3.408243	-1.914172
C	3.840297	3.206354	-2.417217	C	-2.036292	2.445069	1.329052	H	5.470586	-1.091857	-2.388236
C	-2.503385	1.380642	-1.226479	C	-3.334633	-1.672451	1.058448	H	0.014153	3.991373	-1.906902
C	-2.416856	-2.895961	-1.024856	C	0.598945	-3.386241	1.422663	H	-2.046293	3.408249	-1.914170
C	1.945368	-3.822351	-1.022469	C	4.807390	-2.548374	1.128429	H	0.014140	-3.991373	-1.906898
C	5.309073	-1.488330	-1.389615	C	5.586938	1.388862	1.008722	H	5.470586	1.091832	-2.388240
C	4.482105	2.609827	-1.261813	C	2.005095	3.482758	1.379402	H	-3.714152	1.172621	1.952583
C	0.554086	3.855217	-0.974179	C	-1.338423	2.450144	2.558409	H	2.540844	-3.278254	2.301237
C	-2.416846	2.895968	-1.024853	C	-0.056229	-2.944095	2.608716	H	4.623194	2.931275	2.128476
C	-2.503393	-1.380634	-1.226482	C	6.014100	0.677810	2.195794	H	-3.714163	-1.172610	1.952578
C	0.554072	-3.855218	-0.974175	C	-0.056212	2.944096	2.608713	H	4.623178	-2.931279	2.128484
C	4.482097	-2.609843	-1.261805	C	-1.338437	-2.450137	2.558409	H	2.540863	3.278245	2.301229
C	5.309076	1.488311	-1.389620	C	6.014095	-0.677819	2.195796	H	-1.799362	2.021325	3.444947
C	1.945382	3.822347	-1.022476	C	-4.324466	-2.682758	0.485963	H	0.511742	-2.905712	3.535028
C	-1.507344	3.052490	0.203377	C	-3.815449	-3.361742	-0.632387	H	6.189215	1.238647	3.110696
C	-2.976429	-0.695073	-0.082282	C	-4.580761	-4.328017	-1.274170	H	0.511760	2.905710	3.535023
C	-0.153770	-3.505590	0.198345	C	-5.862722	-4.621955	-0.790828	H	-1.799375	-2.021313	3.444944
C	4.060210	-3.019434	0.042905	C	-6.366895	-3.948682	0.321496	H	6.189204	-1.238654	3.110701
C	5.670931	0.732734	-0.259638	C	-5.595793	-2.972607	0.966476	H	-4.188648	-4.852537	-2.142564
C	2.701041	3.560723	0.175844	C	-4.324455	2.682768	0.485969	H	-6.465395	-5.378195	-1.286692
C	-0.153755	3.505590	0.198342	C	-3.815439	3.361750	-0.632382	H	-7.362189	-4.180438	0.691230
C	-2.976425	0.695082	-0.082280	C	-4.580752	4.328025	-1.274164	H	-5.989289	-2.447397	1.833852
C	-1.507357	-3.052486	0.203377	C	-5.862712	4.621964	-0.790819	H	-4.188639	4.852544	-2.142558
C	2.701027	-3.560728	0.175851	C	-6.366883	3.948693	0.321506	H	-6.465386	5.378204	-1.286683
C	5.670927	-0.732749	-0.259636	C	-5.595782	2.972617	0.966485	H	-7.362177	4.180448	0.691241
C	4.060224	3.019425	0.042898	H	-1.730167	-1.249597	-3.227532	H	-5.989275	2.447409	1.833862
C	-3.334623	1.672460	1.058452	H	4.301342	-3.073104	-3.393036				

16b

C	-2.577797	-1.704323	2.446313	C	-4.053168	-0.805279	-1.361873	H	3.642951	-0.826262	3.492325
C	-1.706339	4.600606	2.280311	C	-3.024787	3.182465	-1.476897	H	-0.727322	-2.223432	3.386605
C	3.793871	-0.204432	2.613255	C	0.842140	4.738869	-1.123860	H	-3.474145	3.720066	3.070583
C	-1.305228	-2.216257	2.465803	C	4.038199	2.151025	-0.910060	H	3.397349	1.641769	3.623998
C	-2.875358	3.921479	2.185538	C	3.313139	-1.698424	-1.233319	H	-4.964551	-0.399926	1.876109
C	3.662504	1.159783	2.686342	C	0.659254	-2.594598	-1.247648	H	0.945812	4.768493	2.284391
C	-0.659249	-2.594590	1.247655	C	-3.849876	-2.290019	-1.062055	H	3.522880	-2.854192	1.937902
C	-4.635154	-0.955902	0.996158	C	-3.690063	1.941786	-1.495834	H	1.288369	-2.688534	2.129121
C	-3.263328	3.310207	0.929890	C	-0.550083	4.778450	-1.272915	H	-4.418930	1.772673	1.855062
C	0.550071	4.778440	1.272919	C	3.263311	3.310207	-0.929894	H	2.607066	3.528573	2.417058
C	3.690066	1.941790	1.495828	C	4.635152	-0.955900	-0.996162	H	-1.288365	-2.688549	-2.129114
C	3.849882	-2.290016	1.062056	C	1.305232	-2.216279	-2.465799	H	-2.607053	3.528565	-2.417055
C	0.737098	-2.725216	1.193362	C	-3.662493	1.159776	-2.686347	H	4.418906	1.772672	-1.855073
C	-3.313135	-1.698413	1.233316	C	2.875340	3.921485	-2.185540	H	-3.522870	-2.854193	-1.937901
C	-4.038217	2.151025	0.910051	C	2.577802	-1.704346	-2.446315	H	-0.945826	4.768510	-2.284387
C	-0.842153	4.738859	1.123865	C	-3.793861	-0.204438	-2.613259	H	4.964546	-0.399925	-1.876116
C	3.024789	3.182471	1.476894	C	1.706326	4.600619	-2.280309	H	0.727328	-2.223462	-3.386602
C	4.053170	-0.805276	1.361870	C	-5.183562	-2.789444	-0.513373	H	-3.397331	1.641761	-3.624001
C	1.433526	-2.636753	-0.021176	C	-5.633229	-2.033162	0.580447	H	3.474126	3.720070	-3.070585
C	-2.827955	-2.307487	0.096151	C	-6.847759	-2.328085	1.187015	H	3.022051	-1.291189	-3.348588
C	-4.128272	1.349947	-0.252534	C	-7.623453	-3.384583	0.690025	H	-3.642939	-0.826270	-3.492327
C	-1.398819	4.555422	-0.178242	C	-7.178594	-4.133117	-0.398911	H	1.344873	4.952895	-3.243573
C	2.644990	3.784568	0.282508	C	-5.951994	-3.835368	-1.008621	H	-7.194947	-1.745039	2.036981
C	4.365481	-0.059758	0.238329	C	5.633235	-2.033151	-0.580447	H	-8.575904	-3.619781	1.157485
C	2.827959	-2.307488	-0.096148	C	5.183569	-2.789437	0.513372	H	-7.784122	-4.951788	-0.778494
C	-1.433522	-2.636753	0.021182	C	5.952006	-3.835355	1.008622	H	-5.605616	-4.418940	-1.858520
C	-4.365489	-0.059760	-0.238336	C	7.178611	-4.133096	0.398916	H	5.605630	-4.418930	1.858520
C	-2.645001	3.784567	-0.282508	C	7.623468	-3.384559	-0.690018	H	7.784143	-4.951761	0.778503
C	1.398807	4.555423	0.178244	C	6.847768	-2.328066	-1.187011	H	8.575922	-3.619750	-1.157475
C	4.128262	1.349948	0.252526	H	-3.022048	-1.291157	3.348581	H	7.194954	-1.745020	-2.036978
C	-0.737095	-2.725223	-1.193353	H	-1.344884	4.952871	3.243578				

16c

C	-0.678812	-3.429059	2.305614	C	-2.709383	-2.643291	-1.357474	H	4.235685	0.413526	3.435719
C	-3.154585	2.054305	2.575447	C	-5.215653	0.765647	-1.035220	H	1.224947	-3.456799	3.245765

C	4.011796	0.983459	2.537367	C	-1.434807	3.270798	-1.076894	H	-4.235685	0.413528	3.435719
C	0.678812	-3.429060	2.305614	C	2.749734	2.797170	-1.078626	H	2.683811	2.356818	3.507373
C	-4.011796	0.983460	2.537367	C	4.558135	-0.589709	-1.297747	H	-3.229319	-2.615207	2.037142
C	3.154585	2.054303	2.575448	C	2.709383	-2.643289	-1.357474	H	-0.976747	3.428812	2.304148
C	1.434808	-3.270799	1.076896	C	-1.456462	-3.282057	-1.342609	H	5.216441	-1.406704	1.919475
C	-2.749733	-2.797169	1.078629	C	-4.558135	-0.589709	-1.297747	H	3.229320	-2.615207	2.037141
C	-4.558135	0.589707	1.297746	C	-2.749733	2.797169	-1.078627	H	-5.216441	-1.406703	1.919475
C	-1.456463	3.282055	1.342612	C	1.434807	3.270799	-1.076894	H	0.976745	3.428809	2.304148
C	2.709383	2.643288	1.357476	C	5.215653	0.765647	-1.035220	H	0.976744	-3.428810	-2.304146
C	5.215655	-0.765647	1.035218	C	3.154583	-2.054305	-2.575446	H	-5.216438	1.406702	-1.919477
C	2.749733	-2.797169	1.078628	C	-4.011796	-0.983463	-2.537367	H	3.229319	2.615208	-2.037140
C	-1.434807	-3.270799	1.076897	C	0.678812	3.429057	-2.305611	H	-0.976746	-3.428814	-2.304146
C	-5.215654	-0.765647	1.035218	C	4.011796	-0.983461	-2.537367	H	-3.229319	2.615208	-2.037141
C	-2.709384	2.643290	1.357475	C	-3.154584	-2.054308	-2.575446	H	5.216439	1.406703	-1.919477
C	1.456463	3.282054	1.342612	C	-0.678812	3.429057	-2.305611	H	2.683809	-2.356819	-3.507371
C	4.558135	0.589707	1.297746	C	-6.623869	0.441254	-0.546120	H	-4.235684	-0.413532	-3.435720
C	3.366295	-2.330596	-0.110468	C	-6.623870	-0.441252	0.546116	H	1.224946	3.456794	-3.245763
C	-0.735868	-3.445696	-0.168087	C	-7.822275	-0.877926	1.096884	H	4.235683	-0.413530	-3.435720
C	-4.410951	-1.352276	-0.150390	C	-9.032060	-0.436100	0.543954	H	-2.683810	-2.356822	-3.507371
C	-3.366296	2.330597	0.110468	C	-9.032059	0.436108	-0.543958	H	-1.224945	3.456793	-3.245763
C	0.735867	3.445694	0.168090	C	-7.822273	0.877931	-1.096888	H	-7.822762	-1.557233	1.946249
C	4.410952	1.352275	0.150390	C	6.623869	0.441254	-0.546119	H	-9.973592	-0.776112	0.966893
C	4.410952	-1.352276	-0.150390	C	6.623870	-0.441253	0.546116	H	-9.973590	0.776123	-0.966896
C	0.735868	-3.445696	-0.168088	C	7.822276	-0.877927	1.096883	H	-7.822758	1.557237	-1.946253
C	-3.366295	-2.330596	-0.110467	C	9.032060	-0.436100	0.543954	H	7.822762	-1.557234	1.946248
C	-4.410952	1.352275	0.150389	C	9.032059	0.436108	-0.543958	H	9.973593	-0.776113	0.966892
C	-0.735867	3.445694	0.168090	C	7.822273	0.877931	-1.096888	H	9.973591	0.776123	-0.966896
C	3.366296	2.330596	0.110469	H	-1.224946	-3.456799	3.245766	H	7.822758	1.557237	-1.946253
C	1.456462	-3.282055	-1.342610	H	-2.683812	2.356820	3.507372				

TS$_{15\text{-}16a}$

C	-2.482202	-0.714550	-2.441982	C	-2.372342	-2.458422	1.315353	H	2.953216	4.259293	-3.080383
C	3.340965	-3.350524	-2.554692	C	1.530956	-3.841630	1.226330	H	-1.818889	1.110305	-3.351458
C	3.387135	3.799639	-2.195686	C	5.259991	-1.961959	0.982191	H	1.540430	-4.133689	-3.369879
C	-2.161646	0.623704	-2.441889	C	5.131197	2.077545	1.273533	H	4.990388	2.866025	-3.241149
C	2.096080	-3.881755	-2.469745	C	1.165428	3.610376	1.424321	H	-2.690386	-3.407657	-1.918469
C	4.504097	3.037208	-2.283795	C	-1.414975	2.452682	1.280018	H	5.197096	-1.436904	-2.389849
C	-2.109599	1.340572	-1.217495	C	-3.545340	-1.498251	1.094789	H	0.773090	4.062379	-1.935003
C	-2.938533	-2.845270	-1.016209	C	0.136278	-3.660595	1.302175	H	-0.946529	2.915295	-2.067240
C	1.418274	-3.967089	-1.191609	C	4.366753	-3.041342	1.023589	H	-0.535101	-3.971584	-2.051343
C	4.991715	-1.880070	-1.419421	C	5.675379	0.794347	1.128404	H	5.704500	0.669615	-2.279983
C	4.971808	2.306762	-1.124349	C	2.567691	3.598493	1.453731	H	-2.821422	1.080843	2.126471
C	1.253690	3.939328	-0.968810	C	-0.856090	2.842666	2.554048	H	2.087515	-3.743497	2.152846
C	-1.352012	2.532521	-1.136316	C	-0.445322	-3.227706	2.528447	H	4.960645	2.440184	2.283509
C	-2.867528	-1.331072	-1.235179	C	5.923818	-0.032172	2.290630	H	-3.801363	-0.940336	1.997765
C	0.024866	-3.944367	-1.120346	C	0.390718	3.376111	2.623608	H	4.138551	-3.466568	1.996790
C	4.044734	-2.911115	-1.365782	C	-1.658871	-2.584928	2.527421	H	3.046454	3.436992	2.415406
C	5.565872	1.046575	-1.270374	C	5.734972	-1.372467	2.216378	H	-1.401086	2.590976	3.460594
C	2.650853	3.853958	-0.949792	C	-4.700653	-2.360962	0.597023	H	0.143874	-3.295315	3.439541
C	-0.815720	2.960564	0.082924	C	-4.355220	-3.128060	-0.526486	H	6.138056	0.450881	3.240922
C	-3.081834	-0.600447	-0.075781	C	-5.278538	-3.996289	-1.095711	H	0.863445	3.550674	3.587214
C	-0.648061	-3.651129	0.089304	C	-6.556709	-4.104102	-0.531013	H	-2.049863	-2.134695	3.436529
C	3.592642	-3.379057	-0.092495	C	-6.898535	-3.344885	0.587510	H	5.794899	-1.995488	3.105489
C	5.703911	0.187830	-0.169208	C	-5.966705	-2.467882	1.159120	H	-5.012774	-4.587663	-1.968945
C	3.319990	3.518709	0.270611	C	-4.266516	3.512953	0.363604	H	-7.283575	-4.782986	-0.968893
C	0.491676	3.616287	0.162243	C	-3.762319	3.998220	-0.687255	H	-7.891844	-3.431964	1.019603
C	-2.619116	0.746716	-0.009547	C	-4.294317	5.019367	-1.456632	H	-6.233585	-1.876142	2.031744
C	-1.966768	-3.101461	0.158530	C	-5.513260	5.492553	-0.925028	H	-3.862494	5.437017	-2.361870
C	2.204858	-3.850963	0.009838	C	-6.074523	4.953561	0.245550	H	-6.033480	6.299285	-1.437458
C	5.450579	-1.254752	-0.246332	C	-5.452677	3.907042	0.960030	H	-7.017175	5.354524	0.612565
C	4.561605	2.742044	0.177618	H	-2.394222	-1.305749	-3.350086	H	-5.889549	3.490947	1.863635
C	-2.410405	1.466949	1.197633	H	3.797980	-3.165033	-3.523920				

TS$_{15\text{-}16b}$

C	-2.952804	-1.606387	2.535605	C	-4.096052	-0.667282	-1.343338	H	4.265405	-0.597014	3.443363
C	-1.288340	4.532019	2.311214	C	-2.685530	3.219613	-1.449849	H	-1.154661	-2.164814	3.555265
C	4.252128	-0.031227	2.515118	C	1.159050	4.847837	-1.156980	H	-3.078463	3.710694	3.111903
C	-1.709251	-2.181499	2.620419	C	4.207796	2.163543	-1.120690	H	4.439561	1.871567	3.455505
C	-2.491855	3.913780	2.219093	C	2.923988	-1.841835	-1.085602	H	-5.155456	-0.114136	1.824314
C	4.351211	1.323112	2.520897	C	0.339966	-2.997809	-0.983627	H	1.373881	4.708739	2.246168

C	-1.055950	-2.650898	1.443833	C	-4.089375	-2.164725	-1.023184	H	2.957996	-2.340323	2.276211
C	-4.839160	-0.716724	0.970555	C	-3.438319	2.029870	-1.478437	H	0.861375	-2.588941	2.371961
C	-2.941650	3.364616	0.955667	C	-0.238478	4.860500	-1.259097	H	-4.229737	1.923728	1.861518
C	0.945445	4.766964	1.249543	C	3.547856	3.397342	-1.094321	H	3.298131	3.739131	2.291217
C	4.165434	2.063192	1.295168	C	3.907491	-0.830223	-1.122665	H	-1.575324	-3.147256	-1.915066
C	3.192902	-1.913031	1.305526	C	1.059948	-2.909889	-2.235142	H	-2.221164	3.526231	-2.381572
C	0.346933	-2.762551	1.431989	C	-3.431822	1.231096	-2.658067	H	4.430746	1.732187	-2.092735
C	-3.625461	-1.594005	1.293190	C	3.120612	4.028901	-2.325376	H	-3.779949	-2.771626	-1.876412
C	-3.815207	2.276816	0.920984	C	2.286314	-2.328294	-2.287306	H	-0.667820	4.889409	-2.256456
C	-0.450224	4.705028	1.141028	C	-3.702017	-0.112877	-2.580825	H	4.187473	-0.442627	-2.098067
C	3.557784	3.327260	1.319963	C	1.969005	4.742899	-2.353092	H	0.549501	-3.202213	-3.149618
C	3.942916	-0.717548	1.285515	C	-5.503525	-2.495676	-0.557001	H	-3.078494	1.671969	-3.586816
C	1.070582	-2.799412	0.243104	C	-5.928419	-1.674949	0.499363	H	3.669961	3.818108	-3.239747
C	-3.143788	-2.283852	0.195034	C	-7.204841	-1.815913	1.029623	H	2.772825	-2.147056	-3.242672
C	-3.974363	1.495594	-0.247953	C	-8.067210	-2.781579	0.492753	H	-3.568470	-0.757488	-3.446082
C	-1.045446	4.572288	-0.151828	C	-7.646532	-3.594110	-0.559456	H	1.569084	5.121748	-3.290558
C	3.039898	3.897849	0.150322	C	-6.357806	-3.451953	-1.091806	H	-7.533046	-1.183008	1.850966
C	4.178508	-0.070481	0.043639	C	5.770084	-2.714709	-0.610649	H	-9.068141	-2.896048	0.900170
C	2.437597	-2.281134	0.172798	C	5.463625	-3.240427	0.501106	H	-8.319622	-4.341806	-0.970369
C	-1.791192	-2.752303	0.203341	C	6.160894	-4.254979	1.136578	H	-6.030460	-4.085288	-1.913155
C	-4.382851	0.124617	-0.244874	C	7.287014	-4.673067	0.396142	H	5.904337	-4.708639	2.090235
C	-2.300409	3.814759	-0.253456	C	7.621449	-4.091170	-0.838216	H	7.913441	-5.469639	0.792528
C	1.768454	4.630115	0.118187	C	6.849166	-3.056635	-1.408030	H	8.501342	-4.447116	-1.370274
C	4.320951	1.382872	0.042137	H	-3.402524	-1.124740	3.400343	H	7.111104	-2.606564	-2.361852
C	-1.055610	-3.054337	-0.965168	H	-0.891915	4.828904	3.279400				

TS$_{15-16c}$

C	0.484888	3.795217	2.327978	C	2.750429	3.071956	-1.205052	H	-4.044954	-0.240540	3.423945
C	3.382700	-2.622967	2.423438	C	4.412816	-0.666100	-1.206808	H	-1.467762	3.849059	3.165964
C	-3.861697	-0.863319	2.551842	C	1.292520	-3.605951	-1.138741	H	4.470631	-1.040698	3.350601
C	-0.869667	3.788308	2.259813	C	-2.821931	-2.974711	-0.974911	H	-2.504365	-2.183440	3.552767
C	4.109972	-1.476312	2.422128	C	-4.454463	0.533834	-1.313507	H	2.958434	2.791689	2.193715
C	-3.009825	-1.937803	2.622213	C	-2.637176	2.631658	-1.435221	H	1.102860	-3.931262	2.251080
C	-1.544431	3.536689	1.001832	C	1.499050	3.702591	-1.266063	H	-5.134400	1.477163	1.865720
C	2.576772	3.052376	1.210375	C	4.277914	0.738122	-1.195341	H	-3.337135	2.830259	1.920888
C	4.277916	-0.738184	1.195261	C	2.576724	-3.052422	-1.210435	H	4.439112	1.158475	2.174905
C	1.499024	-3.702626	1.266015	C	-1.544481	-3.536693	-1.001844	H	-0.885101	-3.423472	2.349501
C	-2.637188	-2.631651	1.435222	C	-5.130755	-0.804996	-1.005235	H	-0.885090	3.423460	-2.349521
C	-5.130734	0.805031	1.005265	C	-3.009834	1.937814	-2.622208	H	4.439073	-1.158539	-2.174987
C	-2.821887	2.974720	0.974913	C	4.109963	1.476253	-2.422206	H	-3.337188	-2.830244	-1.920879
C	1.292572	3.605918	1.138696	C	-0.869735	-3.788319	-2.259833	H	1.102877	3.931230	-2.251123
C	4.412846	0.666038	1.206725	C	-3.861717	0.863339	-2.551827	H	2.958377	-2.791739	-2.193780
C	2.750409	-3.072003	1.204990	C	3.382703	2.622915	-2.423508	H	-5.134439	-1.477128	-1.865690
C	-1.422962	-3.344264	1.410402	C	0.484820	-3.795242	-2.328014	H	-2.504382	2.183445	-3.552768
C	-4.454453	-0.533807	1.313528	C	7.043389	-0.309506	-0.552823	H	4.470609	1.040636	-3.350684
C	-3.342771	2.381522	-0.199932	C	7.043315	0.309509	0.552837	H	-1.467841	-3.849064	-3.165977
C	0.674501	3.781552	-0.140066	C	8.163503	0.701673	1.266783	H	-4.044991	0.240562	-3.423927
C	4.080892	1.403235	0.046717	C	9.361671	0.340846	0.614517	H	3.142932	3.129253	-3.355285
C	3.239180	-2.597358	-0.054176	C	9.361764	-0.340700	-0.614219	H	0.988703	-3.857644	-3.289731
C	-0.789542	-3.672866	0.217304	C	8.163696	-0.701598	-1.266628	H	8.166613	1.230878	2.215944
C	-4.339689	-1.355212	0.205070	C	-6.538633	-0.457300	-0.532351	H	10.312424	0.599187	1.076414
C	-4.339676	1.355238	-0.205049	C	-6.538622	0.457350	0.532397	H	10.312588	-0.598984	-1.076003
C	-0.789504	3.672853	-0.217325	C	-7.736970	0.911424	1.069146	H	8.166954	-1.230803	-2.215789
C	3.239210	2.597306	0.054108	C	-8.946769	0.452687	0.530314	H	-7.737440	1.616776	1.897014
C	4.080874	-1.403295	-0.046795	C	-8.946781	-0.452610	-0.530239	H	-9.888269	0.805465	0.942721
C	0.674461	-3.781579	0.140028	C	-7.736993	-0.911360	-1.069085	H	-9.888289	-0.805378	-0.942635
C	-3.342795	-2.381508	0.199941	H	0.988783	3.857613	3.289690	H	-7.737481	-1.616712	-1.896954
C	-1.422941	3.344258	-1.410416	H	3.142934	-3.129301	3.355218				

17

C	3.291210	-1.585688	-0.694324	C	-2.247553	-0.971949	-1.403034	H	-2.245992	-0.977295	-2.490802
C	3.290686	-1.584942	0.699268	C	-1.218907	-0.358077	-0.701003	H	-2.244979	-0.973882	2.494532
C	2.246674	-0.970405	1.406765	C	-1.218545	-0.357083	0.703619	H	-4.101390	-2.063019	1.242861
C	1.218550	-0.357074	0.703621	C	-2.246665	-0.970423	1.406762	H	-4.102205	-2.064432	-1.236888
C	1.218914	-0.358069	-0.701001	C	-3.290672	-1.584966	0.699263	H	0.000346	2.715919	2.623227
C	2.247563	-0.971936	-1.403031	C	-3.291196	-1.585707	-0.694329	H	-0.882752	3.631464	1.400664
C	-0.000001	0.365082	1.282373	H	4.102222	-2.064408	-1.236882	H	0.882292	3.631884	1.400143
C	-0.000003	1.784142	0.672768	H	4.101408	-2.062988	1.242867	H	-0.000004	3.910839	-1.032034
C	-0.000002	1.777931	-0.669123	H	2.244987	-0.973862	2.494536	H	-0.881340	2.959822	-2.240408
C	0.000002	0.361731	-1.280283	H	2.246003	-0.977284	-2.490798	H	0.881307	2.959820	-2.240429

C	-0.000029	3.000657	1.566337	H	-0.000003	0.377217	2.375381
C	-0.000009	2.965763	-1.583354	H	0.000003	0.376285	-2.373731

18a

C	-2.800651	-2.450193	-0.370035	C	-9.377944	-1.385577	-0.021143	H	7.774549	-3.157627	-0.757928
C	-3.570084	-1.255990	-0.137182	C	-10.094104	-0.155810	0.220989	H	5.317211	-3.202034	-0.705224
C	-2.895886	-0.010658	0.069528	C	-9.337999	1.018157	0.413598	H	7.464689	2.310715	0.235890
C	-1.433903	0.020924	0.039133	C	-10.105756	-2.590876	-0.225782	H	9.847104	-1.960483	-0.595328
C	-0.715771	-1.196774	-0.196684	C	-11.482033	-2.601762	-0.196033	H	9.552434	3.508824	0.408841
C	-1.443880	-2.422140	-0.398273	C	-12.184251	-1.400965	0.048176	H	12.030030	3.526942	0.357590
C	-0.684771	1.185560	0.229218	C	-11.516218	-0.208433	0.251694	H	-5.438170	-2.270461	-0.273189
C	0.715764	1.196775	0.196671	C	-13.704254	-1.250264	0.110395	H	-3.219990	2.088296	0.454106
C	1.433897	-0.020923	-0.039144	C	-14.063281	-0.209132	-0.950822	H	-7.774556	3.157618	0.757960
C	0.684764	-1.185559	-0.229229	C	-13.388355	1.004510	-0.744362	H	-5.317218	3.202028	0.705248
C	1.443873	2.422142	0.398258	C	-12.481703	0.958146	0.487436	H	-7.464696	-2.310707	-0.235947
C	2.800645	2.450195	0.370017	C	-14.934806	-0.356850	-2.020843	H	-9.847111	1.960476	0.595347
C	3.570077	1.255991	0.137165	C	-15.137881	0.724045	-2.892594	H	-9.552441	-3.508813	-0.408919
C	2.895880	0.010658	-0.069539	C	-14.468230	1.928636	-2.687952	H	-12.030037	-3.526931	-0.357669
C	4.971520	1.303581	0.111374	C	-13.584037	2.073527	-1.607823	H	-14.225682	-2.199778	-0.038445
C	5.760574	0.173339	-0.110463	C	-14.030143	-0.605216	1.476438	H	-11.980003	1.911146	0.664010
C	5.085383	-1.072480	-0.317560	C	-13.385122	0.553444	1.675323	H	-15.455211	-1.298075	-2.183477
C	3.687086	-1.121645	-0.292118	C	13.704244	1.250269	-0.110456	H	-15.820323	0.618032	-3.731774
C	7.227524	0.205943	-0.140251	C	14.063295	0.209178	0.950799	H	-14.628675	2.761314	-3.367701
C	7.947121	-1.021444	-0.379029	C	13.388339	-1.004461	0.744425	H	-13.059241	3.013343	-1.451327
C	7.209978	-2.245335	-0.580526	C	12.481698	-0.958161	-0.487385	H	14.225670	2.199791	0.038339
C	5.855602	-2.269872	-0.551412	C	14.934839	0.356942	2.020798	H	11.979999	-1.911172	-0.663909
C	7.967765	1.366225	0.051707	C	15.137905	-0.723906	2.892610	H	15.455259	1.298169	2.183373
C	9.377937	1.385581	0.021100	C	14.468228	-1.928495	2.688050	H	15.820361	-0.617856	3.731775
C	10.094097	0.155810	-0.221013	C	13.584011	-2.073431	1.607946	H	14.628671	-2.761138	3.367844
C	9.337992	-1.018161	-0.413597	C	13.385130	-0.553527	-1.675283	H	13.059202	-3.013249	1.451508
C	10.105749	2.590883	0.225723	C	14.030095	0.605178	-1.476483	H	15.970050	1.437670	-1.895238
C	11.482026	2.601768	0.195978	C	14.994097	1.308080	-2.383935	H	14.633506	2.314738	-2.638222
C	12.184243	1.400966	-0.048207	C	13.449029	-1.467613	-2.861859	H	15.159436	0.767062	-3.319952
C	11.516211	0.208432	-0.251712	C	-13.448917	1.467391	2.862013	H	13.798564	-2.467742	-2.569000
C	-4.971526	-1.303580	-0.111393	C	-14.994102	-1.308192	2.383883	H	14.119771	-1.095718	-3.641589
C	-5.760581	-0.173338	0.110447	H	-3.336367	-3.383782	-0.524254	H	12.454848	-1.600278	-3.311122
C	-5.085390	1.072479	0.317557	H	-0.876700	-3.332790	-0.575360	H	-13.797631	2.467800	2.569149
C	-3.687093	1.121644	0.292115	H	-1.185725	2.131503	0.410961	H	-12.454847	1.599305	3.311752
C	-7.227531	-0.205942	0.140231	H	1.185719	-2.131502	-0.410973	H	-14.120257	1.095829	3.641388
C	-7.947127	1.021441	0.379030	H	0.876694	3.332791	0.575345	H	-15.969778	-1.438610	1.894868
C	-7.209984	2.245330	0.580543	H	3.336360	3.383784	0.524234	H	-15.160119	-0.766771	3.319549
C	-5.855609	2.269867	0.551425	H	5.438164	2.270463	0.273167	H	-14.633028	-2.314504	2.638868
C	-7.967772	-1.366221	-0.051749	H	3.219983	-2.088299	-0.454100				

18b

C	-2.787721	2.492604	-0.000342	C	-9.372010	1.418357	-0.000336	H	0.858303	-3.386912	-0.000166
C	-3.564070	1.280336	-0.000224	C	-10.095507	0.169830	-0.000041	H	3.318354	-3.441695	-0.000221
C	-2.896630	0.014178	-0.000084	C	-9.346277	-1.023540	0.000194	H	5.427177	-2.312862	-0.000154
C	-1.434317	-0.019565	-0.000119	C	-10.093051	2.645265	-0.000608	H	3.232038	2.117904	0.000121
C	-0.709207	1.216796	-0.000197	C	-11.468551	2.658933	-0.000587	H	7.794349	3.199120	0.000336
C	-1.430735	2.462440	-0.000311	C	-12.180421	1.438069	-0.000291	H	5.336621	3.247064	0.000248
C	-0.691640	-1.203625	-0.000109	C	-11.519134	0.223185	-0.000023	H	7.453575	-2.356961	-0.000429
C	0.709207	-1.216798	-0.000117	C	-13.705568	1.305402	-0.000220	H	9.859985	1.980641	0.000258
C	1.434317	0.019564	-0.000120	C	-14.052630	0.450697	1.221031	H	9.533879	-3.577781	-0.000588
C	0.691640	1.203624	-0.000188	C	-13.384901	-0.785177	1.222101	H	12.011277	-3.600956	-0.000552
C	1.430735	-2.462441	-0.000149	C	-12.480944	-0.972679	0.000274	H	-5.427178	2.312861	-0.000310
C	2.787721	-2.492605	-0.000176	C	-14.918726	0.782467	2.254947	H	-3.232037	-2.117905	0.000264
C	3.564070	-1.280337	-0.000138	C	-15.118461	-0.128528	3.301557	H	-7.794350	-3.199121	0.000553
C	2.896629	-0.014179	-0.000084	C	-14.456084	-1.355188	3.302809	H	-5.336621	-3.247066	0.000468
C	4.965574	-1.330113	-0.000128	C	-13.583988	-1.689561	2.257436	H	-7.453574	2.356960	-0.000588
C	5.760829	-0.182618	-0.000062	C	-14.052698	0.450131	-1.221052	H	-9.859985	-1.980641	0.000392
C	5.092562	1.083998	0.000036	C	-13.384967	-0.785742	-1.221589	H	-9.533877	3.577780	-0.000831
C	3.694115	1.135370	0.000022	C	13.705569	-1.305401	-0.000121	H	-12.011276	3.600957	-0.000797
C	7.227955	-0.217376	-0.000072	C	14.052709	-0.450218	-1.221012	H	-14.214348	2.272966	-0.000430
C	7.954706	1.028634	0.000100	C	13.384967	0.785650	-1.221650	H	-11.980150	-1.941964	0.000488
C	7.224504	2.273012	0.000221	C	12.480944	0.972680	0.000198	H	-15.436299	1.738993	2.253734
C	5.869829	2.299374	0.000175	C	14.918872	-0.781582	-2.255002	H	-15.793177	0.124866	4.114906
C	7.961958	-1.397495	-0.000254	C	15.118666	0.129816	-3.301250	H	-14.614849	-2.057015	4.117173
C	9.372010	-1.418358	-0.000239	C	14.456279	1.356471	-3.302070	H	-13.068336	-2.647117	2.258661
C	10.095508	-0.169831	-0.000027	C	13.584112	1.690433	-2.256625	H	14.214349	-2.272965	-0.000257

C	9.346277	1.023539	0.000125	C	13.384899	0.785272	1.222040	H	11.980149	1.941964	0.000336
C	10.093052	-2.645265	-0.000430	C	14.052620	-0.450607	1.221071	H	15.436450	-1.738106	-2.254127
C	11.468552	-2.658933	-0.000409	C	-14.918853	0.781420	-2.255074	H	15.793435	-0.123260	-4.114653
C	12.180422	-1.438069	-0.000195	C	-15.118647	-0.130058	-3.301250	H	14.615091	2.058614	-4.116153
C	11.519134	-0.223184	-0.000007	C	-14.456267	-1.356717	-3.301973	H	13.068456	2.647987	-2.257511
C	-4.965575	1.330112	-0.000218	C	-13.584112	-1.690605	-2.256494	H	-15.436427	1.737947	-2.254274
C	-5.760829	0.182616	-0.000075	C	14.918707	-0.782301	2.255018	H	-15.793410	0.122958	-4.114678
C	-5.092563	-1.083999	0.000109	C	15.118443	0.128774	3.301558	H	-14.615078	-2.058921	-4.116003
C	-3.694115	-1.135371	0.000099	C	14.456072	1.355438	3.302711	H	-13.068459	-2.648161	-2.257305
C	-7.227955	0.217375	-0.000087	C	13.583984	1.689734	2.257307	H	15.436278	-1.738829	2.253881
C	-7.954706	-1.028635	0.000169	H	-3.318354	3.441695	-0.000450	H	15.793154	-0.124560	4.114930
C	-7.224504	-2.273013	0.000375	H	-0.858303	3.386911	-0.000389	H	14.614835	2.057326	4.117023
C	-5.869829	-2.299375	0.000331	H	-1.198177	-2.163974	-0.000120	H	13.068335	2.647292	2.258456
C	-7.961957	1.397494	-0.000350	H	1.198176	2.163973	-0.000261				

19a

C	11.625589	1.414533	-2.274293	C	-9.001958	-0.609144	2.502064	H	1.458730	-1.742334	1.660953
C	12.350928	0.871783	-1.190855	C	-9.964093	0.390288	2.746013	H	-0.990427	0.151199	-2.194880
C	11.709711	0.227999	-0.149586	C	-10.701418	0.900675	1.690454	H	-0.566579	-2.495293	2.677207
C	10.292816	0.092731	-0.138961	C	-10.498625	0.439950	0.370415	H	-3.014357	-2.723225	2.665616
C	9.553809	0.641447	-1.250923	C	-11.784302	1.977744	1.765100	H	-5.142798	-2.354611	1.622186
C	10.254248	1.296486	-2.301894	C	-11.328644	3.096513	0.829393	H	-3.009092	-0.033408	-2.207790
C	9.562822	-0.543807	0.885280	C	-11.126794	2.640391	-0.483544	H	-7.619750	0.101848	-3.236697
C	8.176533	-0.656208	0.851478	C	-11.419384	1.147156	-0.628863	H	-5.153605	0.342519	-3.249313
C	7.434414	-0.111819	-0.259483	C	-11.109535	4.429343	1.151530	H	-7.266475	-2.568926	1.594982
C	8.148647	0.518031	-1.271423	C	-10.691179	5.317622	0.149790	H	-9.720606	-0.805997	-2.049897
C	7.466462	-1.315091	1.920991	C	-10.491444	4.865150	-1.153229	H	-8.419432	-1.016771	3.324980
C	6.117063	-1.435651	1.906875	C	-10.706768	3.516989	-1.475187	H	-10.124671	0.756151	3.757447
C	5.324912	-0.910956	0.820981	C	13.868703	0.922629	-1.015148	H	-11.954542	2.328421	2.786764
C	5.972846	-0.246109	-0.269801	C	14.109742	1.624830	0.321985	H	-11.287834	0.797751	-1.655645
C	3.932584	-1.047671	0.827688	C	13.461916	0.970862	1.382055	H	-11.260677	4.782310	2.169271
C	3.120733	-0.556609	-0.201199	C	12.695732	-0.268076	0.913623	H	-10.521152	6.363079	0.393458
C	3.767700	0.107570	-1.291678	C	14.858545	2.770211	0.553823	H	-10.165912	5.558037	-1.924720
C	5.163918	0.243700	-1.296487	C	14.965045	3.265197	1.862701	H	-10.546329	3.164010	-2.491602
C	1.665163	-0.689490	-0.212159	C	14.322205	2.616399	2.914947	H	14.370404	1.421591	-1.848803
C	0.925114	-0.152287	-1.313289	C	13.561947	1.461097	2.676778	H	12.216776	-0.792255	1.742433
C	1.624575	0.506274	-2.384568	C	14.342521	-0.536428	-0.828737	H	15.357611	3.279466	-0.267627
C	2.976728	0.629982	-2.374301	C	13.723718	-1.162527	0.182823	H	15.551222	4.160226	2.053800
C	0.940265	-1.320205	0.805454	C	13.917777	-2.566258	0.672170	H	14.407286	3.005884	3.926000
C	-0.453548	-1.437981	0.777627	C	15.399771	-1.065135	-1.750430	H	13.057643	0.957675	3.498613
C	-1.194651	-0.900989	-0.324284	C	-9.361368	-2.713372	-1.008119	H	12.978777	-3.134978	0.620529
C	-0.472394	-0.271590	-1.339469	C	-8.654306	-3.213045	0.015775	H	14.234179	-2.575187	1.724798
C	-1.153904	-2.097673	1.852929	C	-12.862022	0.929471	-0.121118	H	14.669365	-3.111352	0.094079
C	-2.502902	-2.223330	1.846443	C	-13.053761	1.366234	1.133179	H	15.068652	-1.019295	-2.797488
C	-3.304261	-1.704210	0.764551	C	-10.291847	-3.428936	-1.940416	H	15.667006	-2.102644	-1.530577
C	-2.656274	-1.034376	-0.336657	C	-8.598509	-4.626726	0.511686	H	16.316224	-0.461988	-1.683672
C	-4.688254	-1.845471	0.776980	C	-14.307394	1.325242	1.954363	H	-9.958734	-3.330657	-2.983292
C	-5.505691	-1.352786	-0.260396	C	-13.849265	0.281202	-1.044691	H	-11.303307	-3.000502	-1.891918
C	-4.861483	-0.679595	-1.362832	H	12.152121	1.920603	-3.079922	H	-10.372243	-4.496275	-1.715633
C	-3.457198	-0.545261	-1.361432	H	9.683679	1.706417	-3.131792	H	-8.924128	-4.687991	1.559749
C	-6.923178	-1.478446	-0.293178	H	10.089242	-0.964744	1.737287	H	-7.571259	-5.015889	0.477734
C	-7.654279	-0.963851	-1.346760	H	7.627882	0.942598	-2.124437	H	-9.229754	-5.301897	-0.073117
C	-7.022826	-0.298993	-2.420921	H	8.047651	-1.716428	2.747826	H	-14.151870	0.752319	2.879379
C	-5.652835	-0.164228	-2.426944	H	5.599204	-1.935306	2.722137	H	-15.146808	0.872528	1.418920
C	-7.819510	-2.143585	0.755699	H	3.486386	-1.558772	1.675357	H	-14.610752	2.336933	2.258548
C	-8.805954	-1.064774	1.209047	H	5.609527	0.754802	-2.144466	H	-13.967721	0.866049	-1.967813
C	-9.551396	-0.540127	0.131661	H	1.040258	0.906406	-3.209706	H	-14.838489	0.169867	-0.591724
C	-9.157884	-1.193581	-1.197494	H	3.491174	1.130629	-3.191051	H	-13.506674	-0.718590	-1.348090

19b

C	3.039906	-2.073594	2.486735	C	-12.338974	3.828213	1.089263	H	-7.246896	-0.886175	-2.137913
C	3.639568	-2.592922	1.318844	C	-12.944726	4.929392	0.464814	H	-9.282696	0.893646	2.021369
C	2.913252	-2.768654	0.157568	C	-13.670644	4.759476	-0.712286	H	-9.346882	-0.703471	-3.312903
C	1.532317	-2.431445	0.102048	C	-13.803288	3.485411	-1.285608	H	-11.749539	-0.096617	-3.337852
C	0.918727	-1.904992	1.298592	C	8.245528	0.256405	-1.176320	H	5.656861	-2.899624	2.106469
C	1.704120	-1.738960	2.473075	C	9.026454	0.485498	-0.035503	H	3.233506	-3.490033	-1.905704
C	0.722733	-2.578420	-1.042480	C	8.671366	-0.204893	1.190489	H	6.637491	-0.297835	-3.255443
C	-0.626143	-2.237350	-1.048330	C	7.572604	-1.073582	1.195976	H	4.694440	-1.831765	-3.235110
C	-1.243875	-1.712777	0.145721	C	10.157739	1.358409	-0.001160	H	-11.361509	1.376910	1.981651
C	-0.450016	-1.564668	1.276803	C	10.878021	1.539030	1.158812	H	-13.800073	0.789891	-2.036346

C	-1.421048	-2.402417	-2.240747	C	10.524832	0.869320	2.357409	H	-11.769508	3.962863	2.006183
C	-2.736222	-2.076550	-2.266091	C	9.449626	0.016944	2.368014	H	-12.844414	5.918984	0.902780
C	-3.403899	-1.549165	-1.101229	C	10.715846	2.191248	-1.160744	H	-14.136184	5.616669	-1.191557
C	-2.668092	-1.362795	0.111170	C	10.688679	3.644507	-0.681791	H	-14.367137	3.354188	-2.206506
C	-4.764575	-1.219635	-1.150467	C	11.421163	3.824907	0.502323	H	8.488821	0.763381	-2.105892
C	-5.457527	-0.706172	-0.050592	C	12.042300	2.516263	0.993652	H	7.327684	-1.578605	2.126300
C	-4.721944	-0.520405	1.161961	C	10.048248	4.719106	-1.282926	H	11.108213	1.034106	3.260224
C	-3.361113	-0.849687	1.211096	C	10.144287	5.989987	-0.695060	H	9.169609	-0.505627	3.279583
C	-6.881545	-0.355862	-0.085231	C	10.871143	6.168670	0.480018	H	10.170797	2.057621	-2.096685
C	-7.500168	0.165357	1.109499	C	11.514590	5.079532	1.088116	H	12.616926	2.643490	1.915282
C	-6.705603	0.330075	2.302136	C	-13.807140	0.127936	0.070101	H	9.477473	4.580379	-2.198425
C	-5.389989	0.005732	2.327089	C	-13.108469	0.289484	1.203188	H	9.646894	6.837109	-1.160011
C	-7.674447	-0.499429	-1.217749	C	4.384677	-4.656764	-0.436554	H	10.940465	7.155176	0.930854
C	-9.042974	-0.159125	-1.239155	C	5.087551	-4.485986	0.691158	H	12.079014	5.219268	2.007434
C	-9.659048	0.358584	-0.040509	C	12.906743	1.986012	-0.172613	H	12.153468	0.422723	-2.948105
C	-8.849990	0.503714	1.104320	C	12.208252	1.812289	-1.303861	H	13.761718	1.094681	-2.634946
C	-9.825505	-0.314268	-2.417478	C	12.693188	1.328245	-2.637310	H	12.517538	2.082924	-3.416975
C	-11.160391	0.021361	-2.431547	C	14.365683	1.745801	0.073461	H	14.514291	1.009170	0.875617
C	-11.766740	0.520638	-1.257601	C	4.132492	-5.920160	-1.202624	H	14.866432	2.669017	0.397681
C	-11.043337	0.686866	-0.092099	C	5.820684	-5.510173	1.503720	H	14.886156	1.379362	-0.815995
C	5.102946	-3.016197	1.173411	C	-15.032555	-0.702962	-0.163935	H	3.055528	-6.124590	-1.281815
C	5.667042	-2.182596	0.016433	C	-13.359590	-0.317130	2.550983	H	4.603317	-6.791516	-0.738478
C	4.943307	-2.359150	-1.140944	H	3.632666	-1.940074	3.388485	H	4.514656	-5.841216	-2.230245
C	3.779056	-3.338088	-0.972669	H	1.228564	-1.339292	3.365474	H	5.429376	-5.553009	2.530042
C	6.792677	-1.304009	0.054463	H	1.155271	-2.970168	-1.958934	H	6.887792	-5.259672	1.583593
C	7.145236	-0.610828	-1.170717	H	-0.876206	-1.172118	2.195134	H	5.745505	-6.514391	1.076794
C	6.362326	-0.826530	-2.345993	H	-0.933508	-2.798615	-3.128378	H	-14.854915	-1.455893	-0.944721
C	5.285451	-1.677582	-2.335427	H	-3.319649	-2.207521	-3.174378	H	-15.868673	-0.080142	-0.511972
C	-11.905337	1.247688	1.044301	H	-5.280282	-1.378598	-2.092646	H	-15.361149	-1.228516	0.737179
C	-12.475343	2.568438	0.522681	H	-2.845456	-0.690872	2.153315	H	-12.495694	-0.909395	2.883753
C	-13.206978	2.397521	-0.663324	H	-7.193381	0.725698	3.189871	H	-14.235330	-0.972295	2.559005
C	-13.230906	0.936300	-1.114241	H	-4.806532	0.137242	3.235270	H	-13.518180	0.462527	3.309408

19c

C	-2.298652	1.839199	-2.477863	C	8.870620	-1.366756	2.570849	H	-7.477920	1.154635	3.008063
C	-3.136128	1.816622	-1.329352	C	10.111224	-1.965453	2.571819	H	-5.268904	2.228380	3.040667
C	-2.684360	2.438771	-0.108552	C	10.897012	-1.942471	1.399705	H	-6.523805	-0.149311	-2.318213
C	-1.405206	3.059135	-0.113230	C	10.442134	-1.335409	0.243560	H	-9.197775	-0.049826	1.857080
C	-0.621869	3.060056	-1.251676	C	12.286830	-2.559292	1.240909	H	-8.256316	-1.373795	-3.468064
C	-1.062437	2.446942	-2.443541	C	12.171978	-3.553057	0.084178	H	-10.487753	-2.452998	-3.467892
C	-0.714780	3.777436	1.052063	C	11.713409	-2.938387	-1.091868	H	7.477893	1.154586	-3.008084
C	0.621868	3.060051	1.251678	C	11.457980	-1.442141	-0.899114	H	5.268885	2.228348	-3.040680
C	1.405204	3.059132	0.113232	C	-12.286812	-2.559326	-1.240908	H	6.523837	-0.149250	2.318229
C	0.714780	3.777439	-1.052058	C	-12.171974	-3.553060	-0.084149	H	9.197757	-0.049859	-1.857098
C	1.062435	2.446933	2.443541	C	-11.713426	-2.938358	1.091887	H	8.256361	-1.373709	3.468088
C	2.298650	1.839188	2.477861	C	-11.457993	-1.442116	0.899096	H	10.487799	-2.452912	3.467913
C	3.136126	1.816614	1.329350	C	-12.460616	-4.910557	-0.102411	H	12.645704	-3.025925	2.162389
C	2.684358	2.438766	0.108551	C	-12.293477	-5.661947	1.070985	H	11.129703	-0.959517	-1.821283
C	4.404491	1.195795	1.356463	C	-11.838339	-5.052182	2.238253	H	-12.645674	-3.025984	-2.162380
C	5.245803	1.154146	0.253273	C	-11.542836	-3.680401	2.252503	H	-11.129729	-0.959467	1.821256
C	4.788907	1.776579	-0.965444	C	12.460623	-4.910553	0.102471	H	-12.812301	-5.388081	-1.014267
C	3.543892	2.392270	-1.009734	C	12.293467	-5.661974	-1.070902	H	-12.519291	-6.725058	1.066322
C	-4.404493	1.195802	-1.356466	C	11.838309	-5.052242	-2.238179	H	-11.709286	-5.640121	3.143162
C	-5.245804	1.154149	-0.253276	C	11.542803	-3.680462	-2.252461	H	-11.183998	-3.206378	3.163386
C	-4.788913	1.776588	0.965439	C	0.379081	5.202145	-0.552430	H	12.812322	-5.388052	1.014335
C	-3.543898	2.392279	1.009731	C	-0.379078	5.202144	0.552440	H	12.519284	-6.725085	-1.066214
C	-6.568848	0.509970	-0.272335	C	-12.781915	-0.840742	0.373269	H	11.709243	-5.640206	-3.143070
C	-7.369421	0.521710	0.927547	C	-13.219457	-1.429510	-0.749296	H	11.183949	-3.206464	-3.163351
C	-6.854706	1.161447	2.116857	C	12.781911	-0.840753	-0.373324	H	-12.731785	1.138080	1.220924
C	-5.637813	1.752924	2.134826	C	13.219466	-1.429487	0.749255	H	-13.633207	-0.035798	2.179143
C	-7.093914	-0.115760	-1.394624	C	-13.411137	0.277158	1.149103	H	-14.344648	0.626277	0.698694
C	-8.365099	-0.731064	-1.403850	C	-14.458325	-1.134190	-1.539752	H	-14.208724	-0.849260	-2.571562
C	-9.163469	-0.713626	-0.202584	C	0.909739	6.371538	-1.325506	H	-15.050534	-0.324278	-1.104403
C	-8.623275	-0.077642	0.935360	C	-0.909734	6.371534	1.325522	H	-15.102854	-2.022078	-1.605862
C	-8.870586	-1.366820	-2.570834	C	14.458344	-1.134145	1.539687	H	0.550431	6.352956	-2.364165
C	-10.111190	-1.965517	-2.571805	C	13.411124	0.277123	-1.149199	H	2.007559	6.347956	-1.371339
C	-10.896994	-1.942506	-1.399702	H	-2.651983	1.364191	-3.389919	H	0.615323	7.330018	-0.888567
C	-10.442130	-1.335417	-0.243566	H	-0.427776	2.456105	-3.326423	H	-0.550436	6.352941	2.364183
C	6.568850	0.509975	0.272332	H	-1.309663	3.795989	1.966886	H	-2.007555	6.347961	1.371344

C	7.369412	0.521693	-0.927557	H	1.309663	3.795995	-1.966881	H	-0.615308	7.330016	0.888593
C	6.854687	1.161415	-2.116872	H	0.427774	2.456092	3.326423	H	14.208757	-0.849201	2.571496
C	5.637799	1.752900	-2.134836	H	2.651981	1.364177	3.389916	H	15.050539	-0.324234	1.104316
C	7.093932	-0.115725	1.394631	H	4.709258	0.738987	2.293252	H	15.102882	-2.022026	1.605799
C	8.365118	-0.731028	1.403856	H	3.232396	2.850224	-1.944512	H	12.731773	1.138044	-1.221035
C	9.163473	-0.713618	0.202579	H	-4.709261	0.738999	-2.293257	H	13.633177	-0.035863	-2.179233
C	8.623266	-0.077658	-0.935372	H	-3.232405	2.850237	1.944508	H	14.344642	0.626252	-0.698814

20a

C	11.831768	1.390899	-2.343251	C	-8.835402	-0.255589	2.548681	H	8.243623	-1.319074	2.909628
C	12.555726	0.950300	-1.212431	C	-9.805502	0.748248	2.713254	H	5.795711	-1.543635	2.894578
C	11.911792	0.396984	-0.120479	C	-10.535838	1.181813	1.617543	H	3.685154	-1.254016	1.815780
C	10.494305	0.255856	-0.106563	C	-10.319372	0.629239	0.335409	H	5.816497	0.748865	-2.171526
C	9.758338	0.707152	-1.262585	C	-11.623263	2.258891	1.625147	H	1.249897	0.814224	-3.255538
C	10.461620	1.270225	-2.364204	C	-11.187792	3.303685	0.595898	H	3.700677	1.039113	-3.249584
C	9.762470	-0.296278	0.963658	C	-10.973992	2.755264	-0.680342	H	1.658035	-1.441754	1.810083
C	8.376232	-0.414424	0.934534	C	-11.231462	1.247433	-0.731223	H	-0.782967	0.143236	-2.187717
C	7.636924	0.036304	-0.218909	C	-10.999955	4.662646	0.815615	H	-0.369559	-2.113480	2.877645
C	8.353726	0.581147	-1.277533	C	-10.595760	5.481601	-0.247401	H	-2.817375	-2.339837	2.879842
C	7.664305	-0.986278	2.051711	C	-10.384187	4.937620	-1.513611	H	-4.942955	-2.048685	1.809030
C	6.315117	-1.110015	2.043338	C	-10.573739	3.566769	-1.734641	H	-2.801942	-0.040528	-2.189416
C	5.525467	-0.675262	0.916816	C	14.076583	1.024262	-1.054229	H	-7.408565	0.012596	-3.235722
C	6.175586	-0.099422	-0.222141	C	14.326750	1.812401	0.233708	H	-4.943074	0.249676	-3.262367
C	4.133155	-0.812121	0.931012	C	13.676710	1.250560	1.345108	H	-7.055899	-2.238408	1.815781
C	3.323812	-0.405199	-0.135739	C	12.884488	-0.012046	0.993867	H	-9.514000	-0.759870	-1.992636
C	3.973023	0.170524	-1.274177	C	15.095636	2.960328	0.375399	H	-8.259241	-0.601868	3.403308
C	5.369115	0.306564	-1.286646	C	15.215097	3.554348	1.639619	H	-9.980516	1.180976	3.695259
C	1.868213	-0.539040	-0.139415	C	14.570286	2.996977	2.742740	H	-11.787816	2.684729	2.618417
C	1.130610	-0.090452	-1.281181	C	13.796358	1.836987	2.598553	H	-11.077904	0.831041	-1.729064
C	1.832361	0.481017	-2.399950	C	14.538842	-0.405878	-0.765320	H	-11.164855	5.087383	1.803173
C	3.184452	0.605103	-2.396665	C	13.888858	-0.969057	0.345432	H	-10.447021	6.545299	-0.081739
C	1.141366	-1.087794	0.923193	C	14.188198	-2.263025	0.752097	H	-10.070823	5.577572	-2.334149
C	-0.252479	-1.207113	0.901926	C	15.143789	-3.002347	0.041083	H	-10.408897	3.143063	-2.722765
C	-0.991072	-0.757858	-0.240065	C	15.788620	-2.443204	-1.061134	H	14.571569	1.463442	-1.924329
C	-0.266835	-0.210856	-1.300575	C	15.487465	-1.136279	-1.469342	H	12.397528	-0.451308	1.865754
C	-0.955121	-1.780516	2.024025	C	-9.157585	-2.581550	-0.823016	H	15.599549	3.394888	-0.484885
C	-2.304237	-1.905313	2.025287	C	-8.420353	-3.018415	0.289660	H	15.813599	4.453699	1.757765
C	-3.103028	-1.470761	0.905010	C	-12.662516	1.045954	-0.227367	H	14.666387	3.462202	3.720133
C	-2.452705	-0.890338	-0.244048	C	-12.875446	1.595473	1.048545	H	13.294385	1.402318	3.459916
C	-4.487699	-1.607354	0.927110	C	-9.955469	-3.471136	-1.530916	H	13.686277	-2.699710	1.612468
C	-5.302671	-1.195085	-0.145990	C	-10.016711	-4.811205	-1.124002	H	15.381227	-4.016070	0.352459
C	-4.655792	-0.614332	-1.297443	C	-9.285418	-5.245049	-0.019284	H	16.528476	-3.021366	-1.608251
C	-3.251689	-0.482437	-1.305459	C	-8.482063	-4.345499	0.695240	H	15.991358	-0.700032	-2.328785
C	-6.722064	-1.314833	-0.166947	C	-14.122760	1.501640	1.653298	H	-10.526997	-3.132985	-2.392302
C	-7.450746	-0.885340	-1.260970	C	-15.166896	0.854351	0.978867	H	-10.637773	-5.513323	-1.673807
C	-6.813884	-0.317436	-2.387456	C	-14.955795	0.309687	-0.287136	H	-9.336748	-6.285192	0.291261
C	-5.444697	-0.186073	-2.401733	C	-13.697388	0.404504	-0.896594	H	-7.912711	-4.684592	1.557624
C	-7.610051	-1.895098	0.940944	H	12.360977	1.822403	-3.189280	H	-14.287770	1.926466	2.640810
C	-8.619070	-0.797778	1.291377	H	9.893350	1.605700	-3.228346	H	-16.144830	0.777757	1.446654
C	-9.360690	-0.358410	0.173844	H	10.285859	-0.646077	1.848840	H	-15.769200	-0.191131	-0.805382
C	-8.960117	-1.091803	-1.112021	H	7.835583	0.932128	-2.164826	H	-13.533411	-0.020600	-1.884241

20b

C	-2.879973	-1.937533	-2.484712	C	12.539107	3.944558	-1.021734	H	1.004556	-2.523666	3.206794
C	-3.504257	-2.411777	-1.309199	C	13.156957	5.051128	-0.422516	H	3.392865	-1.943173	3.271816
C	-2.796999	-2.555080	-0.130613	C	13.866082	4.901811	0.768362	H	5.372685	-1.150531	2.197724
C	-1.412499	-2.228102	-0.066726	C	13.967999	3.643648	1.378271	H	3.001723	-0.561407	-2.099278
C	-0.778130	-1.740778	-1.268026	C	-8.203367	0.435455	1.055393	H	7.369772	0.812791	-3.107834
C	-1.544375	-1.608157	-2.459888	C	-8.968120	0.630544	-0.102268	H	4.981686	0.231313	-3.173342
C	-0.620828	-2.348419	1.092567	C	-8.582740	-0.078939	-1.306947	H	7.342388	-0.671830	2.256905
C	0.730215	-2.014832	1.108726	C	-7.469237	-0.928132	-1.278684	H	9.441056	1.004118	-1.913627
C	1.367705	-1.525689	-0.089418	C	-10.110201	1.488610	-0.171851	H	9.427477	-0.476704	3.453925
C	0.591272	-1.405895	-1.236104	C	-10.816571	1.631348	-1.347303	H	11.832313	0.116991	3.497208
C	1.506742	-2.152707	2.316502	C	-10.436807	0.935536	-2.523516	H	-5.509264	-2.715330	-2.137806
C	2.823129	-1.832718	2.352222	C	-9.347493	0.102968	-2.500037	H	-3.139152	-3.197898	1.959698
C	3.509960	-1.338792	1.184011	C	-10.691300	2.329543	0.973194	H	-6.628811	-0.060514	3.173274
C	2.792675	-1.180681	-0.043420	C	-10.716895	3.773791	0.464361	H	-4.664626	-1.565533	3.216061
C	4.871270	-1.013820	1.244431	C	-11.434043	3.915591	-0.735200	H	11.500809	1.504956	-1.858930
C	5.581826	-0.531661	0.141734	C	-12.006888	2.587944	-1.237199	H	13.897263	0.984944	2.232960
C	4.864561	-0.373468	-1.085667	C	-10.129074	4.879357	1.065454	H	11.986880	4.061784	-1.951333

C	3.503204	-0.698480	-1.146077	C	-10.255976	6.137951	0.460964	H	13.081174	6.029043	-0.890395
C	7.006678	-0.186487	0.187786	C	-10.967587	6.278304	-0.729443	H	14.342815	5.763419	1.228099
C	7.643987	0.303551	-1.009865	C	-11.563023	5.162120	-1.333793	H	14.522139	3.527274	2.306848
C	6.867618	0.441395	-2.217719	C	13.929664	0.260260	0.156298	H	-8.470031	0.954015	1.971823
C	5.551339	0.120846	-2.253703	C	13.215011	0.409120	-1.043936	H	-7.202777	-1.447116	-2.195044
C	7.783455	-0.306997	1.334238	C	-4.252610	-4.412259	0.513037	H	-11.012361	1.067226	-3.436491
C	9.152587	0.028492	1.366044	C	-4.968161	-4.266177	-0.685927	H	-9.046365	-0.435776	-3.395218
C	9.786677	0.518185	0.165808	C	-12.873999	2.050383	-0.096158	H	-10.147182	2.231849	1.913655
C	8.994869	0.638365	-0.993288	C	-12.157645	1.907423	1.103755	H	-12.556337	2.690157	-2.176708
C	9.919179	-0.106391	2.557627	C	-12.789479	1.432915	2.246013	H	-9.574629	4.770994	1.994811
C	11.254355	0.223015	2.582469	C	-14.148922	1.095013	2.188440	H	-9.796296	7.006755	0.924491
C	11.878629	0.702890	1.408776	C	-14.859488	1.237044	0.997617	H	-11.062427	7.256534	-1.193229
C	11.171104	0.848793	0.229416	C	-14.221405	1.718818	-0.154067	H	-12.119180	5.272119	-2.261952
C	-4.975658	-2.820701	-1.192148	C	-4.137228	-5.657674	1.115818	H	-12.236167	1.322968	3.175859
C	-5.561414	-1.978621	-0.050523	C	-4.743699	-6.769327	0.513039	H	-14.648900	0.720705	3.077772
C	-4.856753	-2.123839	1.124439	C	-5.453886	-6.624353	-0.677346	H	-15.913104	0.973499	0.959283
C	-3.670757	-3.086848	1.013443	C	-5.568792	-5.365459	-1.284709	H	-14.776353	1.830661	-1.082729
C	-6.704256	-1.122759	-0.120997	C	15.032061	-0.582317	0.217916	H	-3.583027	-5.771682	2.044628
C	-7.090227	-0.414461	1.084187	C	15.423663	-1.284896	-0.930296	H	-4.658035	-7.747578	0.978478
C	-6.327007	-0.598363	2.277831	C	14.714734	-1.137339	-2.121505	H	-5.921791	-7.489597	-1.139256
C	-5.238124	-1.431996	2.302086	C	13.603597	-0.284736	-2.182749	H	-6.122985	-5.253216	-2.213733
C	12.039384	1.382340	-0.918110	H	-3.457001	-1.833959	-3.400296	H	15.585913	-0.696949	1.146879
C	12.638611	2.698868	-0.415350	H	-1.052503	-1.239701	-3.356819	H	16.284163	-1.947348	-0.889061
C	13.353472	2.548693	0.784610	H	-1.067489	-2.713563	2.013009	H	15.022881	-1.684910	-3.008174
C	13.351148	1.105319	1.293696	H	1.032814	-1.042347	-2.159040	H	13.051659	-0.169437	-3.112761

20c

C	-2.328435	1.621825	-2.477311	C	8.928661	-1.523209	2.559106	H	0.459394	2.231508	3.332400
C	-3.160443	1.598808	-1.324171	C	10.173876	-2.109573	2.558317	H	2.691190	1.157443	3.391084
C	-2.697362	2.209147	-0.102587	C	10.952940	-2.094213	1.380484	H	4.746571	0.540962	2.288039
C	-1.408781	2.812994	-0.106305	C	10.487292	-1.498632	0.221314	H	3.235441	2.619077	-1.953438
C	-0.630987	2.814059	-1.249751	C	12.349200	-2.705243	1.236838	H	-4.746587	0.541017	-2.288045
C	-1.087980	2.218058	-2.445414	C	12.258479	-3.690002	0.068763	H	-3.235472	2.619127	1.953439
C	-0.718479	3.515705	1.070227	C	11.789317	-3.087172	-1.110207	H	-7.493128	0.962381	3.024254
C	0.630982	2.814036	1.249772	C	11.490474	-1.595411	-0.936054	H	-5.274368	2.015468	3.053103
C	1.408776	2.812981	0.106326	C	-12.349136	-2.705366	-1.236818	H	-6.569478	-0.331089	-2.309794
C	0.718479	3.515715	-1.070195	C	-12.258482	-3.690005	-0.068637	H	-9.225631	-0.224638	1.876165
C	1.087971	2.218016	2.445427	C	-11.789381	-3.087056	1.110297	H	-8.319218	-1.528871	-3.459556
C	2.328425	1.621777	2.477317	C	-11.490523	-1.595313	0.936006	H	-10.561026	-2.584083	-3.456656
C	3.160434	1.598775	1.324178	C	-12.583851	-5.040138	-0.086036	H	7.493029	0.962196	-3.024314
C	2.697353	2.209126	0.102600	C	-12.437499	-5.796335	1.085334	H	5.274302	2.015352	-3.053132
C	4.434031	0.989920	1.350114	C	-11.972044	-5.198298	2.255393	H	6.569607	-0.330837	2.309880
C	5.271707	0.951409	0.243734	C	-11.646140	-3.834919	2.271997	H	9.225561	-0.224772	-1.876216
C	4.805012	1.564624	-0.975207	C	12.583842	-5.040134	0.086285	H	8.319403	-1.528510	3.459670
C	3.553173	2.167433	-1.017956	C	12.437424	-5.796452	-1.085000	H	10.561210	-2.583725	3.456760
C	-4.434039	0.989950	-1.350112	C	11.971909	-5.198533	-2.255095	H	12.703766	-3.171777	2.159660
C	-5.271710	0.951420	-0.243729	C	11.646010	-3.835155	-2.271822	H	11.143672	-1.131859	-1.860803
C	-4.805032	1.564659	0.975207	C	0.393546	4.930073	-0.581242	H	-12.703654	-3.171993	-2.159611
C	-3.553191	2.167466	1.017962	C	-0.393534	4.930068	0.581296	H	-11.143770	-1.131667	1.860726
C	-6.600384	0.319576	-0.260535	C	-12.788336	-0.966077	0.421504	H	-12.947735	-5.506832	-0.998535
C	-7.396308	0.334115	0.941729	C	-13.256782	-1.570298	-0.757063	H	-12.688455	-6.853682	1.078734
C	-6.872557	0.965569	2.131286	C	12.788317	-0.966126	-0.421688	H	-11.860295	-5.789805	3.160164
C	-5.650133	1.545739	2.147194	C	13.256824	-1.570228	0.756916	H	-11.283905	-3.368943	3.185518
C	-7.135189	-0.296847	-1.383631	C	-13.491593	0.083012	0.999619	H	12.947773	-5.506736	0.998811
C	-8.411552	-0.900258	-1.389794	C	-14.672316	0.534538	0.393559	H	12.688376	-6.853800	-1.078305
C	-9.204221	-0.882802	-0.185580	C	-15.136831	-0.065184	-0.776009	H	11.860108	-5.790133	-3.159800
C	-8.655259	-0.255328	0.952239	C	-14.427530	-1.125452	-1.357459	H	11.283727	-3.369271	-3.185372
C	-8.928523	-1.523478	-2.559025	C	0.787439	6.127438	-1.163648	H	-13.130416	0.550907	1.912575
C	-10.173739	-2.109840	-2.558242	C	0.390693	7.337828	-0.577039	H	-15.226329	1.356299	0.839387
C	-10.952867	-2.094356	-1.380452	C	-0.390661	7.337822	0.577130	H	-16.052776	0.289348	-1.241155
C	-10.487278	-1.498657	-0.221318	C	-0.787417	6.127426	1.163721	H	-14.790621	-1.593732	-2.269457
C	6.600395	0.319595	0.260545	C	14.427607	-1.125326	1.357203	H	1.397086	6.128296	-2.064285
C	7.396273	0.334047	-0.941751	C	15.136881	-0.065120	0.775606	H	0.695172	8.279204	-1.026528
C	6.872490	0.965443	-2.131324	C	14.672305	0.534484	-0.393997	H	-0.695132	8.279194	1.026633
C	5.650082	1.545650	-2.147215	C	13.491547	0.082901	-0.999947	H	-1.397064	6.128276	2.064357
C	7.135262	-0.296705	1.383678	H	-2.691203	1.157501	-3.391083	H	14.790745	-1.593514	2.269230
C	8.411628	-0.900111	1.389838	H	-0.459403	2.231559	-3.332387	H	16.052852	0.289456	1.240667
C	9.204235	-0.882775	0.185582	H	-1.306925	3.519273	1.988935	H	15.226297	1.356197	-0.839940
C	8.655223	-0.255397	-0.952267	H	1.306927	3.519294	-1.988902	H	13.130322	0.550704	-1.912932

5. References

[S1] K. Baumgärtner, A. L. Meza Chincha, A. Dreuw, F. Rominger, M. Mastalerz, *Angew. Chem. Int. Ed.* **2016**, *55*, 15594-15598.

[S2] G. M. Sheldrick, *Acta Crystallogr.* **2015**, *A71*, 3-8.

[S3] G. M. Sheldrick, *Acta Crystallogr.* **2015**, *C71*, 3-8.

[S4] O. V. Dolomanov, L. J. Bourhis, R. J. Gildea, J. A. K. Howard, H. Puschmann, *J. Appl. Crystallogr.* **2009**, *42*, 339-341.

[S5] M. J. Frisch, G. W. Trucks, H. B. Schlegel, G. E. Scuseria, M. A. Robb, J. R. Cheeseman, G. Scalmani, V. Barone, G. A. Petersson, H. Nakatsuji, X. Li, M. Caricato, A. V. Marenich, J. Bloino, B. G. Janesko, R. Gomperts, B. Mennucci, H. P. Hratchian, J. V. Ortiz, A. F. Izmaylov, J. L. Sonnenberg, D. Williams-Young, F. Ding, F. Lipparini, F. Egidi, J. Goings, B. Peng, A. Petrone, T. Henderson, D. Ranasinghe, V. G. Zakrzewski, J. Gao, N. Rega, G. Zheng, W. Liang, M. Hada, M. Ehara, K. Toyota, R. Fukuda, J. Hasegawa, M. Ishida, T. Nakajima, Y. Honda, O. Kitao, H. Nakai, T. Vreven, K. Throssell, J. A. Montgomery, Jr., J. E. Peralta, F. Ogliaro, M. J. Bearpark, J. J. Heyd, E. N. Brothers, K. N. Kudin, V. N. Staroverov, T. A. Keith, R. Kobayashi, J. Normand, K. Raghavachari, A. P. Rendell, J. C. Burant, S. S. Iyengar, J. Tomasi, M. Cossi, J. M. Millam, M. Klene, C. Adamo, R. Cammi, J. W. Ochterski, R. L. Martin, K. Morokuma, O. Farkas, J. B. Foresman, D. J. Fox, Gaussian 16, Revision B.01, Gaussian, Inc., Wallingford CT, 2016.

[S6] A. D. Becke, *J. Chem. Phys.* **1993**, *98*, 5648-5652.

[S7] C. Lee, W. Yang, R. G. Parr, *Phys. Rev. B* **1988**, *37*, 785-789.

6. ^{1}H NMR and ^{13}C NMR Spectra of Products

^{1}H NMR spectrum of **2** (600 MHz, CD_2Cl_2)

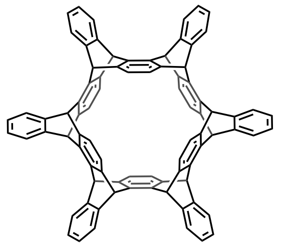

2

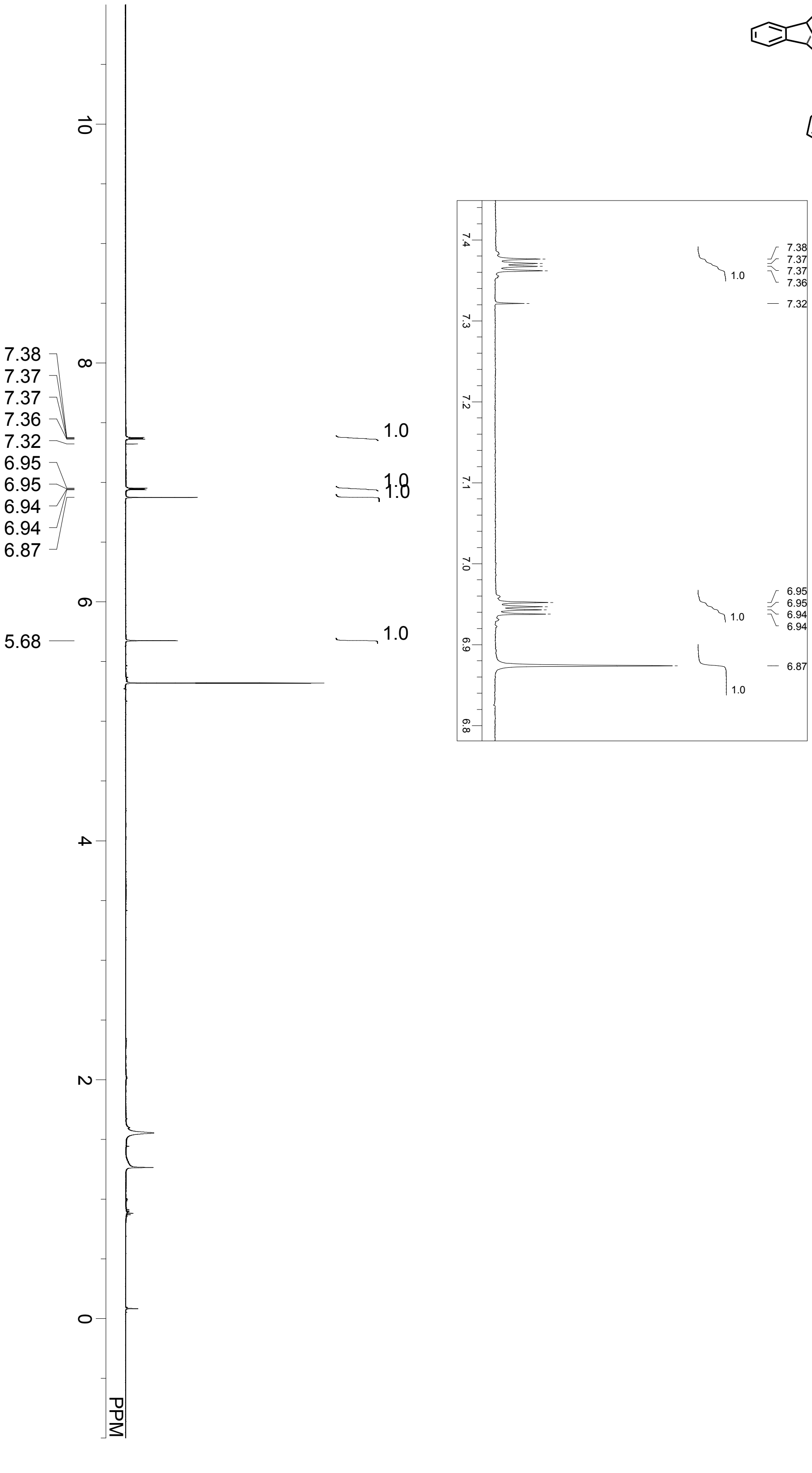

^{13}C NMR spectrum of **2** (150 MHz, CD_2Cl_2)

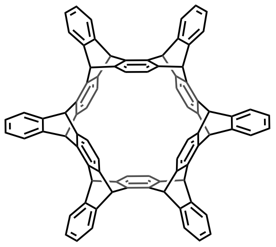

2

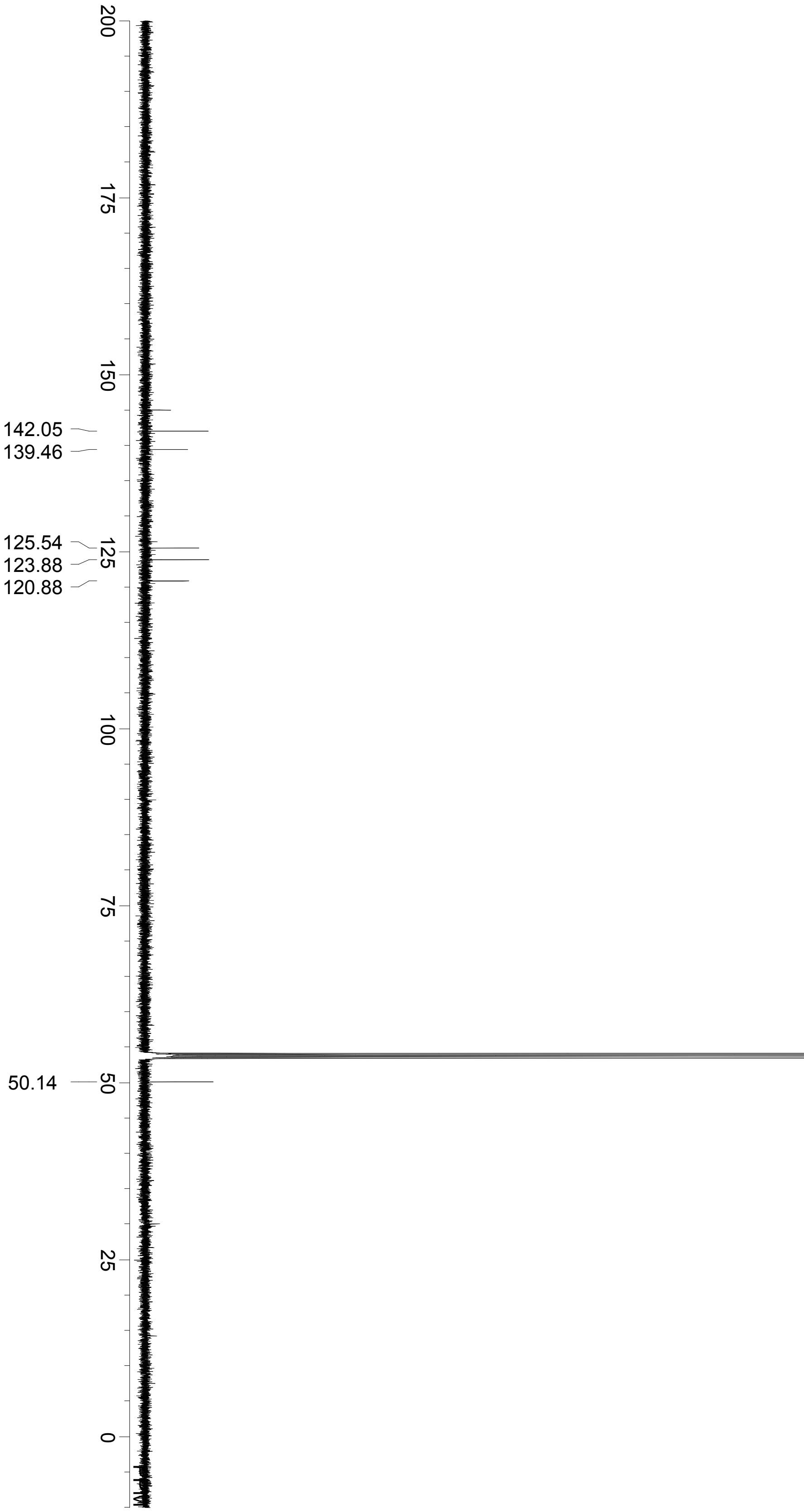

^{1}H NMR spectrum of **3** (600 MHz, CD_2Cl_2)

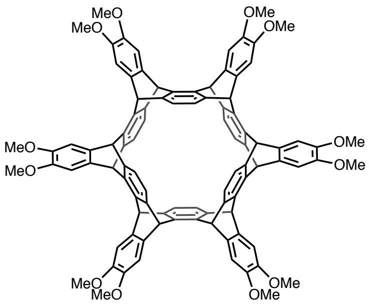

3

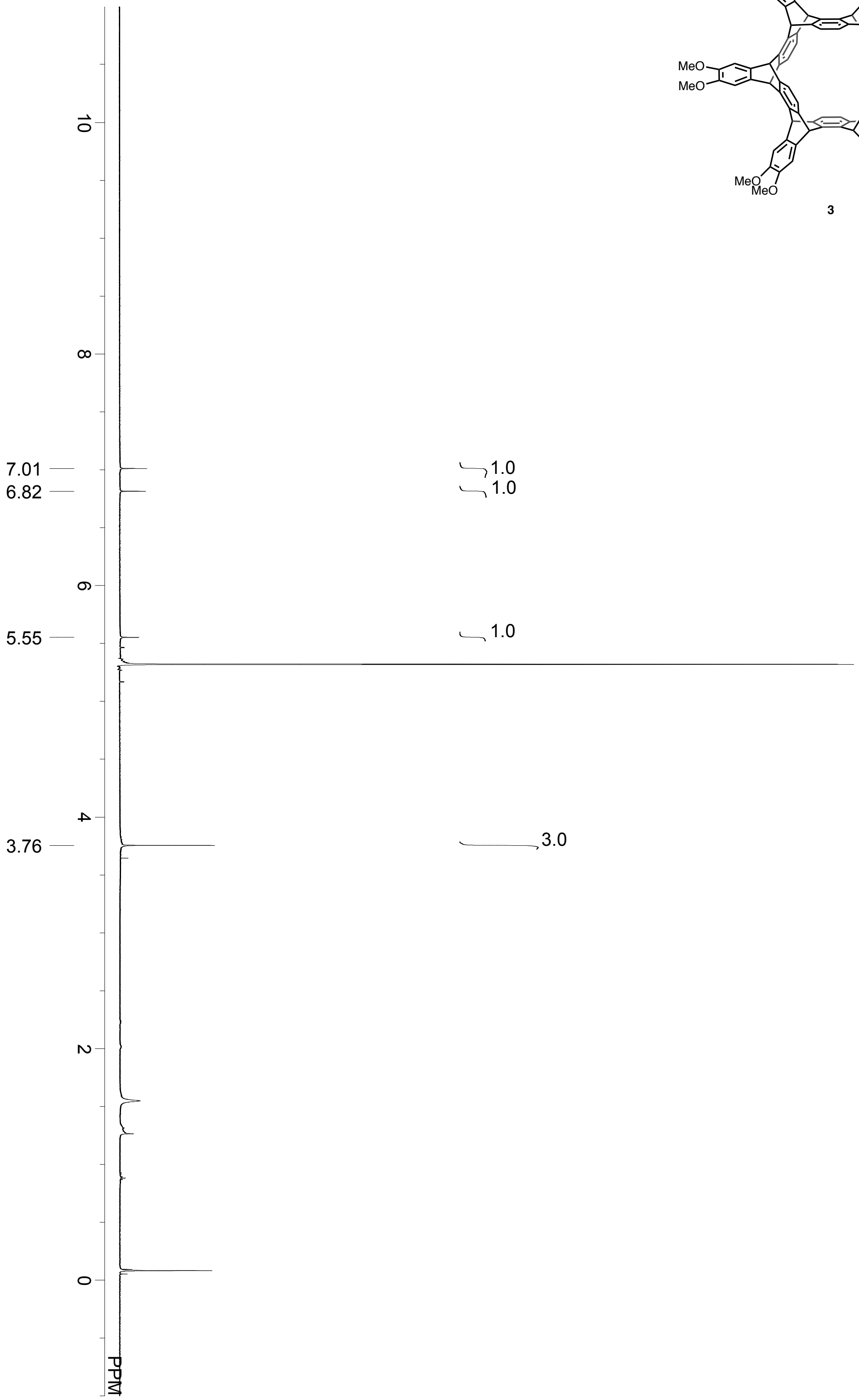

^{1}H NMR spectrum of **4** (600 MHz, CD_2Cl_2)

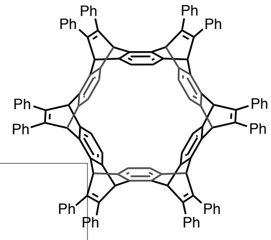

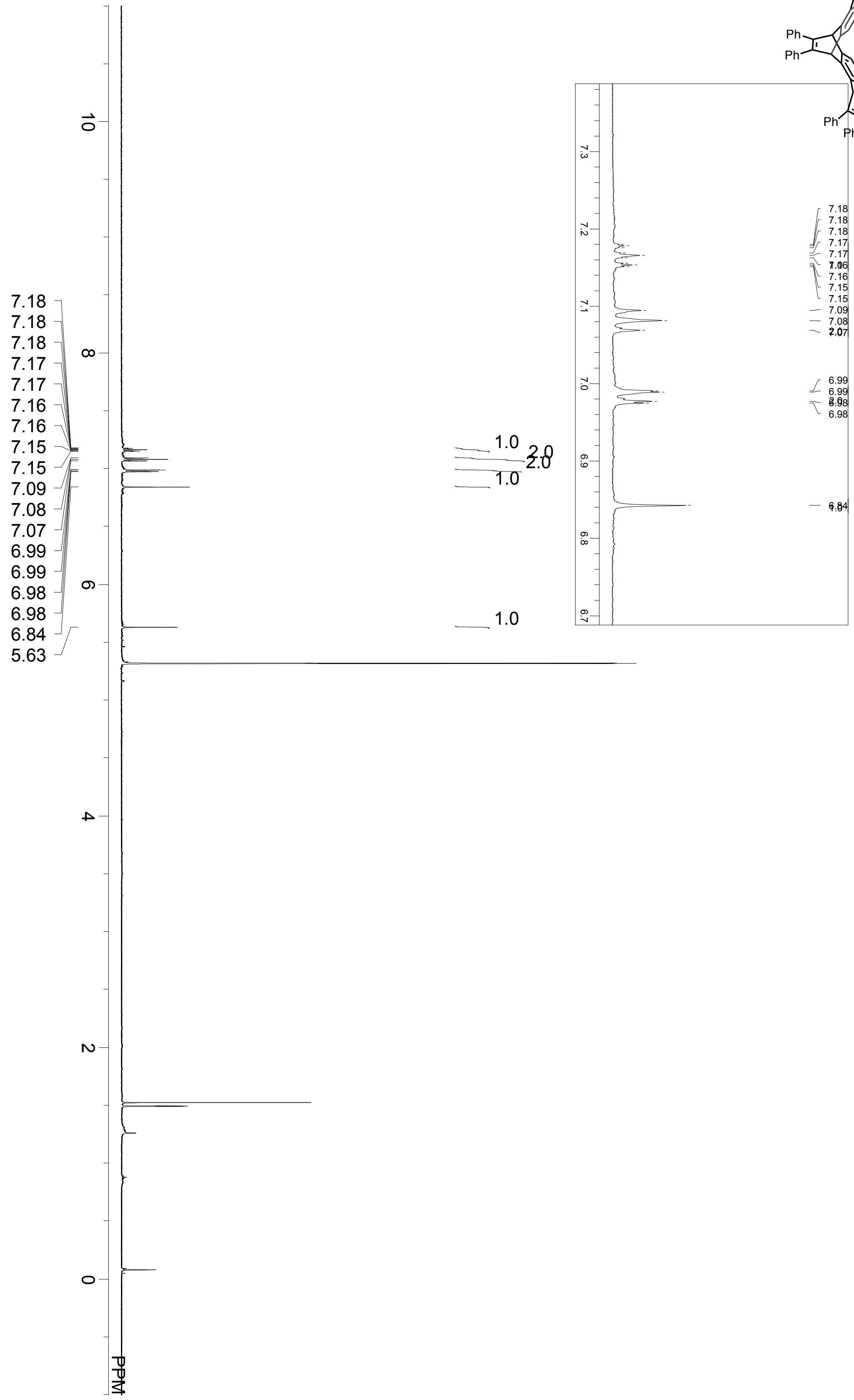

^{13}C NMR spectrum of **4** (150 MHz, CD_2Cl_2)

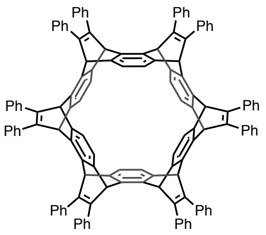

4

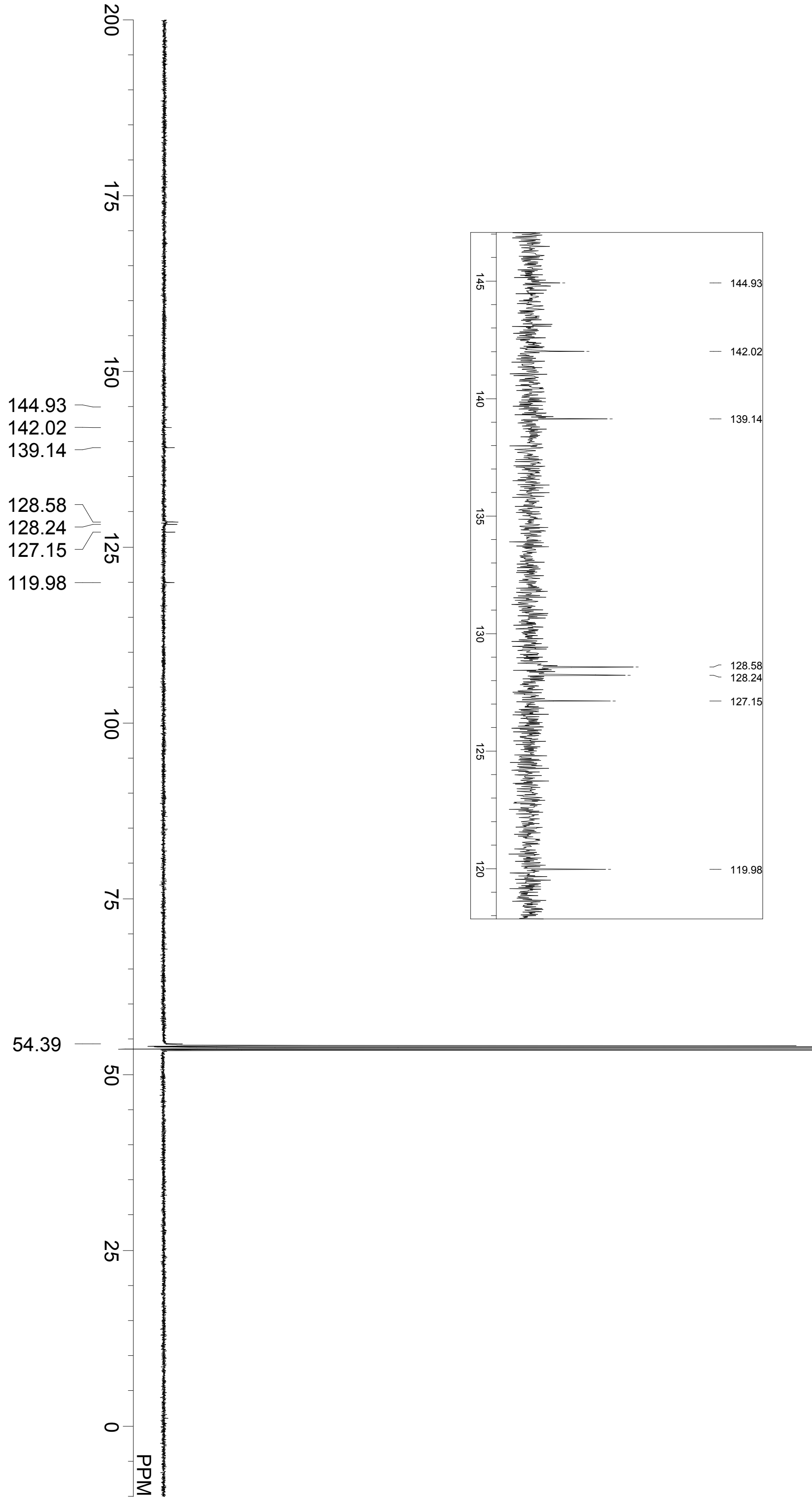

^{1}H NMR spectrum of **12** (600 MHz, CD_2Cl_2)

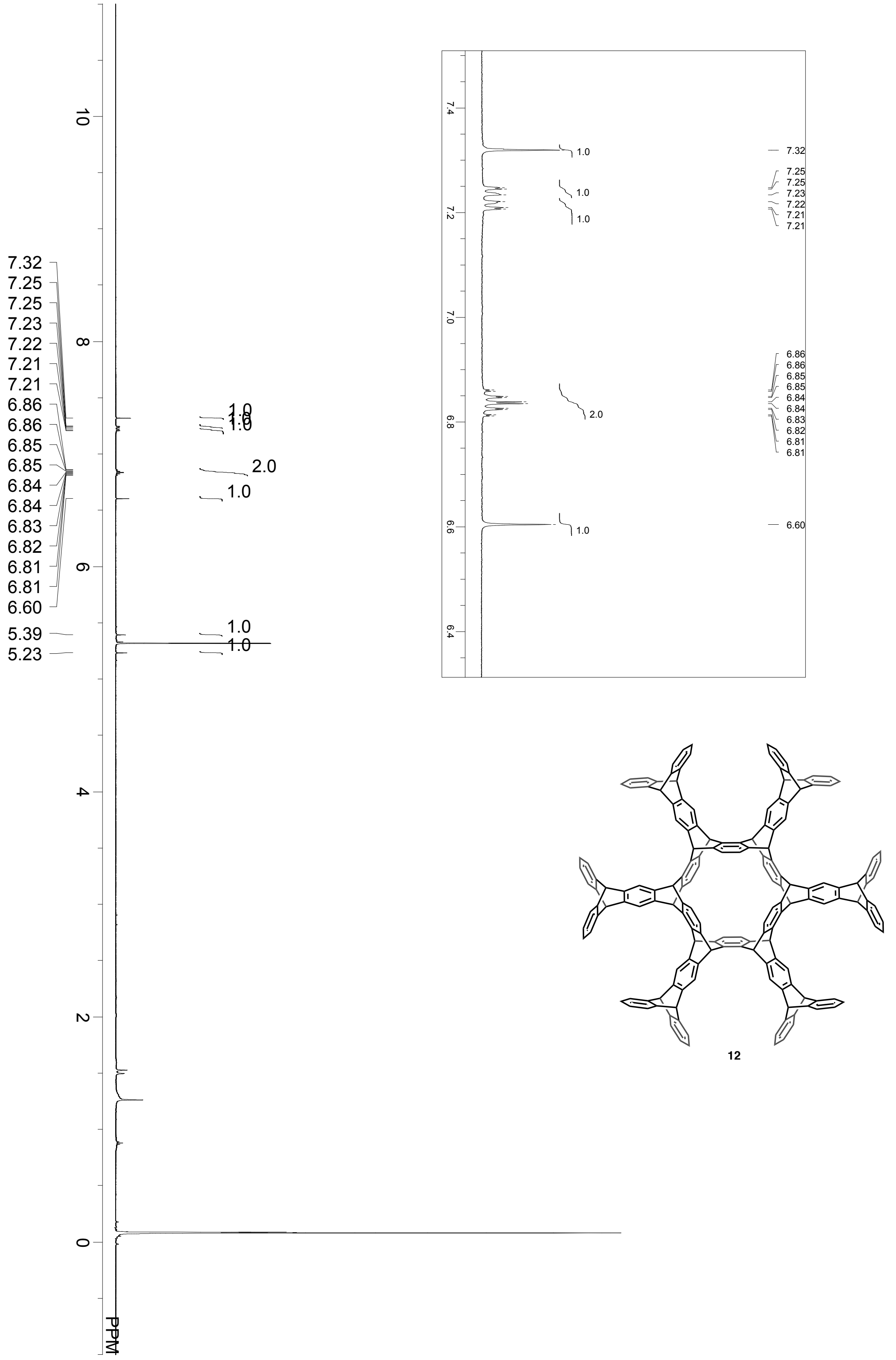

Supplement: Supplementary file 1 [file SC-011-D0SC02501A-s001.pdf]
